# MOF MIL-53(Al) catalyzed hydroacylation of azodicarboxylates: sustainable catalyst for heterogeneous acylhydrazide synthesis

**DOI:** 10.1039/d5ra07909h

**Published:** 2025-12-10

**Authors:** Reynier Báez, Lucas Marchini, José A. C. Delgado, Sebastián San Martín, Felipe Verdugo, Kelvin C. Araújo, Ernesto C. Pereira, Rajender S. Varma, Marcio W. Paixão, Claudio A. Jiménez

**Affiliations:** a Department of Organic Chemistry, Faculty of Chemical Sciences, Universidad de Concepción Concepción 4130000 Chile cjimenez@udec.cl; b Laboratory for Sustainable Organic Synthesis and Catalysis, Department of Chemistry, Federal University of São Carlos Rodovia Washington Luís, km 235 – SP310 São Carlos São Paulo 13565-905 Brazil mwpaixao@ufscar.br; c CDMF, LIEC, Department of Chemistry, Federal University of São Carlos Rodovia Washington Luís, km 235 – SP310 São Carlos São Paulo 13565-905 Brazil

## Abstract

This study reports one of the few examples of acylhydrazide synthesis catalyzed by the metal–organic framework (MOF) MIL-53(Al) *via* the aldehyde C(sp^2^)–H hydrazidation reaction. The material exhibited high catalytic performance, displaying a broad substrate scope and affording yields of up to 97%. The catalyst retained its structural integrity and activity for at least four consecutive cycles, with no significant metal leaching. Notably, MIL-53(Al) was synthesized from waste-derived materials and thoroughly characterized to confirm its crystallinity, structural integrity, and porosity – features essential to its catalytic function. This work demonstrates the potential of upcycled MIL-53(Al) as a stable, sustainable, and low-cost heterogeneous catalyst for the synthesis of valuable acylhydrazides, offering an attractive alternative to conventional systems that rely on toxic metal promoters.

## Abstract 2

This study reports one of the few examples of acylhydrazide synthesis catalyzed by the metal–organic framework (MOF) MIL-53(Al) *via* the aldehyde C(sp^2^)–H hydrazidation reaction.

## Graphical abstract

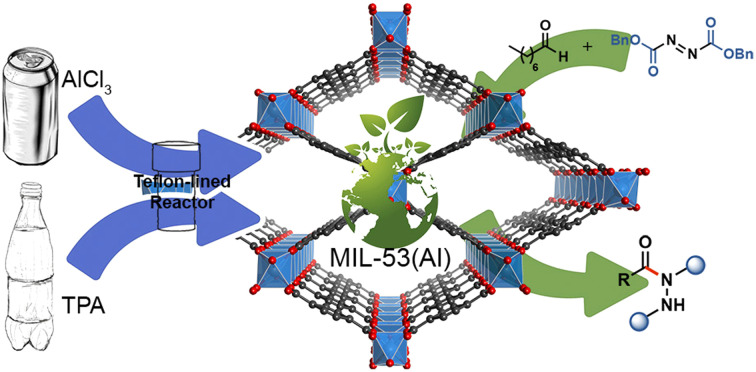

## Introduction

Contemporary society acknowledges the urgent need for recycling and the implementation of sustainable practices to mitigate environmental impact.^1^ The rapid industrial and population growth has led to a significant rise in plastic^2^ and metal waste,^3^ culminating in a pressing global environmental issue that impacts both the environment and the economy.^4,5^ Effective waste management strategies are crucial in this context, and innovative solutions such as upcycling offer promising approaches.^6^

**Fig. 1:**
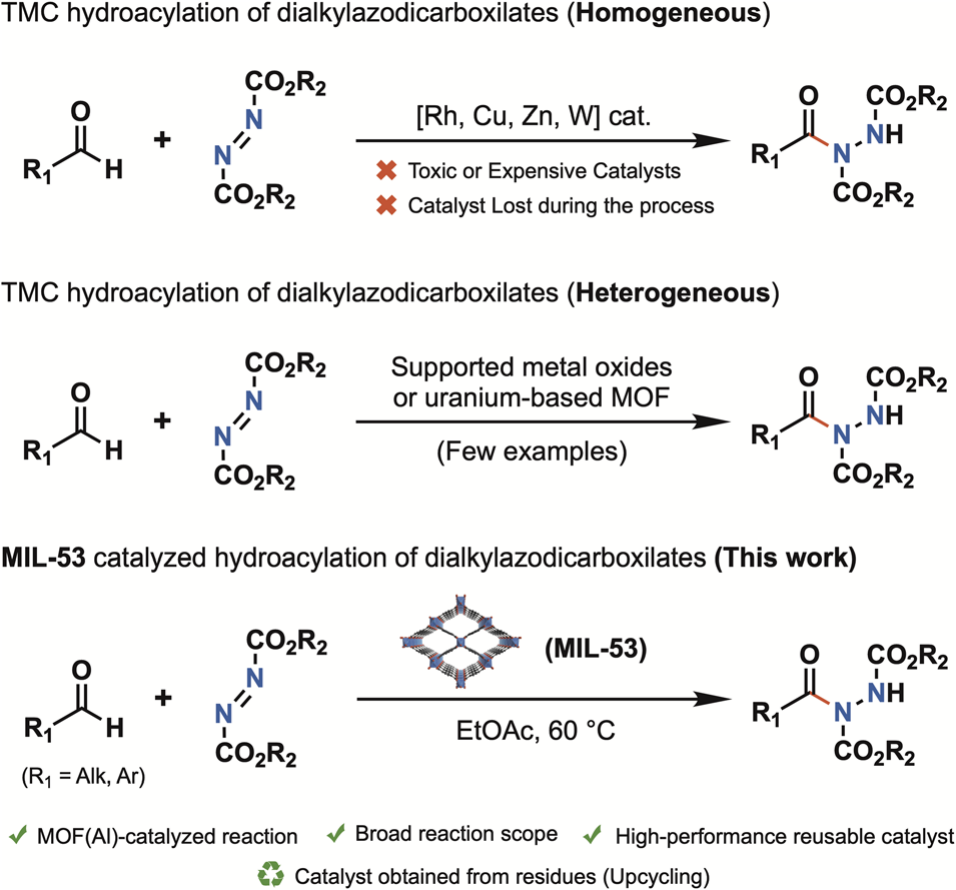
Precedents of transition-metal catalysed hydroacylation of dialkylazodicarboxylates and this work.

Acyl hydrazides are valuable intermediates for accessing pharmaceuticals,^7^ such as Vorinostat^8^ and Moclobemide,^9^ as well as agrochemicals^10^ and assorted natural products.^11^ In this regard, the hydroacylation of azodicarboxylate compounds with aldehydes has garnered significant attention for assembling these motifs with high efficiency under mild conditions.^12,13^ The reactions have been extensively studied using transition metals^14–17^ operating under homogeneous conditions (Fig. 1). Despite their high efficiency, the employment of these expensive and/or often toxic promoters, which are commonly lost during the process, significantly limits the practical synthetic utility of these transformations.

The most significant contributions are based on supported metal oxides, such as the CuO-np/SiO_2_ system reported by Mandal,^18^ or the work published by Ramon and co-workers exploring the IrO_2_·Fe_3_O_4_ and CoO·Fe_3_O_4_ catalytic systems.^19^ Nevertheless, to date, the application of Metal–Organic Frameworks (MOFs) in this chemical transformation remains largely unexplored; the only example reported to date is the use of a 2D uranium coordination polymer as a catalyst.^20^ On this context, MIL-53(Al) is noteworthy for its unique structural and functional properties. Its flexible framework offers exceptional porosity, high surface area, adjustable pore size, and stability under diverse conditions.^21^ These characteristics render MIL-53(Al) an ideal material for various applications, including gas storage,^22,23^ separation,^24,25^ drug delivery,^26^ and catalysis.^27–29^ Thus, MIL-53(Al) has emerged as a versatile heterogeneous catalyst capable of mediating various chemical reactions, including selective oxidations (*e.g.*, methanol oxidation to methyl formate),^30^ pyrrole synthesis,^31^ and Friedel–Crafts alkylation and acylation.^32,33^ Importantly, MIL-53(Al) is simple to synthesize, highly robust, and readily scalable, and it can even be prepared from recycled materials such as aluminium cans and PET bottles, offering exciting prospects for sustainable and circular chemistry.

The work described herein constitutes the first example of an aluminium-based MOF (MIL-53) catalyzed high-yield hydroacylation of dialkylazodicarboxylates, affording acylhydrazides bearing a new C_sp^2^_–N bond. The protocol displays broad substrate tolerance, accommodating alkyl, alkenyl, aryl, and heteroaryl aldehydes under mild conditions. Importantly, AlCl_3_ salts alone catalyse the transformation inefficiently, underscoring the critical role of the MOF architecture in enabling catalytic activity. Control experiments suggest that under reaction conditions, the material can promote the formation of reactive acyl radicals from aldehydes, which then evolve *via* a known radical mechanism. Moreover, recyclability studies confirm the retention of catalytic performance over multiple cycles, attesting to the robustness of the MIL-53(Al) catalyst. Notably, the synthesis of MIL-53(Al) was achieved using aluminium chloride (AlCl_3_) sourced from aluminium cans *via* acidic digestion, and terephthalic acid (TPA) recovered from PET bottles through alkaline hydrolysis. This synthetic approach not only demonstrates the catalyst's sustainability but also aligns with circular economy principles, reinforcing its potential for environmentally responsible catalysis.

## Results and discussion

### Synthesis and characterization of MIL-53(Al)

MIL-53(Al) was prepared by adapting the solvothermal method previously reported in the literature^34^ (SI, Scheme S2). AlCl_3_ (obtained upon acidic treatment of aluminium cans), 0.53 g (4 mmol), and TPA (yielded upon alkaline hydrolysis of PET bottles), 0.66 g (4 mmol), were dissolved in 10 mL of *N*,*N*-dimethylformamide (DMF) until reaching complete homogeneity. The solution was then transferred to a Teflon-lined stainless-steel autoclave and heated at 220 °C for 72 h. Afterwards, the autoclave was allowed to cool to room temperature to form crystals. Then, the crystals were washed with DMF and methanol, dried overnight at 100 °C, and activated by heating under vacuum at 150 °C (see SI for further details).

The crystalline structure and purity of the synthesized MIL-53(Al) were confirmed through various characterization techniques. Powder X-ray diffraction (PXRD) analysis showed sharp peaks between 9 and 40° 2*θ*, indicating high crystallinity and a well-formed MIL-53(Al) compound, consistent with literature values (SI, Fig. S3). FTIR spectra specify the material's functional groups (Fig. S4). The absorption band at 3400 cm^−1^ corresponds to the stretching vibration of the hydroxyl group in H_2_O. MIL-53(Al) exhibited vibrational bands around 1700–1400 cm^−1^, attributed to carboxylate groups. The coordination of carboxylate groups with Al^3+^ was confirmed by absorption bands at 1608 cm^−1^, 1510 cm^−1^ (asymmetric stretching), and 1420 cm^−1^ (symmetric stretching). It is worth noting that the absence of a band near 1700 cm^−1^ indicates no free terephthalic acid in the structure. Vibration bands between 730–1100 cm^−1^ are characteristic of C–H bending modes and, therefore, can be attributed to the presence of aromatic rings. Additionally, the absorption bands at 580 cm^−1^ and 470 cm^−1^ correspond to the stretching of the Al–O bond (SI, Fig. S6).^33,35,36^ On the other hand, the surface area and porosity were analysed by N_2_ adsorption–desorption experiments.

The prepared material exhibited type I isotherms with no hysteresis,^37^ a substantial BET surface area of 1040 m^2^ g^−1^, a pore size of 2.1 nm, and a pore volume of 0.45 cm^3^ g^−1^, indicative of a highly developed porous framework (SI, Fig. S5). Thermogravimetric analysis (TGA) showed a minor 4% mass loss at 230 °C, attributed to the removal of DMF. A mass loss of 66% occurred at 500–600 °C due to the decomposition of terephthalic acid, thus indicating a high thermal stability. Lastly, at 800 °C, the Al_2_O_3_ residue (29.7%) remained (SI, Fig. S6). Scanning Electron Microscopy (SEM) image showed a clustered assembly of uniform, rod-like particles, characteristic of MIL-53(Al) (SI, Fig. S7). The results are in complete agreement with those reported in the literature.^25,38,39^

### Hydrazidation of aldehydes with MIL-53(Al) as catalyst

Following the characterization of MIL-53(Al), we turned our attention to evaluating its catalytic efficacy in the hydroacylation of azodicarboxylate compounds with aldehydes, selecting octanal and dibenzyl azodicarboxylate as model reaction partners.

Initially, we investigated various parameters, including solvent, temperature, and reaction times, under different conditions (Table 1). Firstly, we evaluated the reaction in the absence of a catalyst at room temperature, which resulted in negligible formation of the desired product (Table 1, entries 1–4). Upon addition of the catalyst, the product was obtained in 73% and 69% isolated yields using propylene carbonate (PC) and dimethyl carbonate (DMC) as solvents, respectively, albeit with extended reaction times (Table 1, entries 5 and 6). The reaction rate improved markedly when dichloromethane (DCM) or ethyl acetate (EtOAc) was employed, affording 93% and 94% isolated yields after 28 hours (Table 1, entries 7 and 8). The heating of DCM to reflux slightly reduced the reaction time, while maintaining the high yield (Table 1, entry 9). Notably, deploying EtOAc at 60 °C delivered the product in an excellent 97% yield while avoiding the use of halogenated solvents (Table 1, entry 10). Under the optimized reaction conditions, polar protic solvents were also examined (Table 1, entries 11–13). In all cases, the reaction yields were noticeably lower than those obtained with the previously evaluated solvents, indicating that polar protic media do not contribute to any improvement in the reaction efficiency.

**Table 1:** Optimization for the hydrazidation of aldehydes reaction^a^

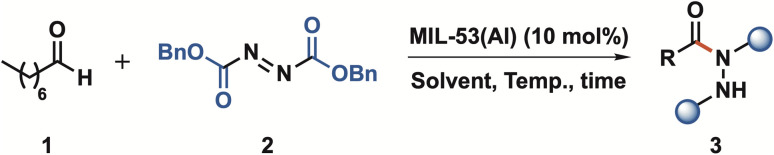					
Entry	MIL-53(Al) (mol%)	Solvent	Temp. (°C)	Time (h)	Yield^b^ (%)
1	0	DCM	RT	120	<5
2	0	EtOAc	RT	120	<5
3	0	DMC	RT	120	<5
4	0	PC	RT	120	<5
5	10	PC	RT	120	73
6	10	DMC	RT	120	69
7	10	DCM	RT	28	93
8	10	EtOAc	RT	24	94
9	10	DCM	40	20	94
10	10	EtOAc	60	10	97
11	10	EtOH	60	10	22
12	10	H_2_O	60	10	45
13	10	THF : H_2_O^c^	60	10	40

a Reaction conditions: octanal (300 µmol); dibenzyl azodicarboxylate (200 µmol) in the presence of the catalyst MIL-53(Al) (10 mol%) under solvents (200 µL) at different temperatures and reaction times in the absence of light.

b Isolated by silica gel column chromatography.

c A mixture of THF : H_2_O (1 : 1) was employed.

**Scheme 1:**
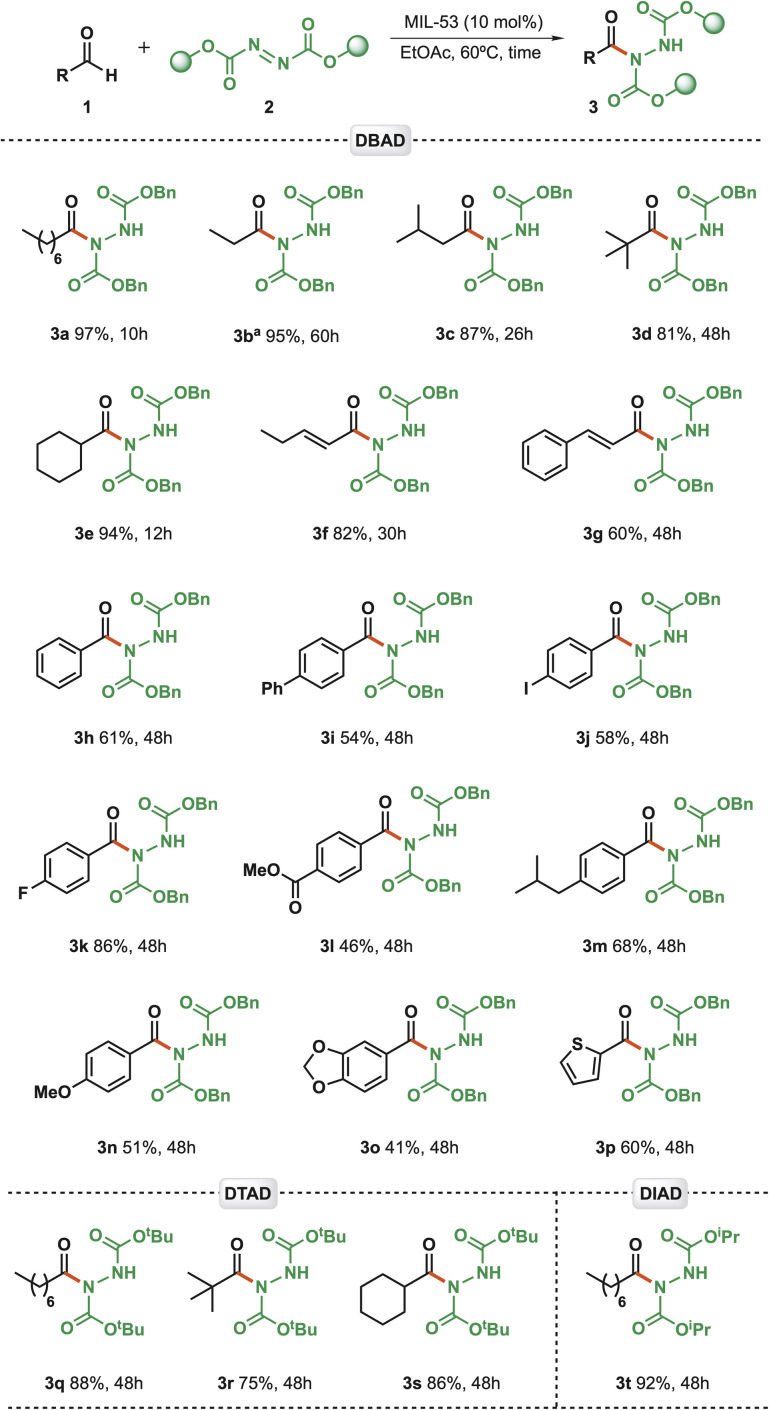
Scope of aldehyde C(sp^2^)–H hydrazidation. Reaction conditions: aldehyde (300 µmol); azodicarboxylate (200 µmol) in the presence of the catalyst MIL-53(Al) (10 mol%) in AcOEt (200 µL), with different reaction times at 60 °C. ^a^Carried out at 40 °C.

With the optimal conditions in hand, the scope of our C(sp^2^)–H hydrazidation strategy was explored by evaluating aldehydes and azodicarboxylates (Scheme 1). First, propanal (3b) exhibited an excellent yield (95%); however, it was over an extended reaction time of 60 h. Branched aliphatic aldehydes were also shown to be competent reaction partners, affording, therefore, compounds (3c) from isovaleraldehyde and (3d) from pivaldehyde in 87% and 81% yield, respectively. The slightly lower yields for these compounds can be attributed to steric hindrance and shorter reaction times.

A cyclic aldehyde (cyclohexanecarbaldehyde) was employed, affording compound (3e) in an excellent yield (94%) over 12 h. Moreover, the protocol was found to be successfully tolerant of α,β-unsaturated aldehydes (3f and 3g). Seeking to broaden the chemical space of our strategy, we further evaluated aromatic aldehydes over 48 hours. In these instances, lower yields were observed compared with aliphatic aldehydes, ranging from 54% to 86%. Benzaldehyde smoothly underwent C(sp^2^)–H hydrazidation in 61% yield (3h). Aldehyde substrates containing electron-withdrawing *para*-substituents (–I and –F) groups yielded from moderate to good conversion yields, as seen for compounds 3j (58%) and 3k (86%).

On the other hand, ester moieties (3l) did not exhibit the same behaviour, thereby significantly reducing the yield (46%). Switching to electron-donating substituents, we observed that methoxy (3n, 51%), biphenyl aldehyde (3i, 54%), and catechol acetal (3o, 41%) gave poorer results. In comparison, the aromatic aldehyde pendant with a *para*-isobutyl group gave a higher yield (3m, 68%). Gratifyingly, heteroaromatic aldehyde derived from thiophene was also accommodated, providing compound 3p in a good 60% yield. On the other hand, azodicarboxylate bearing the *tert*-butyl ester moiety gave the lowest conversion (compounds 3q, 3r, and 3s) over 48 hrs. Finally, the only example with an isopropyl moiety, 3t, exhibited a slightly decreased yield (92%).

To gain insight into the nature of the transformation, a series of control experiments was conducted. Under the optimized conditions, in the absence of the MIL-53(Al), the reaction afforded the product in a modest 37% yield (Table 2, entry 2). The use of AlCl_3_ at an equivalent molar loading gave a similarly low yield (Table 2, entry 3), indicating that free Al^3+^ ions do not seem to be involved in the observed activity. Terephthalic acid, the organic linker of MIL-53(Al), was also tested as a potential Brønsted acid catalyst, and the reaction yield was the same as that of the uncatalyzed reaction (Table 2, entry 4). Additionally, other common Brønsted and Lewis acids, comprising Amberlyst 15, Amberlite IR-120, β-zeolite, and mordenite zeolite, produced similar results (Table S2, entries 3–6). NH_3_-TPD experiment (SI, Fig. S8) revealed that the acidic properties of MIL-53(Al) are much more analogous to zeolites than to amberlite resins, featuring a similar distribution of acid strengths, comparable total acidity, and high thermal stability. The primary disadvantage highlighted in these results was the inability to characterize the type of acid sites (Brønsted *vs.* Lewis). Importantly, these findings suggest that the high activity of MIL-53(Al) arises from the cooperative interplay between its Al^3+^ Lewis acidic nodes and its porous, crystalline framework, rather than from simple homogeneous acid catalysis.

**Table 2:** Control experiments^a^

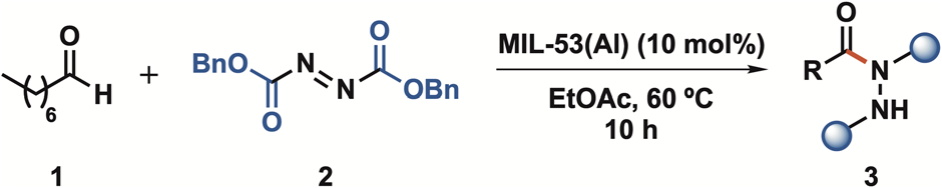		
Entry	Deviation from optimized conditions	Yield (%)
1	—	97
2	No catalyst	37
3	10 mol% of AlCl_3_ instead of MIL-53(Al)	40
4	Terephthalic acid	35
5	MIL-53/N_2_ atmosphere	36
6	Addition of 2 equiv. of TEMPO	Trace

a Reaction conditions: octanal (300 µmol); dibenzyl azodicarboxylate (200 µmol) in the presence of the catalyst MIL-53(Al) (10 mol%) in ethyl acetate (200 µL) at 60 °C for 10 hours under air atmosphere.

**Scheme 2:**
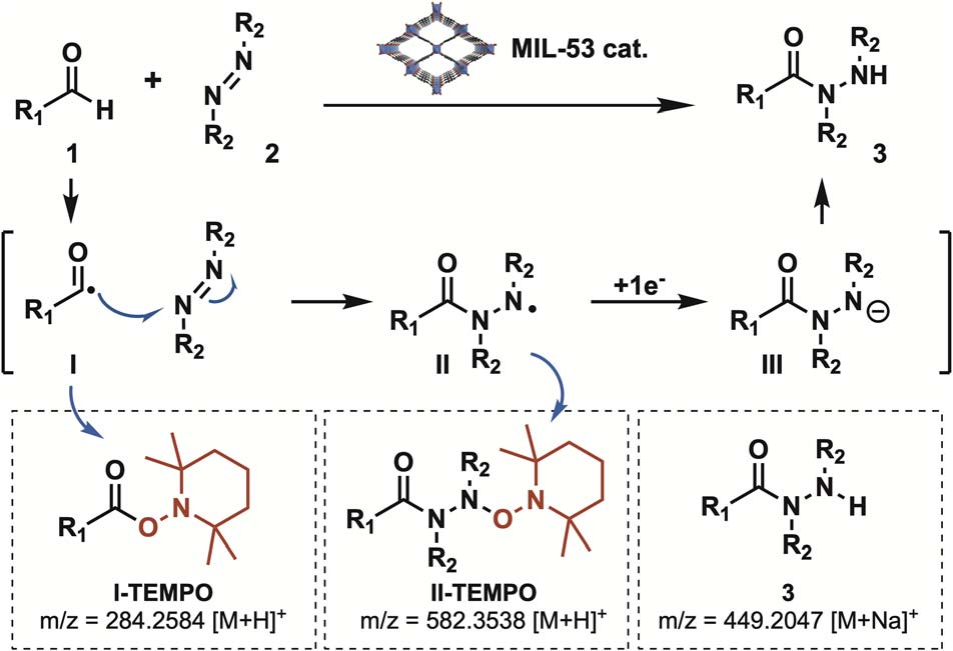
Mechanistic proposal.

Interestingly, the reaction performed under inert atmosphere (N_2_) showed an important decrease in the reaction yield, suggesting the participation of aerobic oxidation within the process (Table 2, entry 5).^40^ Furthermore, radical-trapping experiments were performed (Table 2, entry 6). The addition of 2.0 equivalents of TEMPO under optimized conditions resulted in negligible product formation, suggesting the involvement of radical intermediates. High-resolution mass spectrometry (HRMS) analysis of the crude mixture (see SI) confirmed the presence of TEMPO adducts corresponding to key radical species proposed in the canonical mechanism (Scheme 2).^12^ Specifically, the acyl radical I, generated from aldehyde 1, was detected as the TEMPO adduct at *m/z* = 284.2584 [M + H]^+^. This radical underwent addition to the N=N bond of diazocarboxylate 2, forming the N-centered radical II, which was likewise identified as its TEMPO adduct (*m/z* = 585.3538 [M + H]^+^). Subsequent single-electron reduction of II (reductive radical-polar crossover) followed by protonation, affording the desired product 3.

### Recycling and structure stability of MIL-53(Al)

**Fig. 2:**
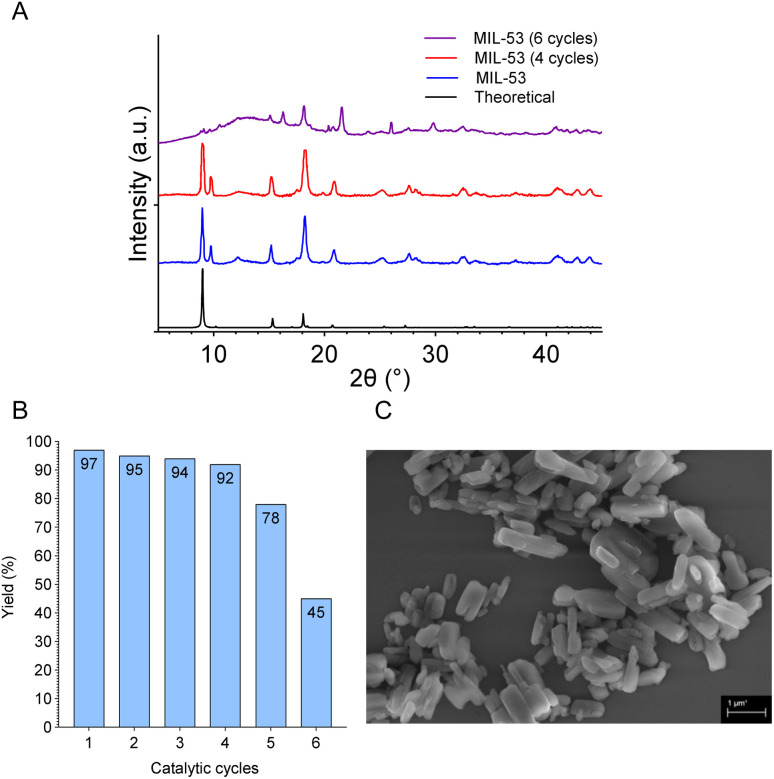
Characterization of MIL-53(Al) after reuse. (A) PXRD, (B) evaluation of the recyclability, and (C) SEM image.

Next, by focusing on its performance across multiple cycles, the recycling potential of the heterogeneous MIL-53(Al) catalyst was explored (Fig. 2). Seminal findings indicated that the catalyst could be efficiently recovered and redeployed after a simple filtration process. Notably, a reduction in catalytic efficiency (5%) following the fourth cycle can be considered negligible. This observation underscores the robustness of MIL-53(Al), highlighting its capacity for repeated application with minimal loss in activity.

Furthermore, post-reuse PXRD analysis (Fig. 2A) confirms that the MOF's structural composition and crystalline nature remain unchanged through multiple cycles, thereby demonstrating its durability and sustainability as a catalyst in chemical synthesis (Fig. 2B). SEM analysis conducted after the fourth catalytic cycle reveals that MIL-53(Al) maintains its distinct rod-like morphology (Fig. 2C). The image displays the characteristic particle assembly, with only slight changes in texture and edge definition, potentially attributable to the accumulation of reaction by-products or minor structural rearrangements during catalysis. Nonetheless, the overall particle size and shape distribution remain consistent with those of the pristine catalyst, indicating that the physical integrity of MIL-53(Al) is preserved mainly after repeated use.

The catalytic performance profile indicates a progressive loss of activity over successive cycles. The slight decrease in yield from 97% to 92% between cycles 1 and 4 is typical and may be attributed to a minor loss of active sites or initial pore blocking. The further decline to 78% in cycle 5 suggests a more severe deactivation event, possibly due to partial structural collapse or increased obstruction of the porous network, which reduces access to active sites. Catalyst poisoning caused by the accumulation of by-products or impurities within the pores may also contribute, reaching a critical point where a significant fraction of active sites becomes inaccessible. By cycle 6, catalytic failure is evident, with the yield dropping sharply to 45%, indicating that the catalyst has lost most of its activity. At this stage, the MOF framework is likely sufficiently degraded to render it ineffective, supporting the conclusion that reuse beyond the 4^th^ or 5^th^ cycle is not viable.

Finally, to assess the possibility of Al^3+^ leaching, both the reaction product 3a solution and the digested MIL-53(Al) samples were analyzed. 3a solution samples were subjected to microwave-assisted acid digestion, while the MOF was fully digested under the same conditions to quantify its total Al content. In the case of product 3a solution, no aluminum was detected (ND) across triplicate measurements, with mean absorbance values close to baseline (0.0001–0.0009). These results indicate that the product is free of Al^3+^ contamination from the catalyst and did not leach into the organic phase (see SI).

## Conclusions

In summary, we have demonstrated the first example of an aluminium-based MOF, MIL-53(Al), catalyzing the hydroacylation of aldehydes with azodicarboxylates for the efficient synthesis of acylhydrazides. The method exhibits a broad substrate scope, enabling the functionalization of alkyl, alkenyl, aryl, and heteroaryl aldehydes in high yields. The catalyst maintains its crystallinity, porosity, and activity for at least 4 consecutive cycles, demonstrating its robustness and recyclability. Control experiments indicate that the cooperative environment of the MOF framework, rather than simple Brønsted or Lewis acidity, is essential for the observed activity. Importantly, MIL-53(Al) was synthesized entirely from aluminium cans and PET bottles, valorising waste into a functional catalytic material. This work underscores the potential of upcycled MOFs as cost-effective, stable, and environmentally benign alternatives to conventional metal-based catalytic systems, advancing the principles of sustainable catalysis.

## Author contributions

RBG, investigation, methodology, validation, formal analysis, writing – original draft. LM, JACD, and FV, investigation, validation, and writing – review & editing. MWP and CAJ, conceptualization, visualization, resources, writing – review & editing, and funding acquisition. ECP, KCA, and SSM, investigation and validation. RSV, visualization, and writing – review & editing.

## Conflicts of interest

There are no conflicts to declare.

## Supplementary Material

RA-015-D5RA07909H-s001

## Data Availability

The data supporting this article have been included as part of the supplementary information (SI). Supplementary information is available. See DOI: https://doi.org/10.1039/d5ra07909h.
