# Supplementary material for: MOF MIL-53(Al) catalyzed hydroacylation of azodicarboxylates: sustainable catalyst for heterogeneous acylhydrazide synthesis
Source: RSC Adv. 2025 Dec 10;15(57):49159–64. doi: 10.1039/d5ra07909h (PMC12691338; doi:10.1039/d5ra07909h)
Supplement: RA-015-D5RA07909H-s001 [file RA-015-D5RA07909H-s001.pdf]

Supplementary Information (SI) for RSC Advances.
This journal is © The Royal Society of Chemistry 2025

Supporting Information

MOF MIL-53(Al) Catalyzed Hydroacylation of Azodicarboxylates: Sustainable Catalysts for Heterogeneous Acylhydrazide Synthesis

Reynier Báez,[a] Lucas Marchini,[b] José A. C. Delgado,[b] Sebastián San Martín,[a] Felipe Verdugo,[a] Kelvin C. Araújo,[c] Ernesto C. Pereira,[c] Rajender S. Varma,[b] Marcio W. Paixão*[b] and Claudio A. Jiménez*[a]

[a] Department of Organic Chemistry, Faculty of Chemical Sciences, Universidad de Concepción, Concepción 4130000, Chile. E-mail: cjimenez@udec.cl

[b] Laboratory for Sustainable Organic Synthesis and Catalysis, Department of Chemistry, Federal University of São Carlos, Rodovia Washington Luís, km 235 -SP310, São Carlos, São Paulo, 13565-905, Brazil. mwpaixao@ufscar.br

[c] CDMF, LIEC, Department of Chemistry, Federal University of São Carlos, Rodovia Washington Luís, km 235 -SP310, São Carlos, São Paulo, 13565-905, Brazil.

Table of Contents:

1) General Information 2

2) Characterization methods 2

3) Experimental Procedure 5

3.1) Upcycling of the Metal-Organic Framework MIL-53(Al) 5

3.1.1) General Procedure (A): Depolymerization of Poly(ethylene terephthalate) (PET) for the Recovery of Terephthalic Acid (TPA). 5

3.1.2) General Procedure (B): Chemical Conversion of Aluminum Cans into Aluminum Chloride ($AlCl_3$) 6

3.1.3) General Procedure (C): Solvothermal Synthesis of MIL-53(Al) 6

3.2) General Procedure for the Synthesis of Acylhydrazides 7

3.3) General Procedure for the reuse of the MOF MIL-53(Al) 7

4) Characterization Section 8

4.1.1) Terephthalic Acid 8

4.1.2) Aluminum chloride 10

4.1.3) MIL-53(Al) 10

4.1.4) MIL-53(Al) after reuse 20

5) NMR Spectroscopic Data of Compounds 22

6) Characterization Spectra of Compounds **3a** - **3t**. 29

1) General Information

All solvents were dried and distilled before use by standard procedures, and reagents were of the highest commercially available grade, purchased from suppliers including Sigma-Aldrich, Alfa-Aesar, TCI, ABCR, Acros, Fluorochem, and Merck Millipore, and used as received or purified according to the procedures outlined in Purification of Common Laboratory Chemicals.[1] Glassware used was dried in an oven or flame dried under a vacuum and cooled under an inert atmosphere. Column flash chromatography was performed using silica gel 60 M (40-63 µm, 230-440 mesh, Merck), and analytical thin-layer chromatography (TLC) was performed using gel-coated alumina plates (MN TLC sheets ALUGRAM ® Xtra SIL G /UV254). Compounds were visualized on TLC by UV-light (254 nm), $KMnO_4$, I_2, $H_3[P(Mo_3O_{10})_4]xH_2O$ (PMA) and Vanillin. Yields refer to chromatographically and spectroscopically pure compounds unless otherwise noted.

2) Characterization methods

Powder X-ray Diffraction (PXRD): PXRD analysis was performed using a Rigaku-3070-E diffractometer equipped with a graphite monochromator and Cu Kα radiation (λ = 1.5418 Å). The samples were ground into fine powders and placed on circular sample holders with an internal diameter of 1 cm. The crystallinity of the synthesized materials was examined by collecting data over a 2θ angular range of 2–80°, with a step size of 0.02° and a measurement time of 30 minutes per scan.

FTIR Spectroscopy: FTIR spectra were recorded using a Nicolet FTIR Magna 550 spectrometer (Thermo Scientific, USA) with dry KBr pellets. Measurements were performed in the mid-infrared region (4000 – 400 cm^{-1}), and wavenumber ($\tilde{v}$) values are reported in cm^{-1}.

Thermogravimetric analysis: (TGA) was carried out using a TGA Q500 instrument (TA Instruments). The thermal decomposition profile of the material was monitored by recording the mass loss as the temperature was ramped from 30 to 600°C at a constant heating rate of 10°C·min^{-1}.

N_2 Adsorption/Desorption Isotherms: N_2 adsorption/desorption isotherms were measured using a Micromeritics TriStar II 3020 analyzer with a liquid nitrogen bath at -196 °C. The sample was pretreated under vacuum at 120 °C for 3 hours. The Specific surface area (S_{BET}) and pore volume (V_p) values were determined using the Brunauer-Emmett-Teller (BET)

method. The average pore diameter (D_p) was calculated using the Barrett-Joyner-Halenda (BJH) method with the TriStar II 3020 interface software.

Scanning Electron Microscopy (SEM): SEM analysis was performed using a Supra 35-VP microscope (Carl Zeiss, Oberkochen, Germany) operated at 5 kV.

Ammonia Temperature-Programmed Desorption (NH_3-TPD): The NH_3-TPD measurements were carried out using a Micromeritics Autochem II 2920 Chemisorption Analyzer with a thermal conductivity detector. For that, 100 mg of the powder samples were thermally treated under a helium flow at a heating rate of 10 °C.min^{-1} from room temperature to 350 °C and kept at this temperature for 45 min. After that, the reactor was cooled to 120 °C and the helium flow was kept for 60 min. Next, the adsorption of ammonia was conducted with a 10% (V/V) mixture of NH_3 in He for 30 min over the sample and after the saturation, the sample was purged with He flow for 1 h to remove the excess of ammonia. The desorption measurements were performed under a of He flow and a heating rate of 15 °C.min^{-1} from 120°C to 500 °C.

NMR spectroscopy: All NMR spectra were recorded at room temperature using a Bruker Avance 400 (400 MHz for ^{1}H, 100 MHz for ^{13}C) NMR spectrometer. Chemical shifts (δ) are reported in parts per million (ppm) relative to the residual solvent signals, tetramethylsilane (TMS), deuterated chloroform ($CDCl_3$), or deuterated dimethyl sulfoxide (DMSO-d_6). Coupling constants (J) are given in hertz (Hz). The following abbreviations indicate signal multiplicities in the ^{1}H-NMR spectra: br = broad, s = singlet, d = doublet, t = triplet, q = quartet, dd = doublet of doublets, dt = doublet of triplets, and m = multiplet.

UHPLC-QTOF-MS/MS: After sample preparation, analyses were performed using an ultra-high-performance liquid chromatograph (UHPLC) Agilent 1290 Infinity II (Agilent Technologies, Santa Clara, CA, USA). Chromatographic separation was carried out on a C18 column (100 mm × 2.1 mm i.d., 1.8 µm, Zorbax Eclipse Plus, Agilent, USA). The UHPLC mobile phase consisted of H_2O (solvent A) and ACN (solvent B), each containing 0.1% formic acid. The gradient elution program was as follows: 0-0.5 min, 5% B; 0.5-5 min, 5 to 90% B; 5-7 min, 90% B; 7-8 min, 90 to 5% B; and 8-10 min, 5% B. The column oven and autosampler temperatures were maintained at 25 ºC. The flow rate was 0.30 mL.min^{-1}, with an injection volume of 10 µL. Simultaneously, spectrometric analyses were conducted using an Agilent 6545 Q-TOF-MS/MS system (Agilent Technologies, Santa Clara, CA, USA) equipped with a

Jet Stream ESI interface operating in positive ion mode, with a capillary voltage (VCap) of 3.2 kV. The gas flow rate was set to 12 $L.min^{-1}$, with a gas temperature of 350 ºC, nebulizer pressure of 35 psi/g, nozzle voltage of 750 V, fragmentor voltage of 1125 V, and skimmer voltage of 45 V. Collision energies were evaluated in the range of 5–40 V. Molecular ions were acquired in MSE mode. Data were collected over the mass range of 50–1000 Da at a scan rate of 3.0 $spectra/s^{-1}$ and processed using MassHunter Workstation Software, Version B.08.00.

Chromatographic and Mass Spectrometric Analysis: Chromatographic analyses were performed using an ACQUITY UPC^2 supercritical fluid chromatograph (Waters Corporation) coupled to a Waters Xevo TQD mass spectrometer. Chromatography was carried out with a photodiode array (PDA) detector operating in the wavelength range of 210 – 400 nm, with a resolution of 1.2 nm and a sampling rate of 20 $points.s^{-1}$. The total run time was 7.00 minutes at a flow rate of 1 $mL.min^{-1}$, a column temperature of 35 °C, and an injection volume of 2 μL. The mobile phase consisted of CO_2 (A) and methanol (B), with the following gradient: 0.00 – 5.00 min: 75% A, 25% B; 5.00 – 6.00 min: 75% A, 25% B; 6.05 – 7.00 min: 100% A, 0% B. Mass spectrometry was performed using a capillary voltage of 2.50 kV in negative electrospray ionization mode (ES^-). The mass range was set from 150 to 650 Da, with a collision energy of 7 eV and a cone voltage of 30 V. The source temperature was 150 °C, and the desolvation temperature was 350 °C.

Atomic Absorption Spectroscopy (AAS): Aluminum quantification was performed using a GBC XplorAA atomic absorption spectrometer equipped with SavantAA software (Version 3.11a). The instrumental conditions for Al determination were as follows: wavelength, 309.30 nm; slit width, 0.5 nm; lamp current, 6 mA; and acetylene–nitrous oxide flame. A GBC aluminum hollow cathode lamp was employed, and a deuterium lamp was used for background correction. Measurements were carried out in integration mode with a read time of 1 s and an equivalent time constant. Aluminum concentrations were expressed relative to the mass of MOF. Aliquots of 50 mg of each sample were weighed in triplicate and placed into TFM digestion vessels of a microwave chemistry workstation (MASTER 16, Microwave Chemistry Workstation). To each vessel, 2 mL of concentrated HNO_3 and 2 mL of concentrated HCl were added inside a fume hood. The vessels were sealed and loaded into the microwave system according to the manufacturer's instructions. The temperature program was as follows: ramp to 100 °C in 10 min and hold for 10 min; ramp to 150 °C in 10 min and hold for 10 min; ramp to 200 °C in 15 min and hold for 30 min to ensure complete digestion.

After digestion, the vessels were cooled for at least 5 min before handling. The digests were transferred to acid-cleaned glass bottles, and 0.5 mL of 30% H_2O_2 was added to each. Samples were then quantitatively transferred to 100 mL acid-cleaned glass volumetric flasks and diluted to volume with ultrapure water (resistivity 18.2 MΩ·cm). To avoid nebulizer clogging during AAS analysis, all samples were filtered through Whatman 42 filter paper (Cytiva). All volumetric flasks and filtration apparatus were pre-cleaned with acid. Reagent blanks were prepared and processed in the same manner as the samples. Calibration standards were prepared from a 1000 ± µg/mL aluminum stock solution (Inorganic Ventures). All standards contained 2.0 % HCl, 2.0 % HNO_3, and 0.15 % H_2O_2 (v/v) in 100 mL.

3) Experimental Procedures

3.1) Upcycling of the Metal-Organic Framework MIL-53(Al)

3.1.1) General Procedure (A): Depolymerization of Poly(ethylene terephthalate) (PET) for the Recovery of Terephthalic Acid (TPA).

As shown in Scheme S1, the basic hydrolysis of PET was performed according to the following procedure.[3] A total of 4 g of PET, previously cut into small pieces, was placed in a beaker. Then, 50 mL of a 5 mol·L^{-1} NaOH solution was added, and the mixture was heated to a boil under constant stirring. After the reaction, the resulting white solid, corresponding to sodium terephthalate, was dried and subsequently resuspended in 100 mL of deionized water. Sulfuric acid (7 mL) was then added dropwise until complete precipitation occurred. The white solid terephthalic acid was collected by filtration, washed with water to remove any impurities, and obtained a 98 % yield (2.97 g). Finally, the product was dried in an oven for several hours and stored for further characterization and use.

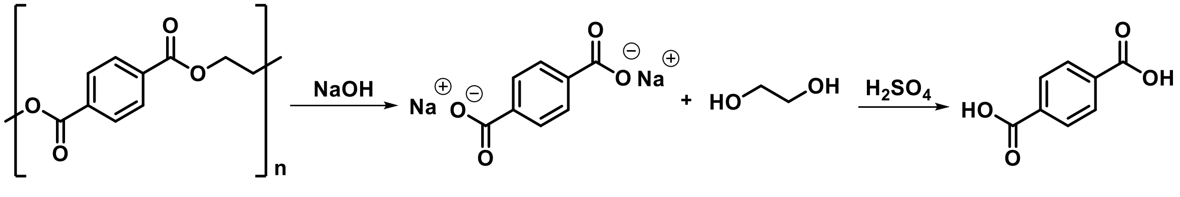

Scheme S1. Depolymerization of poly(ethylene terephthalate) to terephthalic acid.

3.1.2) General Procedure (B): Chemical Conversion of Aluminum Cans into Aluminum Chloride ($AlCl_3$)

An aluminum can was thoroughly washed with water to remove the external labels and residues. After drying, it was cut into small pieces, and 3.7 g of the material was placed in a beaker. Subsequently, 100 mL of deionized water and 50 mL of HCl (32%) were added. The mixture was stirred continuously at room temperature until the complete dissolution of metallic aluminum. The resulting aluminum chloride solution exhibited a final pH between 3 and 4. The solution was then evaporated until dryness, affording a light-yellow solid. A portion of the solid was ground in an agate mortar and submitted for powder X-ray diffraction analysis (PXRD).

3.1.3) General Procedure (C): Solvothermal Synthesis of MIL-53(Al)

MIL-53(Al) was synthesized via a solvothermal method based on previously reported procedures, with minor modifications.[4] $AlCl_3$, 0.53 g (4 mmol), and terephthalic acid, 0.66 g (4 mmol), were dissolved in 10 mL of *N*,*N*-dimethylformamide (DMF) to form a clear solution. The mixture was transferred to a Teflon-lined stainless-steel autoclave and heated at 220 °C for 72 h. After cooling to room temperature, the resulting solid was collected and washed with DMF (3 x 30 min), followed by methanol (3 x 24 h). The obtained MIL-53(Al) was dried overnight at 200 °C and subsequently activated under vacuum at 150 °C for 24 h (Scheme S2).

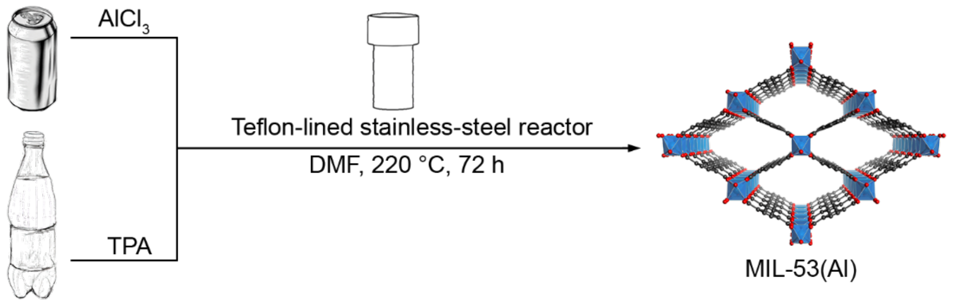

Scheme S2. Solvothermal synthesis of MIL-53(Al) from recycled precursors.

3.2) General Procedure for the Synthesis of Acylhydrazides

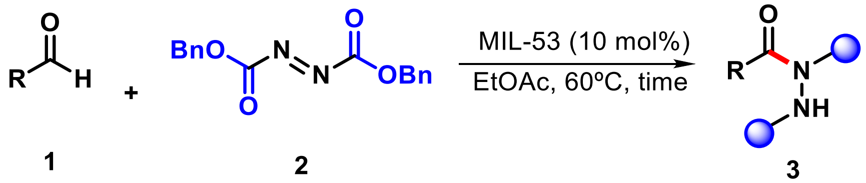

Scheme S3. General procedure for the synthesis of acylhydrazides

The reaction was carried out in a vial containing aldehyde (octanal) (300 μmol, 1.5 equivalents) and azo compound (dibenzyl (*E*)-diazene-1,2-dicarboxylate) (200 μmol, one equivalent) in the presence of MIL-53(Al) (10 mol%) in ethyl acetate (200 μL) at 60°C. The reaction was stirred until a color change was observed or for a maximum of 48 h. A variety of aliphatic and aromatic aldehydes, along with other azo compounds such as di-*tert*-butyl (*E*)-diazene-1,2-dicarboxylate and diisopropyl (*E)*-diazene-1,2-dicarboxylate, were employed to generate a diverse compound library. All reactions were monitored by thin-layer chromatography (TLC) using silica gel-coated plates. Reactions progress was monitored by thin-layer chromatography (TLC). Upon completion, the mixture was separated by centrifugation and purified by flash column chromatography using ethyl acetate in hexanes (7:3) as the eluent, affording the desired products.

3.3) General Procedure for the reuse of the MOF MIL-53(Al)

The reusability of MIL-53(Al) was evaluated over four consecutive catalytic cycles under the standard reaction conditions (octanal, 300 μmol; dibenzyl azodicarboxylate, 200 μmol; MIL-53(Al), 10 mol%; EtOAc, 200 μL; 60 °C; 10 h). After each cycle, the reaction mixture was cooled to room temperature, and the solid catalyst was recovered by centrifugation. The recovered MIL-53(Al) was washed sequentially with ethyl acetate (3 × 5 mL, 5 min each), methanol (2 × 5 mL, 10 min each), and acetone (5 mL, 5 min), then dried at 100 °C for 6 h. The dried material was activated under vacuum at 150 °C for 24 h, cooled to room temperature under vacuum, and weighed to confirm consistent catalyst loading. The regenerated MIL-53(Al) was then reused in the next cycle without further treatment.

4) Characterization Section

4.1.1) Terephthalic Acid

Figure S1 (**a** and **b**) displays the ^{1}H and ^{13}C NMR spectra of terephthalic acid, respectively. In the ^{1}H NMR spectrum, a single sharp signal is observed at 8.0 ppm, corresponding to the four equivalent aromatic protons of the benzene ring. Additionally, a signal at 13.3 ppm is attributed to the two carboxylic acid protons. The absence of signal in the aliphatic region confirms the fully aromatic nature of the compound and indicates the successful remove of residual ethylene glycol byproduct from the PET depolymerization process. The ^{13}C NMR spectrum shows three distinct signals, consistent with the expected carbon environments in terephthalic acid. The signal at 166.7 ppm corresponds to the carboxylic acid carbon atoms, while the signal at 129.5 ppm is assigned to the aromatic C-H carbons. The signal at 134.5 ppm is attributed to the quaternary carbon bonded directly to the carboxylic groups, reflecting the electron withdrawing nature of these substituents. The overall NMR data confirms the structure and high purity of the isolated terephthalic acid. The absence of additional proton or carbon signals further supports the efficient depolymerization of PET into the desired product without detectable impurities.

Figure S1(**c**) presents the FTIR spectrum of terephthalic acid, revealing characteristic absorption bands associated with the molecular vibrations of the functional groups present in the structure. A broad band in the region of 3200–3500 cm^{-1} is attributed to the O-H stretching vibrations of hydroxyl groups in terephthalic acid, indicating the presence of hydrogen bonding. The band in the 2900–3000 cm^{-1} region corresponds to the C-H stretching vibrations of aromatic rings. A strong band observed at 1680 cm^{-1} is assigned to the C=O stretching of the carboxylic acid group, confirming its presence. The band at 1419 cm^{-1} is attributed to in-plane bending of C-O-H moiety, while the band at 1285 cm^{-1} corresponds to C-O stretching vibrations. Overall, the spectral analysis confirms the presence of key functional groups consistent with the chemical structure of terephthalic acid.

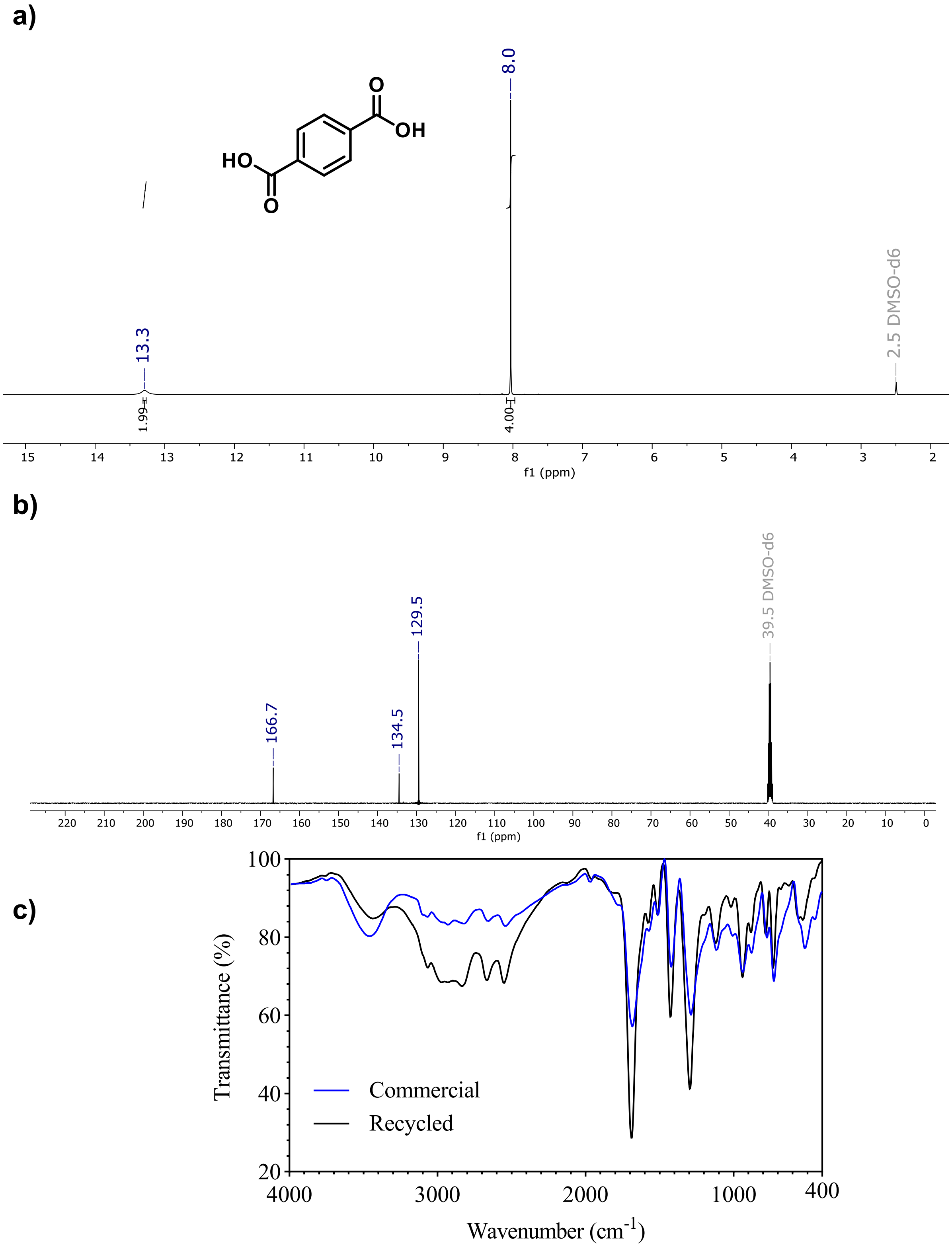

Figure S1. Characterization of Terephthalic Acid. a) ^{1}H NMR, b) ^{13}C NMR, and c) FTIR spectra

4.1.2) Aluminum chloride

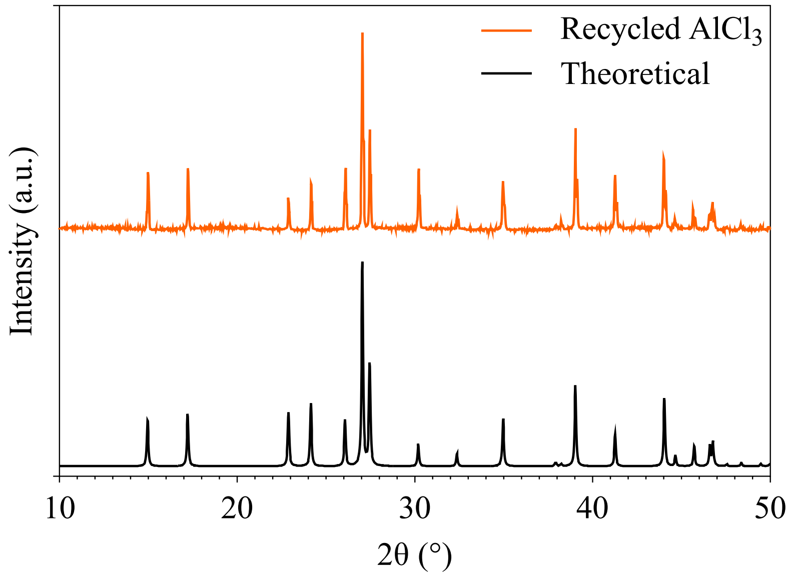

Figure S2. PXRD pattern of recycled Aluminum chloride.

Powder X-ray Diffraction (PXRD) analysis was employed to evaluate the crystalline structure of the aluminum chloride obtained from recycled aluminum cans. As shown in Figure S2, the PXRD pattern displays several distinct peaks characteristic of a crystalline material. These peaks were compared to the theoretical diffraction pattern of aluminum chloride. The major diffraction peaks observed for the recycled $AlCl_3$ closely match the principal peaks in the reference pattern, corresponding to known crystallographic planes of the compound. The agreement in 2θ positions confirm the successful formation of $AlCl_3$ from the aluminum can-derived precursor. Moreover, the sharpness of the diffraction peaks indicates a high degree of crystallinity in the recovered material.

4.1.3) MIL-53(Al)

PXRD analysis of the synthesized MIL-53(Al) is presented in Figure S3. The diffraction pattern exhibits sharp peaks in the 2θ range of 9° to 40°, characteristic of the crystalline structure of MIL-53(Al). These reflections confirm the successful formation of MIL-53(Al) crystals with high crystallinity. The observed pattern is consistent with previously reported PXRD data for pure MIL-53(Al), indicating that the material was obtained with structural fidelity to the expected framework.[5]

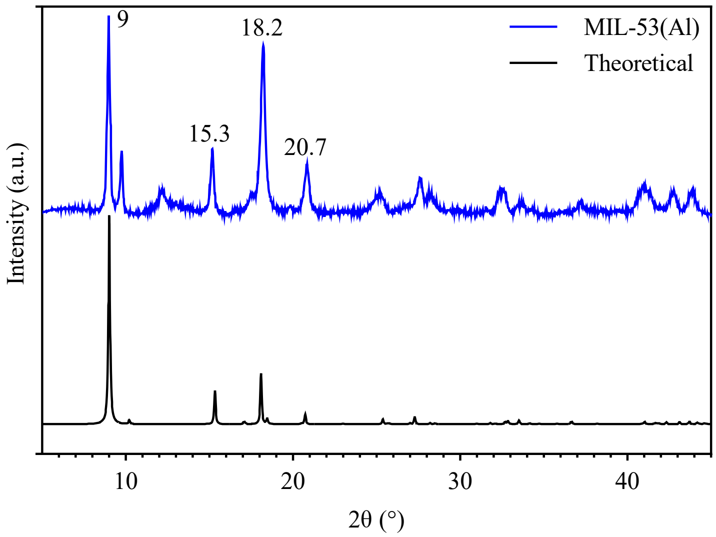

Figure S3. PXRD pattern of MIL-53(Al).

Figure S4 shows the FTIR spectrum of MIL-53(Al), which displays a series of intense and well-defined absorption bands corresponding to functional groups present in the MOF structure. The broad band centered around 3400 cm^{-1} is attributed to O-H stretching vibrations, indicating the presence of adsorbed water molecules within the porous framework. The strong and sharp bands observed at approximately 1608 cm^{-1} and 1420 cm^{-1} are assigned to the asymmetric and symmetric stretching vibrations of carboxylate groups (-COO), respectively, confirming the coordination of these groups to aluminum. Additionally, absorption bands in the 730 – 1100 cm^{-1} region, related to C-H bending vibrations, indicate the presence of aromatic rings within the organic linker. Finally, low-wavenumber bands between 470 and 580 cm^{-1} are attributed to Al-O stretching vibrations, which are characteristic of the MIL-53(Al).[5-6]

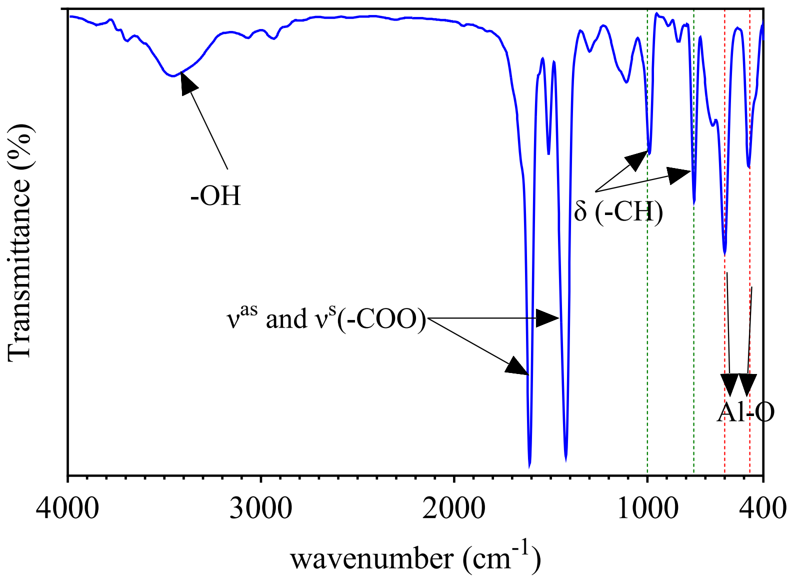

Figure S4. Infrared spectrum of activated MIL-53(Al) in dry KBr.

The surface area, pore volume, and pore size distribution of MIL-53(Al) were determined using the BET and BJH methods, as shown in Figure S5. The nitrogen adsorption-desorption isotherm exhibits a typical type I profile, according to the IUPAC classification,[7] which is typical of microporous materials or materials with narrow pores. BET analysis revealed a specific surface area of 1040 m^2/g, with a total pore volume of 0.44 cm^3/g. The pore size distribution is centered around 2.1 nm, with a narrow range, indicating the formation of porous materials with well-defined and relatively uniform channels. These results are consistent with data reported in previous literature. [5-6]

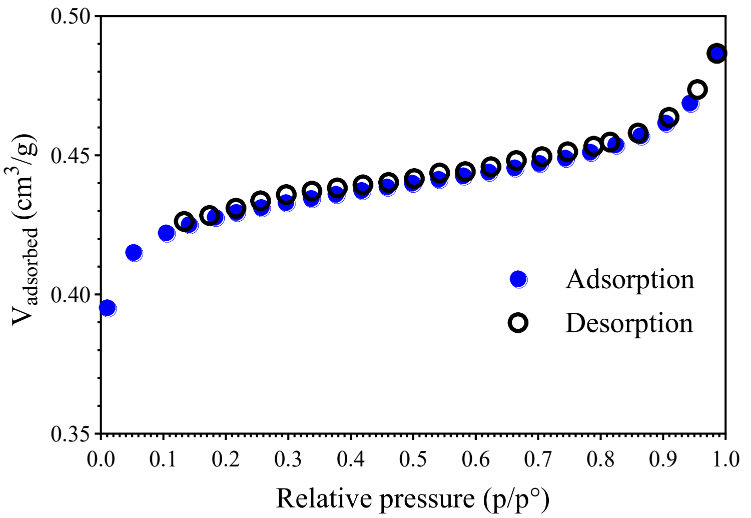

Figure S5. N_2 isotherm at 77 K of MIL-53(Al).

Figure S6 presents the Thermogravimetric analysis (TGA) of MIL-53 (Al). The profile shows a minor weight loss of approximately 4% at 230 °C, attributed to the release of residual DMF solvent confined within the MOF structure. A more pronounced weight loss, about 66.1%, occurs between 500 and 600 °C, corresponding to the decomposition of terephthalic acid as organic linker. At 800 °C, the remaining solid residue corresponds to amorphous Al_2O_3 representing 29,7% of the initial mass. These results demonstrate the high thermal stability of MIL-53 (Al) [8]

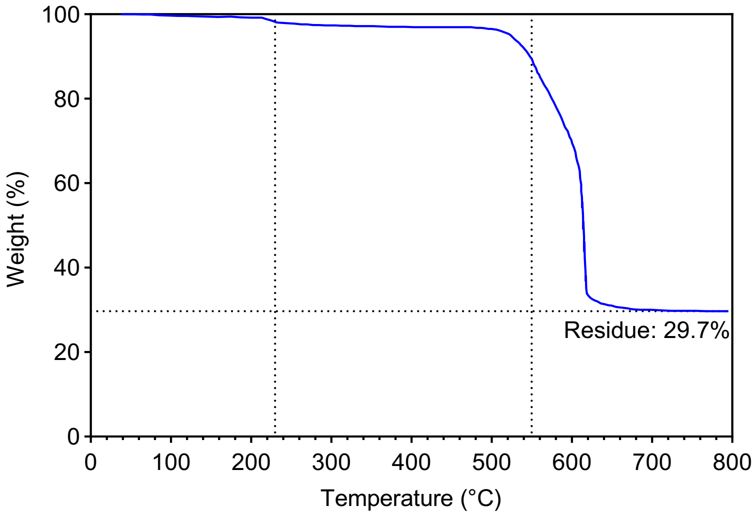

Figure S6. Thermogravimetric analysis of MIL-53(Al)

Figure S7 presents the Scanning Electron Microscopy (SEM) images of MIL-53 (Al) acquired at two magnifications, providing an overview of the material's morphology. The material exhibits a clustered arrangement of rod-like particles, which is characteristic of the MIL-53(Al) framework. The particles display a relatively uniform morphology, suggesting a well-controlled synthesis process. [8]

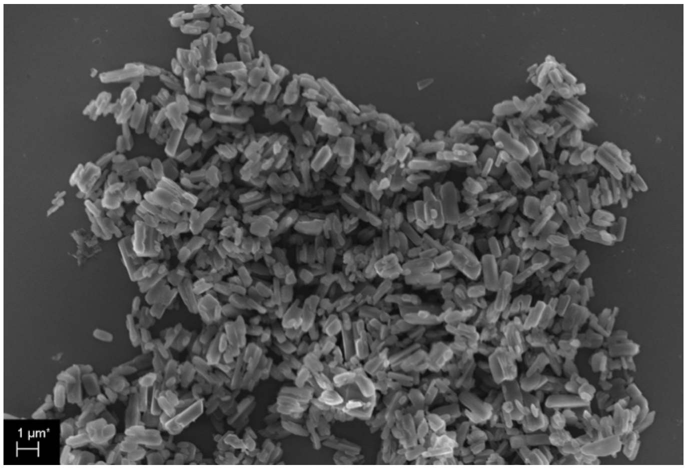

Figure S7. SEM images of MIL-53(Al)

From the NH_3-TPD profile of MIL-53(Al) (Figure S9), a broad desorption band centered at approximately 210 °C is observed, indicating the presence of weak to moderate acid sites in the material. In addition, a distinct high-temperature desorption event appears at around 460 °C, which can be associated with the presence of strong acid sites.[5] Integration of the desorption profile yielded a total acidity of 257 μmol.g^{-1}.

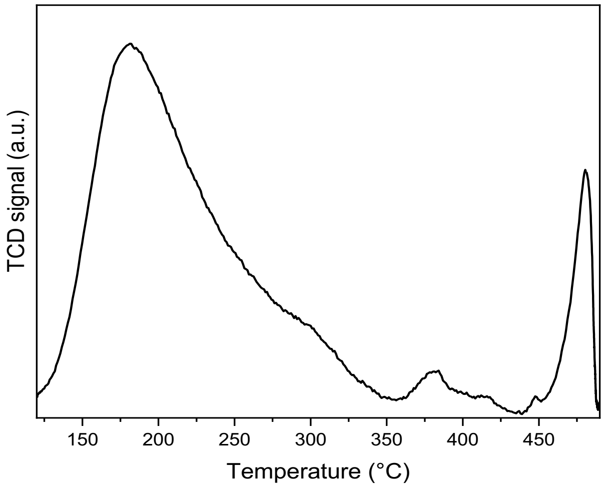

Figure S8: TPD-NH_3 of the MIL-53(Al) catalysts.

4.1.3.1) MIL-53(Al) Catalyst screening:

Catalyst screening was performed to evaluate the efficiency of various acid catalysts in the reaction including precursors $AlCl_3$ and terephthalic acid under identical reaction conditions, along with a control experiment in the absence of catalyst.

Table S1: Catalyst screening.

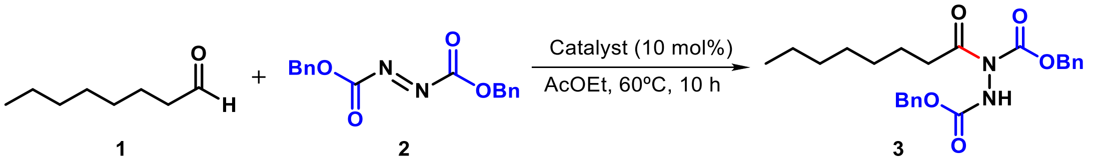

1 2 3

Entry	Catalyst	Yield (%)[a]
1	Aluminum Chloride	40
2	Terephthalic acid	35
3	Amberlyst 15 Ion Exchange	28
4	Amberlite IR-120 (Na) Ion Exchange	41
5	β-zeolite	44
6	Mordenite zeolite	32
7	Without	37
8	MIL-53	97
9	MIL-53 / N_2 atmosphere	36

[a] Isolated yield

The results (Table S2) show that acid catalysts such as $AlCl_3$ and ion-exchange resins afforded moderate yields (28 – 44 %), similar to or slightly better than the blank reaction (37 %), indicating limited activity under the applied conditions. Notably, MIL-53(Al) outperformed all other catalysts, delivering a 97 % isolated yield, highlighting its superior catalytic activity and efficiency in this reaction.

Radical trapping experiments with TEMPO. To investigate the reaction mechanism and evaluate the possible involvement of radical intermediates, radical trapping experiments were performed using 2,2,6,6-tetramethylpiperidine-1-oxyl (TEMPO) as a radical scavenger (Scheme S4).

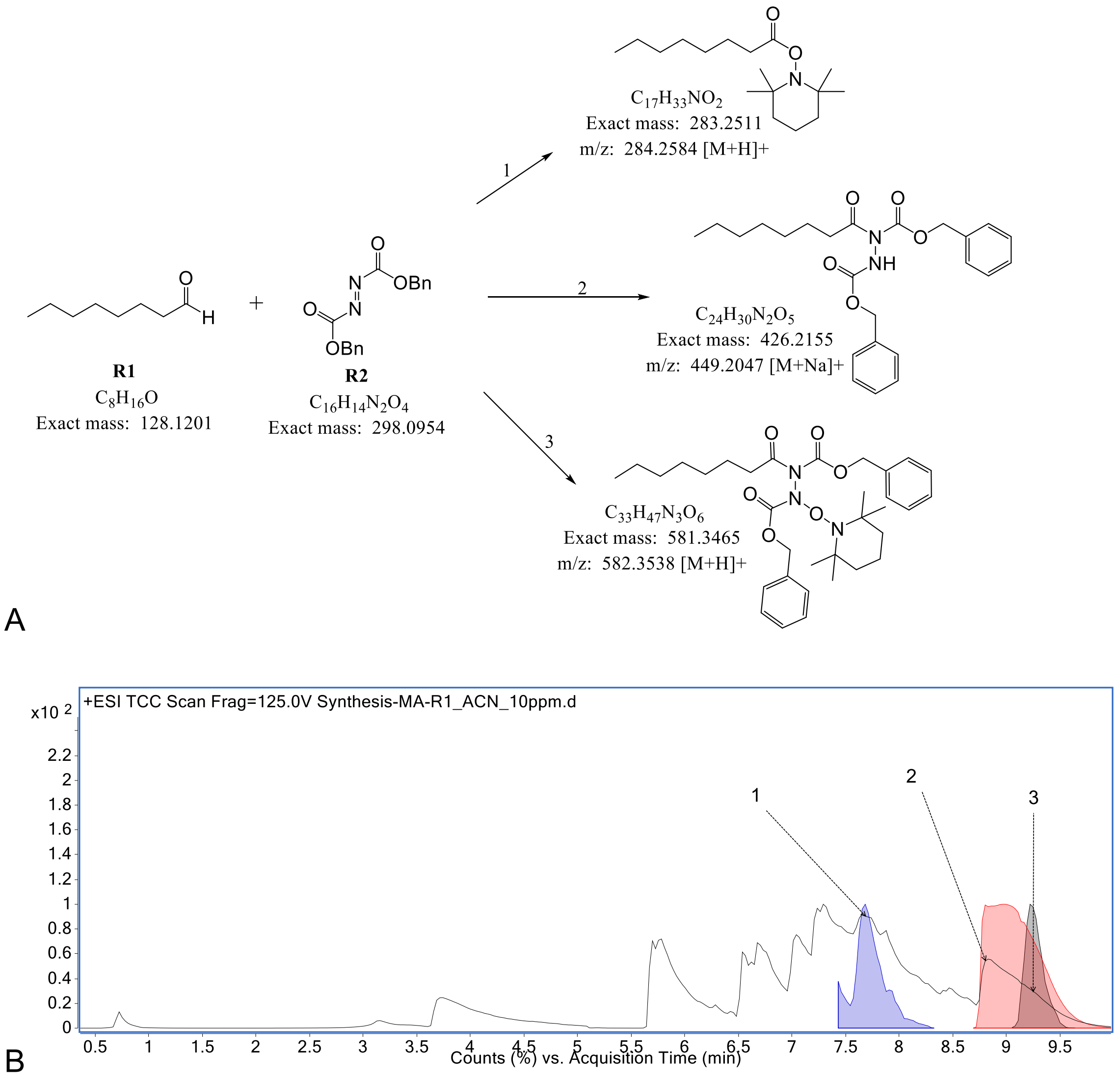

Scheme S4: A) Radical trapping experiment using TEMPO as a scavenger. B) Overlay of the extracted ion chromatograms of the possible products obtained

Trapping radicals by TEMPO 1

When the reaction was carried out under the standard conditions in the presence of TEMPO, the expected product was not detected by TLC monitoring, and only trace signals corresponding to the product were observed by mass spectrometry (Figure S10 to S12). The absence of a clear TLC spot, combined with the near-complete suppression of the product and the detection of only residual amounts by MS, strongly suggests that the transformation predominantly proceeds through a radical pathway, and that TEMPO efficiently quenches the reactive radical intermediates involved in the process.

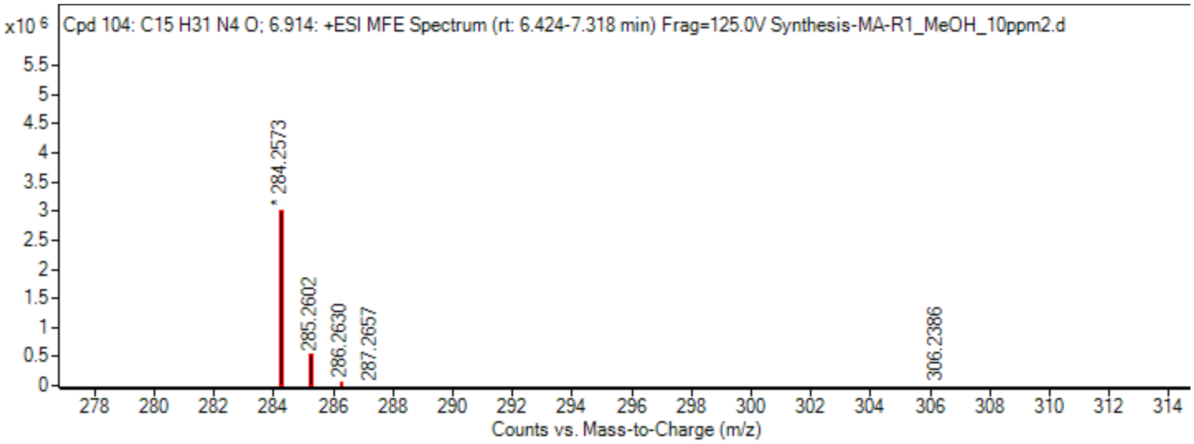

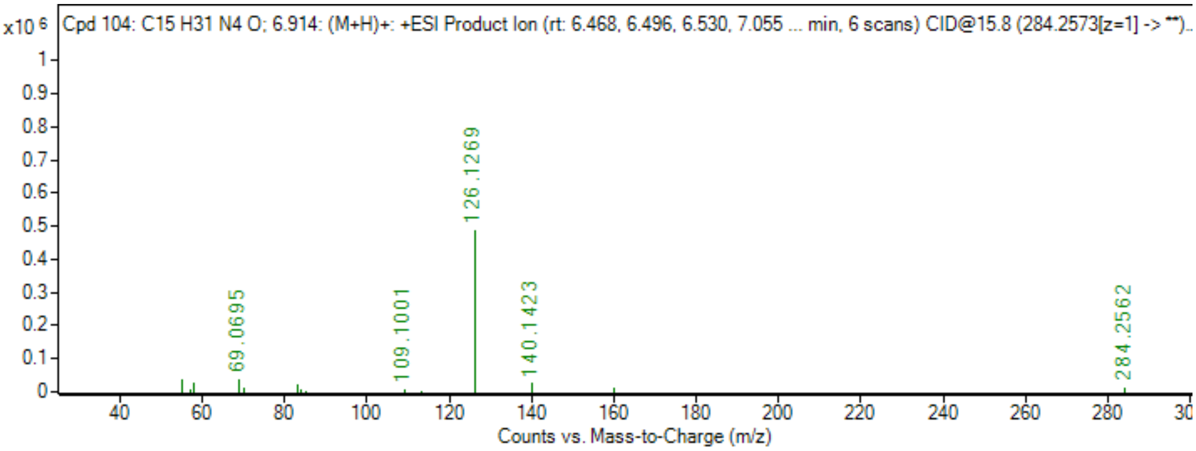

Figure S9: MS and MS/MS acquisition of the precursor ion at m/z 284.2575 $[M+H]^+$, mass error: −3.166

Trapping radicals by TEMPO 3:

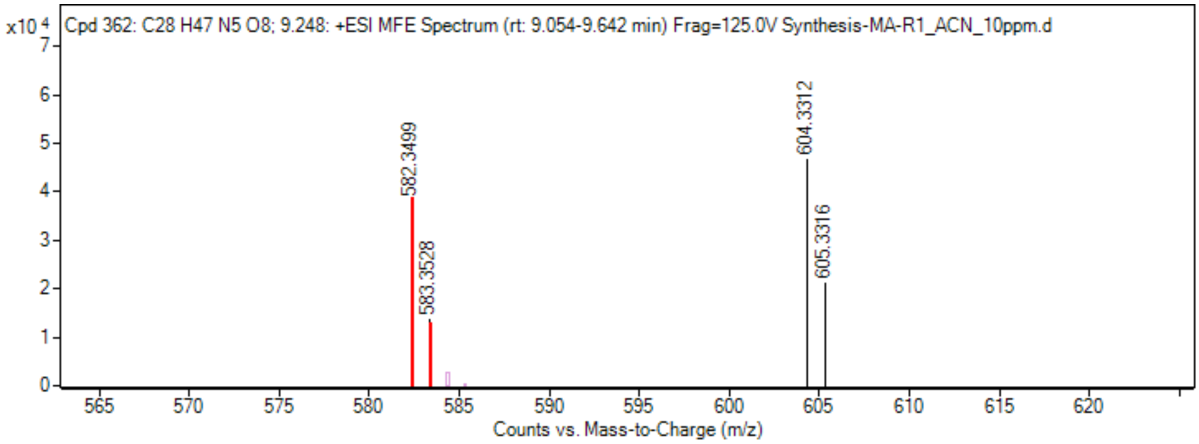

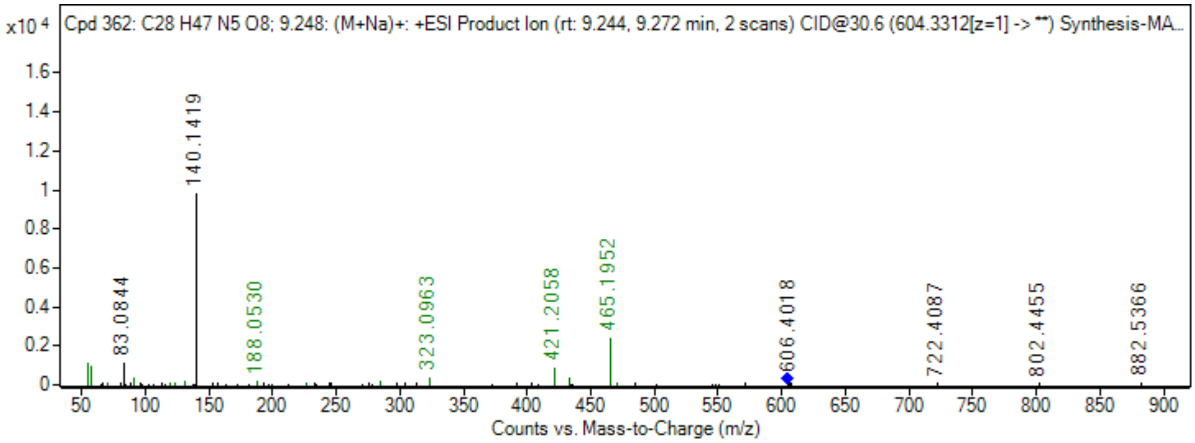

Figure S10. MS and MS/MS acquisition of the precursor ion at m/z 582.3499 [M+H]$^+$, mass error: −6.696.

Product:

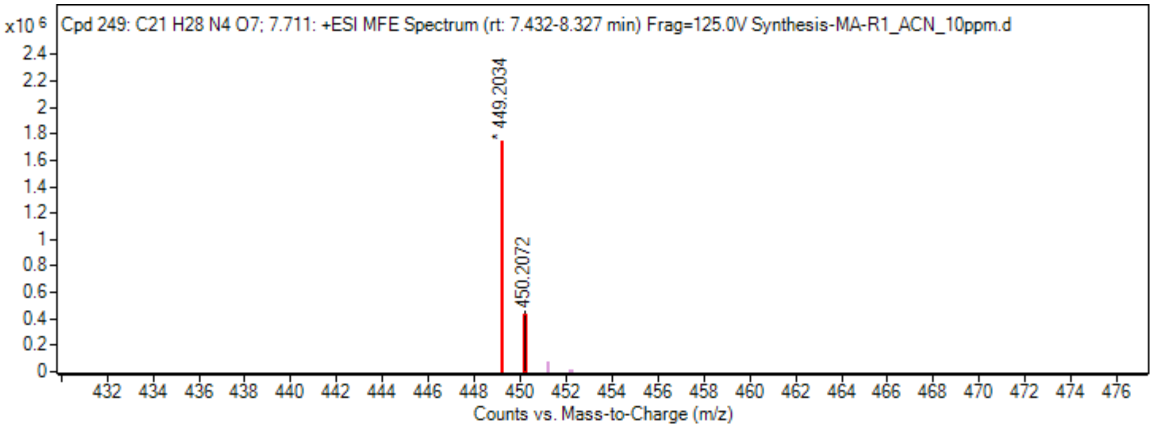

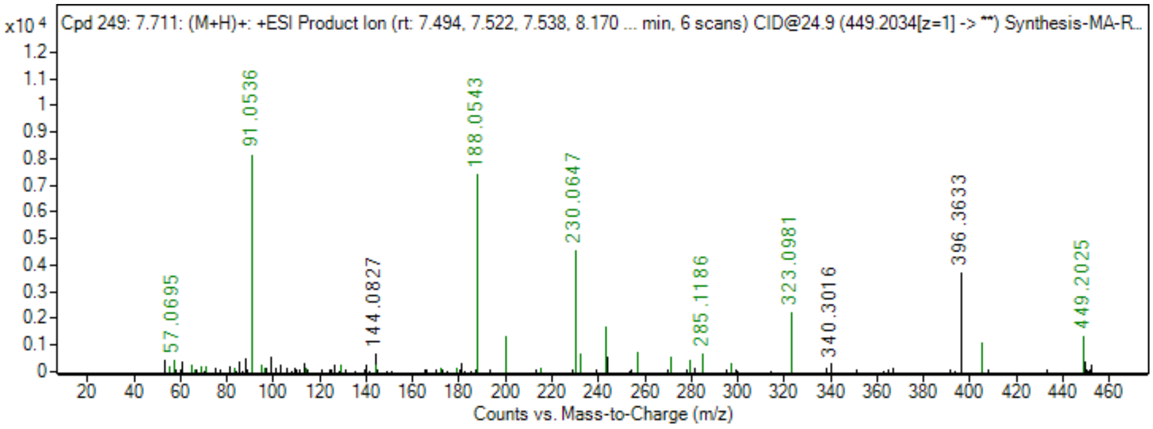

Figure S11. MS and MS/MS acquisition of the precursor ion at m/z 449.2034 [M+Na]$^+$, mass error: −2.894

Experimental evidence supports that the transformation proceeds through a radical pathway, consistent with the observed products and trapping experiments (Scheme S5).

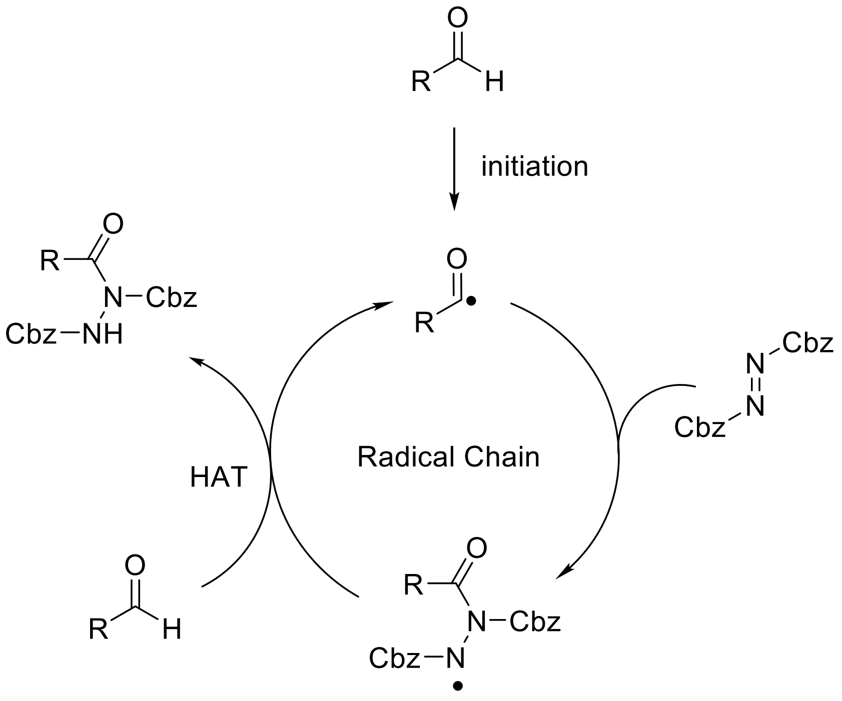

Scheme S5. Proposed radical pathway confirmed by trapping experiments

Atomic Absorption Spectroscopy (AAS) analysis of Al in products and catalyst

Following the confirmation of a radical pathway, atomic absorption spectroscopy (AAS) was performed to evaluate the possible leaching of aluminum from the MIL-53(Al) catalyst into the product (table S3). For this purpose, both the reaction product (**3a**) and the digested **MIL-53(Al)** samples were analyzed. Samples of Product **3a** were subjected to microwave-assisted acid digestion, while the MOF was fully digested under the same conditions to quantify its total Al content. In the case of Product **3a**, no aluminum was detected (ND) across triplicate measurements, with mean absorbance values close to baseline (0.0001–0.0009). These results indicate that the product is free of Al contamination from the catalyst and does not leach into the organic phase. By contrast, digested MIL-53(Al) samples showed high Al concentrations, averaging ca. **145–147 mg L**$^{-1}$ with low %RSD values (1.69–3.30). Overall, these results demonstrate that aluminum remains confined within the MIL-53(Al) catalyst during the

reaction, and the catalytic activity proceeds without metal leaching into the product.

Table S2: AAS Data.

Sample label	Conc. (mg.L^{-1})	%RDS	Mean
Cal Blank	-----	HIGH	0.0018
Standard 1	6.250	5.90	0.0193
Standard 2	12.500	1.92	0.0375
Standard 3	25.000	3.18	0.0653
Standard 5	50.000	1.32	0.1170
Standard 6	75.000	1.51	0.1614
bco	ND	HIGH	-0.0001
**Product 3a**	ND	HIGH	0.0006
**Product 3a**	ND	HIGH	0.0001
**Product 3a**	ND	HIGH	0.0009
**MIL-53**	145.197.188	1.69	0.0853
**MIL-53**	146.676.625	2.26	0.0861
**MIL-53**	144.573.391	3.30	0.0850
bco	ND	HIGH	-0.0001
STD 25.0 ppm	25.035	2.17	0.0623

4.1.4) MIL-53(Al) after reuse

A marginal reduction in catalytic efficiency (~5%) was observed after the fourth cycle (Figure S8 A), highlights the robustness of MIL-53(Al) and its ability to undergo repeated use with minimal loss of activity. PXRD analysis performed after reuse (Figure S13 B) confirms that the crystalline structure of the MOF remains preserved, with no detectable changes in diffraction peak positions or intensities over multiple cycles. degradation. In addition, SEM imaging after the fourth catalytic cycle reveals that MIL-53(Al) retains its characteristic rod-shaped morphology (Figure S13 D). The particle arrangement remains comparable to that of the fresh material (Figure S13 C), with no significant changes in shape or size. These results indicate that the physical integrity of MIL-53(Al) is largely maintained after repeated catalytic applications. A sharper drop in cycle 5 (78%) indicates a more pronounced loss of catalytic efficiency, likely caused by progressive pore obstruction or structural deterioration. By cycle 6,

the yield falls to 45%, evidencing substantial deactivation. These results suggest that the structural and functional integrity of the catalyst is no longer maintained beyond the fourth or fifth reuse, limiting its practical reusability.

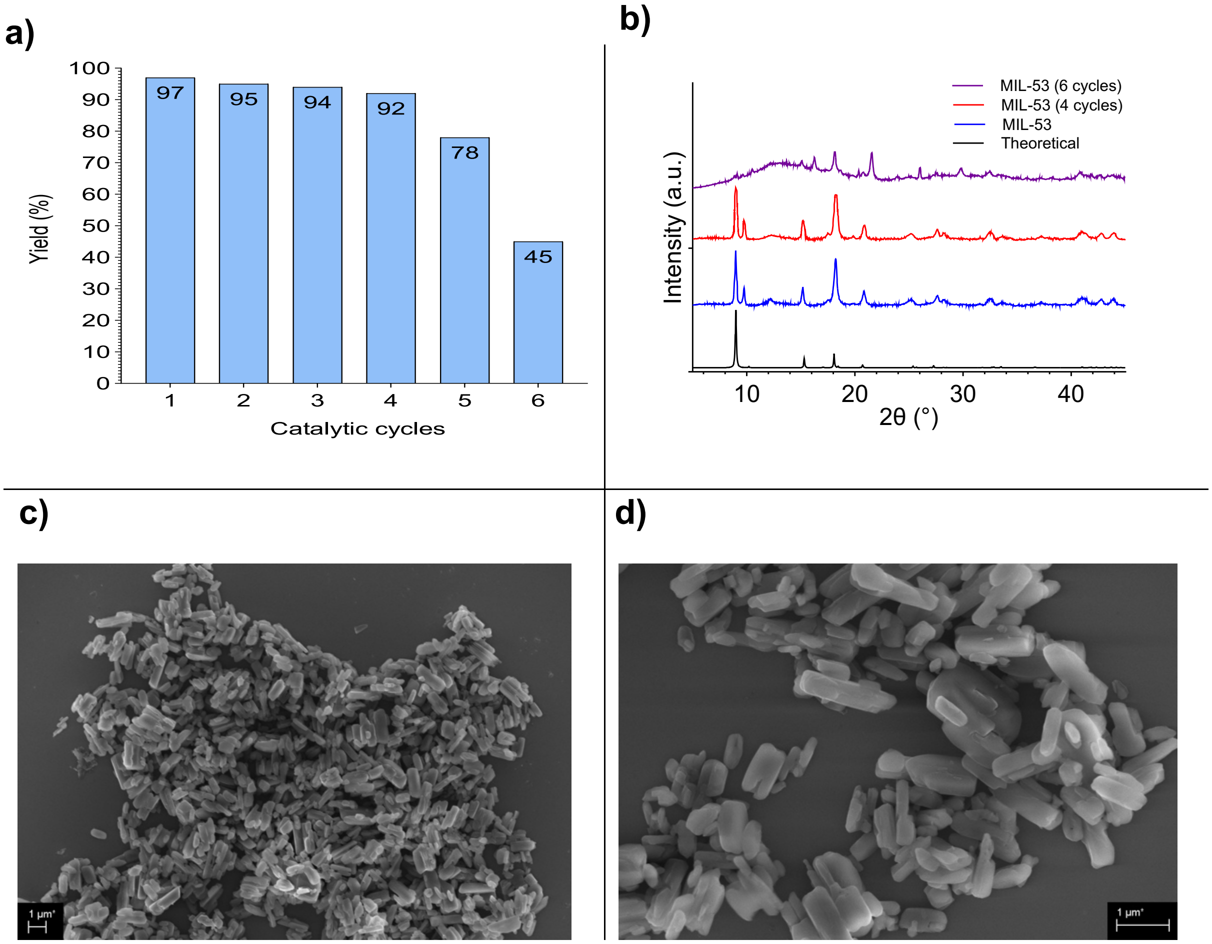

Figure S12. Characterization of the final material in hydrazide synthesis. a) Product yield after 4 catalytic cycles. b) PXRD pattern of reused MIL-53(Al) (red) compared with the fresh material (blue). c) SEM image of fresh MIL-53(Al). (D) SEM image of MIL-53(Al) after the fourth catalytic cycle.

5) NMR Spectroscopic Data of Compounds

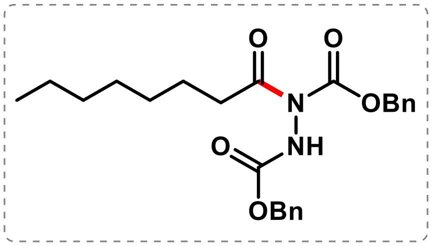

Dibenzyl 1-octanoylhydrazine-1,2-dicarboxylate (3a) was synthesized according to general procedure 3.2. Purification by flash column chromatography (hexanes/EtOAc, 7:3) afforded the title compound as a white solid (MP = 76°C) in 97% yield (82.7 mg). **^{1}H NMR (400 MHz, $CDCl_3$)** δ 7.34 (d, *J* = 5.3 Hz, 10H), 7.04 (s, 1H), 5.24 (s, 2H), 5.17 (s, 2H), 2.90 (t, *J* = 7.7 Hz, 2H), 1.78 - 1.55 (m, 2H), 1.30 (m, 8H), 0.95 – 0.77 (m, 3H). **^{13}C NMR** (101 MHz, $CDCl_3$) δ 173.8, 155.5, 153.1, 135.5, 134.8, 128.7, 128.7, 128.6, 128.5, 128.2, 69.2, 68.1, 37.1, 31.8, 29.1, 24.6, 22.7, 14.2. **MS(ESI)**: *m*/*z* calcd. for $C_{24}H_{30}N_2O_5$ $[M + Na]^+$ 449.2047, found 449.1636. Chromatographic analysis. UPC^2: λ 210 nm, Retention time: 1.98 min, Purity: 99%.

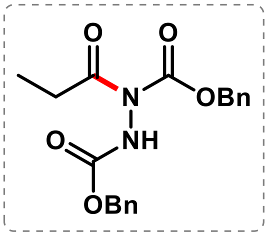

Dibenzyl 1-propionylhydrazine-1,2-dicarboxylate (3b) was synthesized according to general procedure 3.2. Purification by flash column chromatography (hexanes/EtOAc, 7:3) afforded the title compound as a light-yellow oil in 95% yield (67.8 mg) **^{1}H NMR (400 MHz, $CDCl_3$)** δ 7.34 (s, 10H), 6.95 (s, 1H), 5.24 (s, 2H), 5.17 (s, 2H), 2.93 (q, *J* = 8.4, 7.4 Hz, 2H), 1.16 (t, *J* = 7.2 Hz, 3H). **^{13}C NMR (101 MHz, $CDCl_3$)** δ 174.6, 155.5, 153.2, 135.5, 134.8, 128.8, 128.7, 128.7, 128.5, 128.2, 69.3, 68.2, 30.7, 8.9. **MS(ESI):** *m*/*z* calcd. for $C_{19}H_{20}N_2O_5$ $[M + Na]^+$ 379.1264, found 379.1216. Chromatographic analysis. UPC^2: λ 210 nm, Retention time: 1.7 min, Purity: 98%.

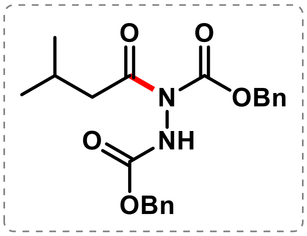

Dibenzyl 1-(3-methylbutanoyl)hydrazine-1,2-dicarboxylate (3c) was synthesized according to general procedure 3.2. Purification by flash column chromatography (hexanes/EtOAc, 7:3) afforded the title compound as a white solid (MP = 91°C) in 87% yield (61.5 mg). **^{1}H NMR (400 MHz, $CDCl_3$)** δ 7.34 (s, 10H), 6.80 (s, 1H), 5.23 (s, 1H), 5.18 (s, 1H), 2.88 – 2.77 (m, 2H), 2.19 (dd, *J* = 10.9, 6.1 Hz, 1H), 0.96 (d, *J* = 6.3 Hz, 6H). **^{13}C NMR (101 MHz, $CDCl_3$)** δ 153.2, 135.5, 134.9, 128.8, 128.8, 128.7, 128.6, 128.3, 69.3, 68.3, 45.7, 25.4, 22.6. **MS(ESI):** *m*/*z* calcd. for $C_{21}H_{24}N_2O_5$ $[M + Na]^+$ 407.1577, found 407.1316. Chromatographic analysis. UPC^2: λ 210 nm, Retention time: 1.53 min, Purity: 98%.

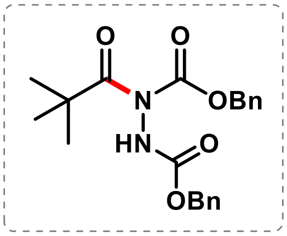

Dibenzyl 1-pivaloylhydrazine-1,2-dicarboxylate (3d) was synthesized according to the general procedure 3.2. Purification by flash column chromatography (hexanes/EtOAc, 7:3) afforded the title compound as a colorless oil in 81% yield (62.3 mg). **^{1}H NMR (400 MHz, $CDCl_3$)** δ 7.36 – 7.24 (m, 10H), 7.10 (s, 1H), 5.25 (s, 2H), 5.17 (s, 2H), 1.30 (s, 9H). **^{13}C NMR (101 MHz, $CDCl_3$)** δ 156.9, 156.1, 153.8, 136.2, 134.9, 128.7, 128.6, 128.5, 128.4, 128.3, 128.2, 128.1, 127.9, 127.1, 69.4, 68.3, 67.7, 42.2, 28.3, 27.5, 27.1. **MS(ESI):** *m*/*z* calcd. for $C_{21}H_{24}N_2O_5$ $[M + Na]^+$ 407.1577, found 407.2336. Chromatographic analysis. UPC^2: λ 210 nm, Retention time: 1.5 min, Purity: 97%.

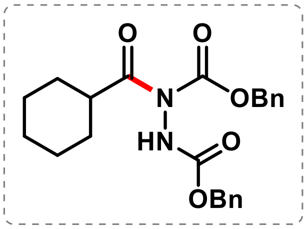

Dibenzyl 1-(cyclohexanecarbonyl)hydrazine-1,2-dicarboxylate (3e) was synthesized according to general procedure 3.2. Purification by flash column chromatography (hexanes/EtOAc, 7:3) afforded the title compound as a white solid (MP = 122°C) in 94% yield (77.2 mg). **^{1}H NMR (400 MHz, $CDCl_3$)** δ 7.34 (s, 10H), 7.10 (s, 1H), 5.24 (s, 2H), 5.16 (s, 2H), 3.50 – 3.31 (m, 1H), 2.00 – 1.88 (m, 2H), 1.81 – 1.68 (m, 2H), 1.44 (ddd, *J* = 15.4, 11.5, 5.4 Hz, 2H)1.25 (dd, (dd, *J* = 12.1, 7.5 Hz, 4H). **^{13}C NMR (101 MHz, $CDCl_3$)** δ 180.9, 155.6, 153.1, 134.8, 128.7, 128.6, 128.4, 128.2, 69.2, 68.1, 44.0, 42.8, 29.4, 28.9, 29.4, 25.8, 25.6, 25.4. **MS(ESI):** *m*/*z* calcd. for $C_{23}H_{26}N_2O_5$ $[M + Na]^+$ 433.1734, found 434.1319. Chromatographic analysis. UPC^2: λ 210 nm, Retention time: 2.07 min, Purity: 99%.

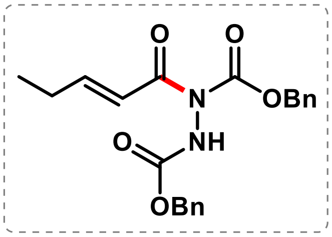

Dibenzyl (*E*)-1-(pent-2-enoyl)hydrazine-1,2-dicarboxylate (3f) was synthesized according to general procedure 3.2. Purification by flash column chromatography (hexanes/EtOAc, 7:3) afforded the title compound as a white solid (MP = 85°C) in 82% yield (62.7 mg). **^{1}H NMR (400 MHz, $CDCl_3$)** δ 7.34 (s, 10H), 7.16 (dt, J = 15.3, 6.4 Hz, 1H), 7.02 (s, 1H), 6.89 (d, J = 15.4 Hz, 1H), 5.25 (s, 2H), 5.17 (s, 2H), 2.31 (m, 2H), 1.06 (t, J = 7.4 Hz, 3H); **^{13}C NMR (101 MHz, $CDCl_3$)** δ 166.3, 155.6, 153.3, 135.5, 134.8, 128.8, 128.7, 128.7, 128.5, 128.2, 120.9, 69.4, 68.2, 25.9, 12.2. **MS(ESI):** *m*/*z* calcd. for $C_{21}H_{22}N_2O_5$ [M + Na]+ 405.1421, found 405.9732. Chromatographic analysis. UPC^2: λ 210 nm, Retention time: 1.71 min, Purity: 96%

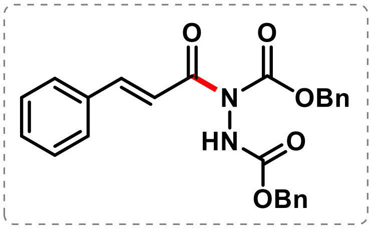

Dibenzyl 1-cinnamoylhydrazine-1,2-dicarboxylate (3g) was synthesized according to general procedure 3.2. Purification by flash column chromatography (hexanes/EtOAc, 7:3) afforded the title compound as a light-yellow oil in 60% yield (51.30 mg). **^{1}H NMR (400 MHz, $CDCl_3$)** δ 7.81 (d, *J* = 15.6 Hz, 1H), 7.51 (d, *J* = 7.2 Hz 2H), 7.36 (t, *J* = 15.6 Hz, 14H), 7.11 – 7.01 (m, 1H), 5.29 (s, 2H), 5.20 (s, 2H). **^{13}C NMR (101 MHz, $CDCl_3$)** δ 166.3, 155.5, 153.2, 146.6, 135.4, 134.6, 134.5, 130.6, 128.8, 128.7, 128.7, 128.6, 128,5, 128.4, 128.2, 118.4, 69.7, 68.1. **MS(ESI):** *m*/*z* calcd. for $C_{25}H_{21}N_2O_5$ [M - H]$^+$ 429.1456, found 429.2318. Chromatographic analysis. UPC^2: λ 289 nm, Retention time: 4.27 min, Purity: 99%.

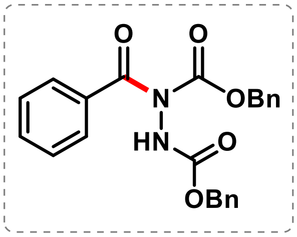

Dibenzyl 1-benzoylhydrazine-1,2-dicarboxylate (3h) was synthesized according to general procedure 3.2. Purification by flash column chromatography (hexanes/EtOAc, 7:3) afforded the title compound as a yellow oil in 61% yield (49.4 mg). **^{1}H NMR (400 MHz, $CDCl_3$)** δ 7.59 (d, *J* = 7.5 Hz, 1H), 7.43 – 7.35 (m, 2H), 7.26 – 7.15 (m, 10H), 6.99 (d, *J* = 7.9 Hz, 2H), 5.10 (s, 2H), 5.02 (s, 2H). **^{13}C NMR (101 MHz, $CDCl_3$)** δ 170.9, 155.5, 153.4, 134.2, 133.6, 132.2, 130.2, 128.6, 128.6, 128.5, 128.5, 128.4, 128.2, 128.2, 69.5, 68.3. **MS(ESI):** *m*/*z* calcd. for $C_{23}H_{20}N_2O_5$ [M + Na]$^+$ 427.1264, found 427.8559. Chromatographic analysis. UPC^2: λ 210 nm, Retention time: 2.83 min, Purity: 95%.

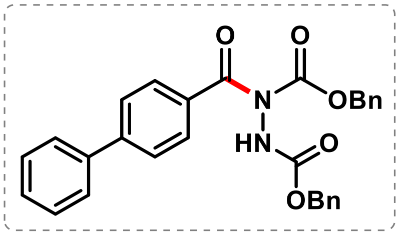

Dibenzyl 1-([1,1'-biphenyl]-4-carbonyl)hydrazine-1,2-dicarboxylate (3i) was synthesized according to general procedure 3.2. Purification by flash column chromatography (hexanes/EtOAc, 7:3) afforded the title compound as a white solid (MP = 84°C) in 54% yield (48.1 mg). **^{1}H NMR (400 MHz, $CDCl_3$)** δ 7.67 (s, 1H), 7.49 (d, *J* = 7.0 Hz, 4H), 7.38 (t, *J* = 7.4 Hz, 2H), 7.32 (d, *J* = 7.3 Hz, 1H), 7.24 (s, 10H), 6.99 (d, *J* = 5.9 Hz, 2H), 5.11 (s, 2H), 5.04 (s, 2H). **^{13}C NMR (101 MHz, $CDCl_3$)** δ 153.5, 145.2, 140.0, 135.4, 134.8, 134.3, 129.2, 129.0, 128.8, 128.7, 128.7, 128.6, 128.6, 128.3, 128.3, 127.7, 127.4, 127.1, 127.0, 69.7, 68.4. **MS(ESI):** *m*/*z* calcd. for $C_{29}H_{24}N_2O_5$ [M + Na]$^+$ 503.1577, found 504.2135. Chromatographic analysis. UPC^2: λ 210 nm, Retention time: 5.5 min, Purity: 95%

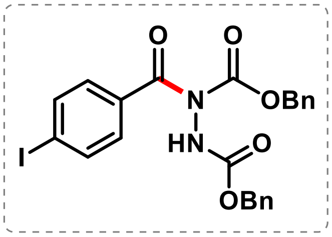

Dibenzyl 1-(4-iodobenzoyl)hydrazine-1,2-dicarboxylate (3j) was synthesized according to general procedure 3.2. Purification by flash column chromatography (hexanes/EtOAc, 7:3) afforded the title compound as a yellow solid (MP = 81°C) in 58% yield (61.5 mg). **^{1}H NMR (400 MHz, CDCl$_3$)** δ 7.67 (d, *J* = 8.3 Hz, 2H), 7.33 (qd, *J* = 10.3, 8.8, 4.0 Hz, 10H), 7.14 – 7.04 (m, 2H), 5.20 (s, 2H), 5.12 (s, 2H). **^{13}C NMR (101 MHz, CDCl$_3$)** δ 153.2, 137.6, 134.1, 129.8, 128.9, 128.8, 128.8, 128.7, 128.5, 128.3, 99.6, 69.9, 68.5. **MS(ESI):** *m*/*z* calcd. for $C_{23}H_{19}IN_2O_5$ [M + Na]$^+$ 553.0231, found 553.7363. Chromatographic analysis. UPC^2: λ 210 nm, Retention time: 4.22 min, Purity: 99%

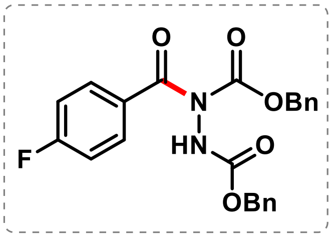

Dibenzyl 1-(4-fluorobenzoyl)hydrazine-1,2-dicarboxylate (3k) was synthesized according to general procedure 3.2. Purification by flash column chromatography (hexanes/EtOAc, 7:3) afforded the title compound as a colorless oil in 86% yield (72.7 mg). **^{1}H NMR (400 MHz, CDCl$_3$)** δ 7.69 88 (t, *J* = 6.9 Hz, 1H), 7.40 – 7.28 (m, 10H), 7.17 – 7.04 (m, 1H), 6.98 (d, *J* = 8.5 Hz, 2H), 5.18 (s, 2H), 5.12 (s, 2H). **^{13}C NMR (101 MHz, CDCl$_3$)** δ 169.9, 166.4, 163.9, 153.4, 135.3, 134.8, 134.2, 132.9, 132.8, 131.2, 131.1, 128.8, 128.7, 128.6, 128.6, 128.4, 128.3, 115.6, 115.4, 69.7, 68.4. **MS(ESI):** *m*/*z* calcd. for $C_{23}H_{19}FN_2O_5$ [M + Na]$^+$ 454.1055, found. 454.1110. Chromatographic analysis. UPC^2: λ 210 nm, Retention time: 2.18 min, Purity: 95%

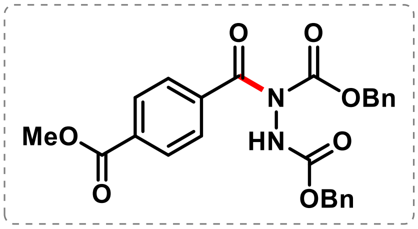

Dibenzyl 1-(4-(methoxycarbonyl)benzoyl)hydrazine-1,2-dicarboxylate (3l) was synthesized according to general procedure 3.2. Purification by flash column chromatography (hexanes/EtOAc, 7:3) afforded the title compound as a colorless oil in 46% yield (42.50 mg). **^{1}H NMR (400 MHz, CDCl$_3$)** δ 7.98 (d, *J* = 8.0 Hz, 1H), 7.67 (d, *J* = 7.9 Hz, 1H), 7.34 (s, 10H), 7.09 (d, *J* = 7.2 Hz, 2H), 6.62 (s, 1H), 5.20 (s, 2H), 5.16 (s, 2H), 3.94 (s, 3H). **^{13}C NMR (101 MHz, CDCl$_3$)** δ 166.1, 156.5, 155.4, 153.0, 138.59, 135.5, 135.1, 133.9, 132.8, 129.4, 128.7, 128.6, 128.6, 128.5, 128.4, 128.4, 128.2, 128.2, 127.8, 69.7, 68.4, 67.9, 52.4.**MS(ESI):** *m*/*z* calcd. for $C_{25}H_{21}N_2O_7$ [M - H]$^+$ 461.1354, found 461.2932. Chromatographic analysis. UPC^2: λ 241 nm, Retention time: 2.94 min, Purity: 99%.

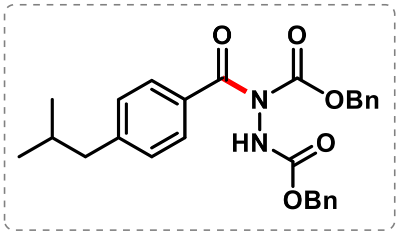

Dibenzyl 1-(4-isobutylbenzoyl)hydrazine-1,2-dicarboxylate (3m) was synthesized according to general procedure 3.2. Purification by flash column chromatography (hexanes/EtOAc, 7:3) afforded the title compound as a light-light-yellow oil in 68% yield (62.6 mg). **^{1}H NMR (400 MHz, $CDCl_3$)** δ 7.54 (s, 1H), 7.31 – 7.16 (m, 10H), 7.10 – 7.04 (m, 2H), 7.01 (d, *J* = 6.6 Hz, 2H), 5.12 (s, 2H), 5.04 (s, 2H), 2.42 (d, *J* = 7.2 Hz, 2H), 1.80 (s, 1H), 0.82 (d, *J* = 6.6 Hz, 6H). **^{13}C NMR (101 MHz, $CDCl_3$)** δ 155.6, 153.6, 153.2, 147.1, 134.4, 129.1, 128.7, 128.7, 128.6, 128.6, 128.3, 69.5, 68.4, 45.6, 30.2, 22.5. **MS(ESI):** *m*/*z* calcd. for $C_{27}H_{28}N_2O_5$ $[M + Na]^+$ 483.1890, found 483.8834. Chromatographic analysis. UPC^2: λ 210 nm, Retention time: 2.55 min, Purity: 96%

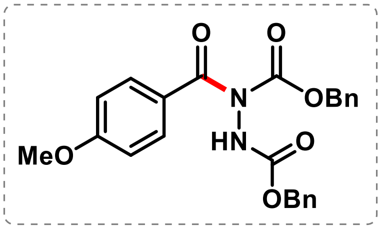

Dibenzyl 1-(4-methoxybenzoyl)hydrazine-1,2-dicarboxylate (3n) was synthesized according to general procedure 3.2. Purification by flash column chromatography (hexanes/EtOAc, 7:3) afforded the title compound as a light-yellow oil in 51% yield (44.50 mg). **^{1}H NMR (400 MHz, $CDCl_3$)** δ 7.70 (d, *J* = 8.3 Hz, 1H), 7.32 (d, *J* = 19.9 Hz, 10H), 7.13 (d, *J* = 5.7 Hz, 2H), 6.83 (d, *J* = 8.4 Hz, 1H), 6.63 (s, 1H), 5.19 (s, 2H), 5.17 (s, 2H), 3.83 (s, 3H). **^{13}C NMR (101 MHz, $CDCl_3$)** δ 163.2, 155.5, 153.6, 135.4, 135,4, 132.2, 131.2,128.6, 128.5, 128.5, 128.4, 128.2, 128.1, 126.2, 113.5, 69.3, 68.2, 67.9, 55.4. **MS(ESI):** *m*/*z* calcd. for $C_{24}H_{21}N_2O_6$ $[M - H]^+$ 433.1405, found 433.2931. Chromatographic analysis. UPC^2: λ 269 nm, Retention time: 3.28 min, Purity: 89%.

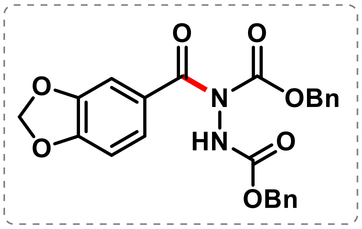

Dibenzyl 1-(benzo[d][1,3]dioxole-5-carbonyl)hydrazine-1,2-dicarboxylate (3o) was synthesized according to general procedure 3.2. Purification by flash column chromatography (hexanes/EtOAc, 7:3) afforded the title compound as a colorless oil in 41% yield (36.30 mg). **^{1}H NMR (400 MHz, $CDCl_3$)** δ 7.45 – 7.22 (m, 10H), 7.20 – 7.03 (m, 3H), 6.74 (d, *J* = 8.0 Hz, 1H), 6.00 (s, 2H), 5.19 (s, 2H), 5.14 (s, 2H). **^{13}C NMR (101 MHz, $CDCl_3$)** δ 170.0, 153.5, 151.4, 147.5, 135.2, 134.3, 128.6, 128.5, 128.5, 128.2, 128.2, 124.7, 109.0, 107.9, 101.85, 69.4, 68.2. **MS(ESI):** *m*/*z* calcd. for $C_{24}H_{20}N_2O_7$ $[M + Na]^+$ 471.1168, found 471.0428. Chromatographic analysis. UPC^2: λ 303 nm, Retention time: 3.26 min, Purity: 97%.

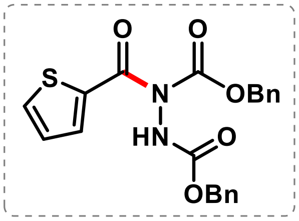

Dibenzyl 1-(thiophene-2-carbonyl)hydrazine-1,2-dicarboxylate (3p). was synthesized according to the general procedure 3.2. Purification by flash column chromatography (hexanes/EtOAc, 7:3) afforded the title compound as a yellow oil in 60% yield (49.3 mg). **^{1}H NMR (400 MHz, CDCl$_3$)** δ 7.84 (s, 1H), 7.58 (dd, *J* = 5.0, 1.3 Hz, 1H), 7.36 – 7.28 (m, 10H), 7.16 (s, 1H), 7.07 – 7.00 (m, 1H), 5.27 (s, 2H), 5.21 (s, 2H). **^{13}C NMR (101 MHz, CDCl$_3$)** δ 136.0, 134.6, 134.1, 128.7, 128.4, 127.5, 69.8, 68.6. **MS(ESI):** *m*/*z* calcd. for $C_{21}H_{18}N_2O_5S$ [M + Na]$^+$ 433.0829, found 433.8619. Chromatographic analysis. UPC^2: λ 210 nm, Retention time: 3.24 min, Purity: 95%

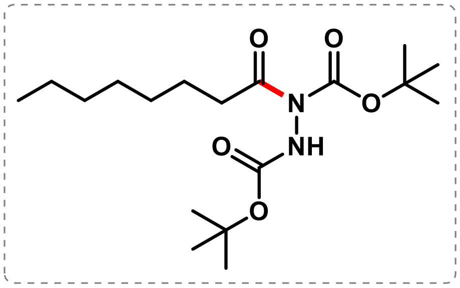

Di-tert-butyl 1-octanoylhydrazine-1,2-dicarboxylate (3q) was synthesized according to general procedure 3.2. Purification by flash column chromatography (hexanes/EtOAc, 7:3) afforded the title compound as a light-yellow oil in 88% yield (68.1 mg). **^{1}H NMR (400 MHz, CDCl$_3$)** δ 6.58 (s, 1H), 2.83 (t, *J* = 7.5 Hz, 2H), 1.68-1.57 (m, 2H), 1.50 (s, 9H), 1.45 (s, 9H), 1.31 – 1.21 (m, 8H), 0.86-0.83 (m, 3H). **^{13}C NMR (101 MHz, CDCl$_3$)** δ 174.2, 154.5, 151.8, 84.2, 81.9, 37.2, 31.8, 29.2, 29.1, 28.2, 28.0, 24.8, 22.7, 14.2. **MS(ESI)**: *m*/*z* calcd. for $C_{18}H_{34}N_2O_5$ [M + Na]$^+$ 381.2360, found 381.2242. Chromatographic analysis. UPC^2: λ 210 nm, Retention time: 0.85 min, Purity: 97%.

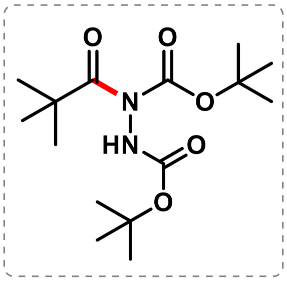

Di-tert-butyl 1-pivaloylhydrazine-1,2-dicarboxylate (3r) was synthesized according to general procedure 3.2. Purification by flash column chromatography (hexanes/EtOAc, 7:3) afforded the title compound as a light-yellow oil in 75% yield (47.5 mg). **^{1}H NMR (400 MHz, CDCl$_3$)** δ 6.50 (s, 1H), 1.51 (s, 9H), 1.47 (s, 9H), 1.30 (s, 9H). **^{13}C NMR (101 MHz, CDCl$_3$)** δ 152.2, 83.9, 59.3, 41.9, 29.7, 28.5, 28.3, 28.2, 28.1, 27.9, 27.5, 27.1. **MS(ESI):** *m*/*z* calcd. for $C_{15}H_{28}N_2O_5$ [M + Na]$^+$ 339.1890, found 339.3657. Chromatographic analysis. UPC^2: λ 210 nm, Retention time: 0.81 min, Purity: 98%.

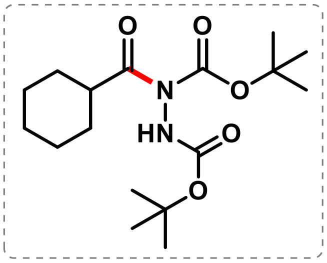

Di-tert-butyl 1-(cyclohexanecarbonyl)hydrazine-1,2-dicarboxylate (3s) was synthesized using general procedure 3.2. Purification by flash column chromatography (hexanes/EtOAc, 7:3) afforded the title compound as a white solid (MP = 144°C, [145°C][9]) in 86% yield (58.9 mg). **^{1}H NMR (400 MHz, $CDCl_3$)** d 6.49 (s, 1H), 1.92 (d, J = 12.3 Hz, 2H), 1.78 (dt, J = 12.6, 3.3 Hz, 2H), 1.72 – 1.64 (m, 2H), 1.53 (s, 9H), 1.53 (s, 9H), 1.47 (s, 9H), 1.28 (ddd, J = 20.9, 10.0, 3.4 Hz, 4H). ^{13}C NMR (126 MHz, $CDCl_3$) δ 182.3, 151.7, 84.2, 81.9, 44.1, 43.0, 29.6, 28.9, 28.2, 28.0, 25.9, 25.8, 25.7, 25.5. **MS(ESI):** *m*/*z* calcd. for $C_{17}H_{30}N_2O_5$ $[M + Na]^+$ 365.2047, found 364.9313. Chromatographic analysis. UPC^2: l 210 nm, Retention time: 0.86 min, Purity: 97%

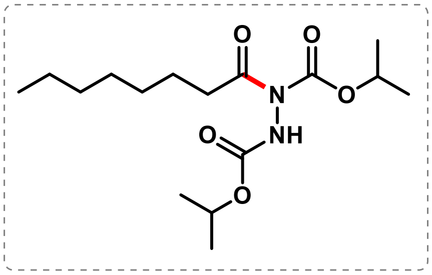

Diisopropyl 1-octanoylhydrazine-1,2-dicarboxylate (3t) was synthesized according to general procedure 3.2. Purification by flash column chromatography (hexanes/EtOAc, 7:3) afforded the title compound as a light yellow oil in 92% yield (60.8 mg).**^{1}H NMR (400 MHz, $CDCl_3$)** δ 6.78 (s, 1H), 4.97 (dhept, J = 25.0, 6.3 Hz, 2H), 2.86 (t, J = 7.6 Hz, 2H), 1.77 – 1.53 (m, 2H), 1.30 (s, 6H), 1.28 (s, 6H), 1.26 – 1.23 (s, 8H), 0.87 – 0.83 (m, 3H). **^{13}C NMR (101 MHz, $CDCl_3$)** δ 174.0, 155.2, 152.8, 72.1, 70.4, 31.8, 29.2, 29.1, 24.7, 22.7, 22.0, 21.8, 14.1. **MS(ESI):** *m*/*z* calcd. for $C_{16}H_{30}N_2O_5$ $[M + Na]^+$ 353.2047, found 353.1956. Chromatographic analysis. UPC^2: λ 210 nm, Retention time: 0.85 min, Purity: 97%.

6) Characterization Spectra of Compounds 3a-3t.

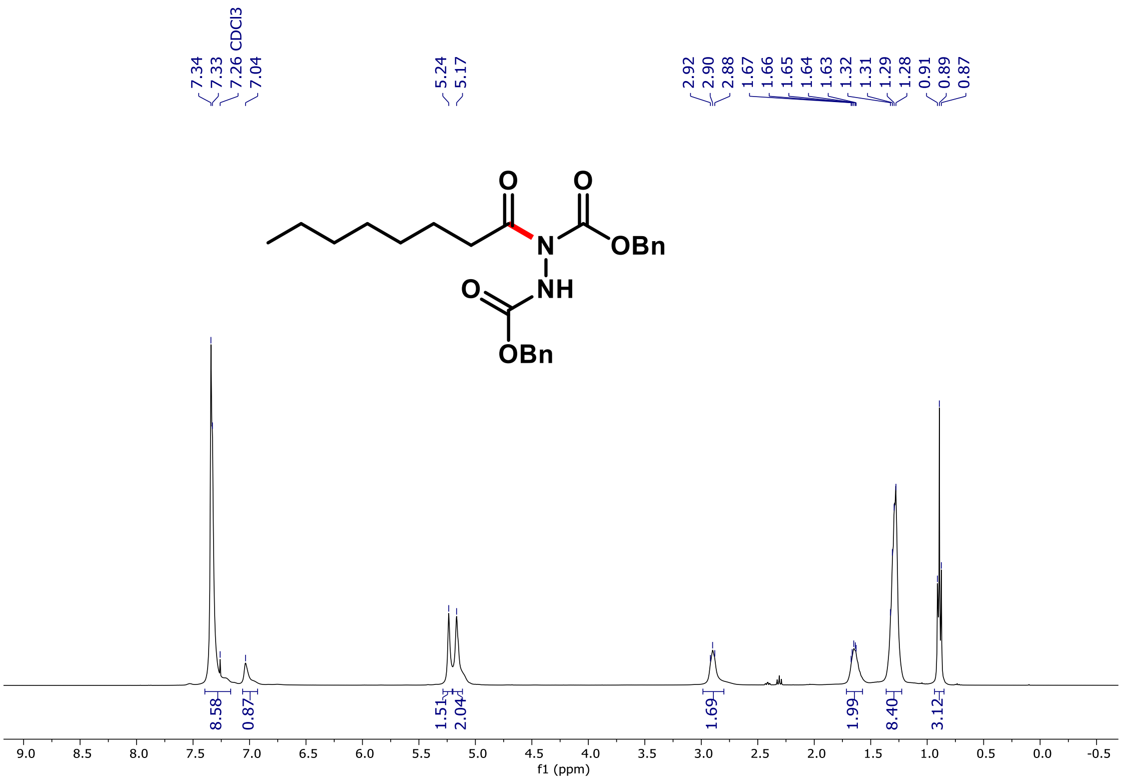

Figure S13. ^{1}H NMR of compound **3a** (400 MHz, $CDCl_3$)

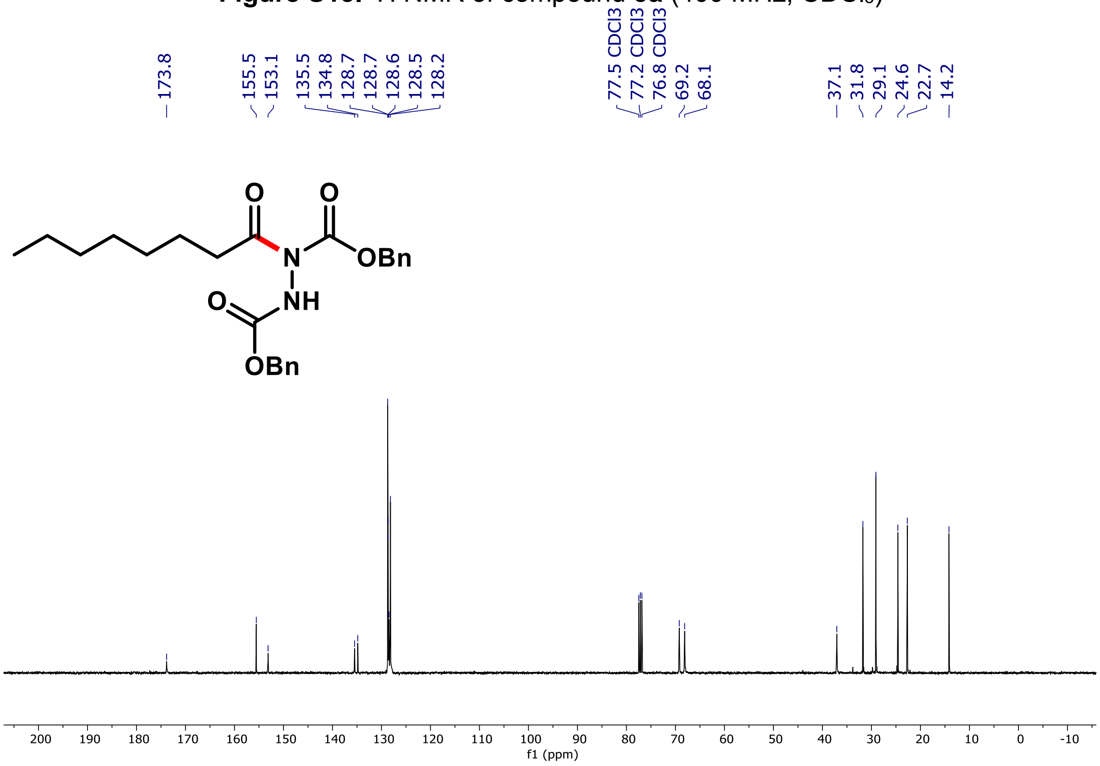

Figure S14. ^{13}C NMR of compound **3a** (101 MHz, $CDCl_3$).

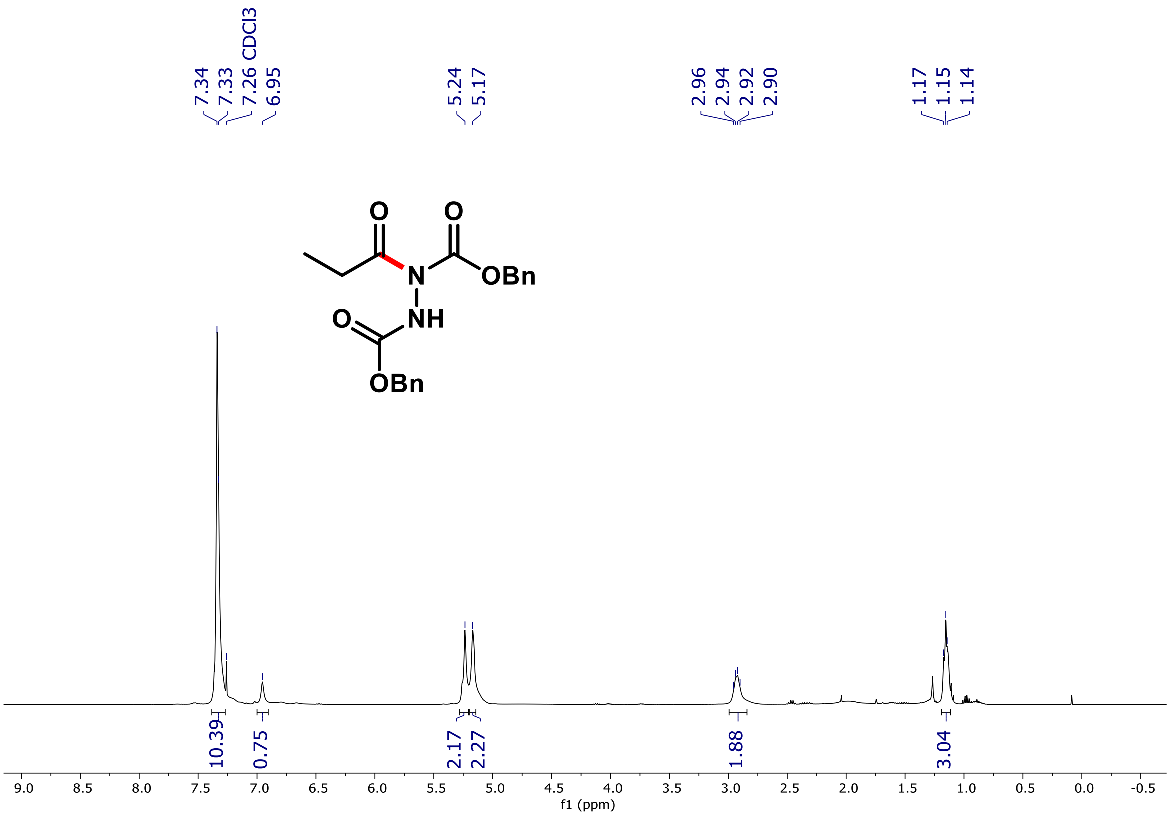

Figure S15. ^{1}H NMR of compound **3b** (400 MHz, $CDCl_3$)

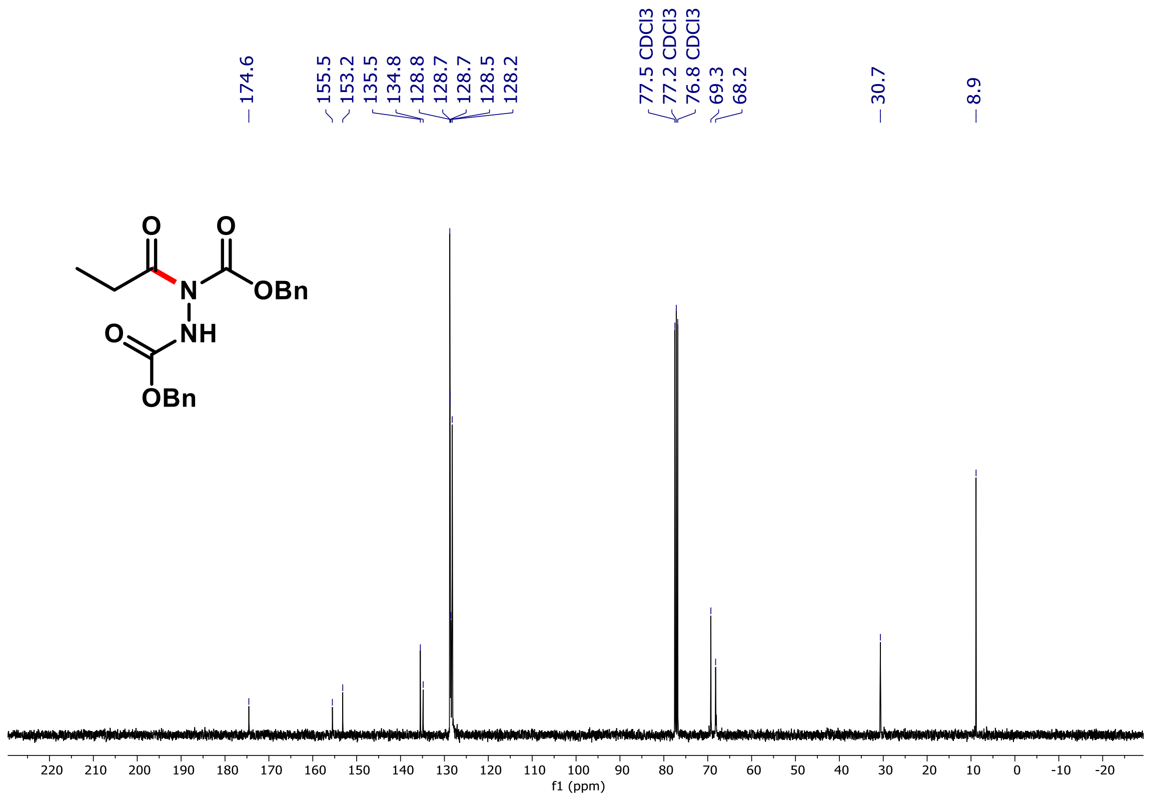

Figure S16. ^{13}C NMR of compound **3b** (101 MHz, $CDCl_3$)

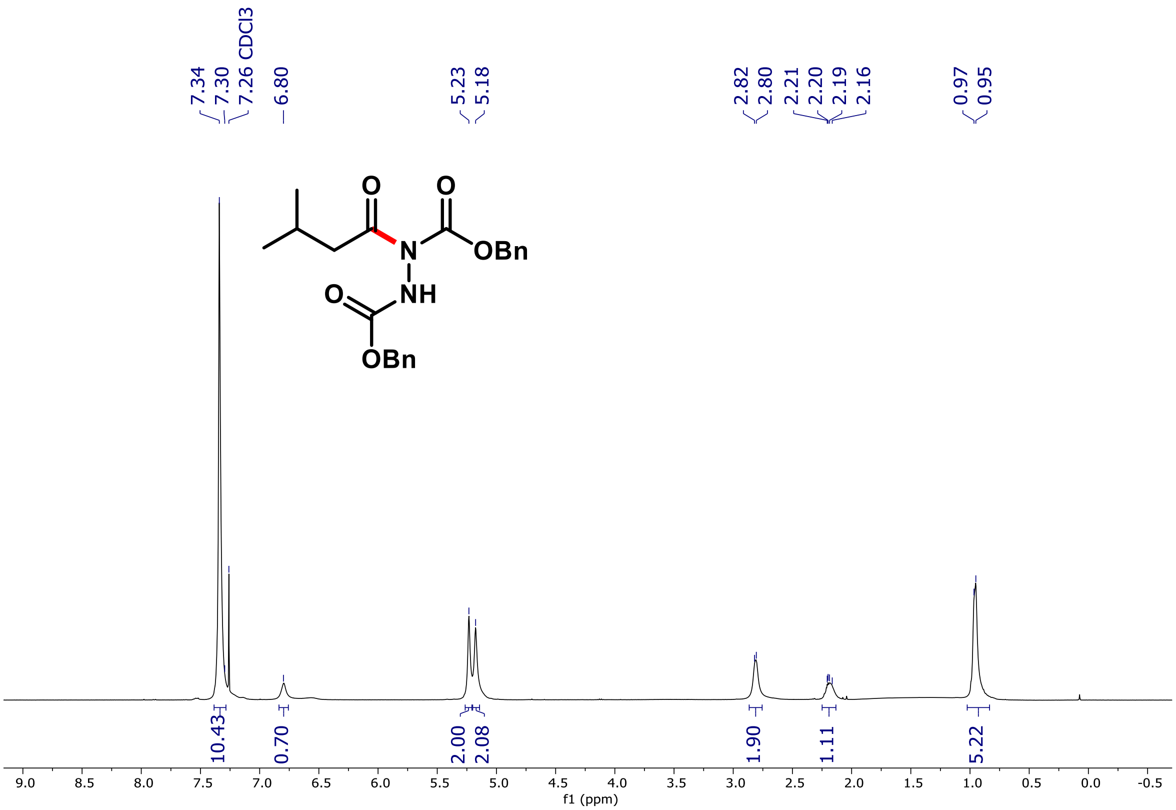

Figure S17. ^{1}H NMR of compound **3c** (400 MHz, $CDCl_3$)

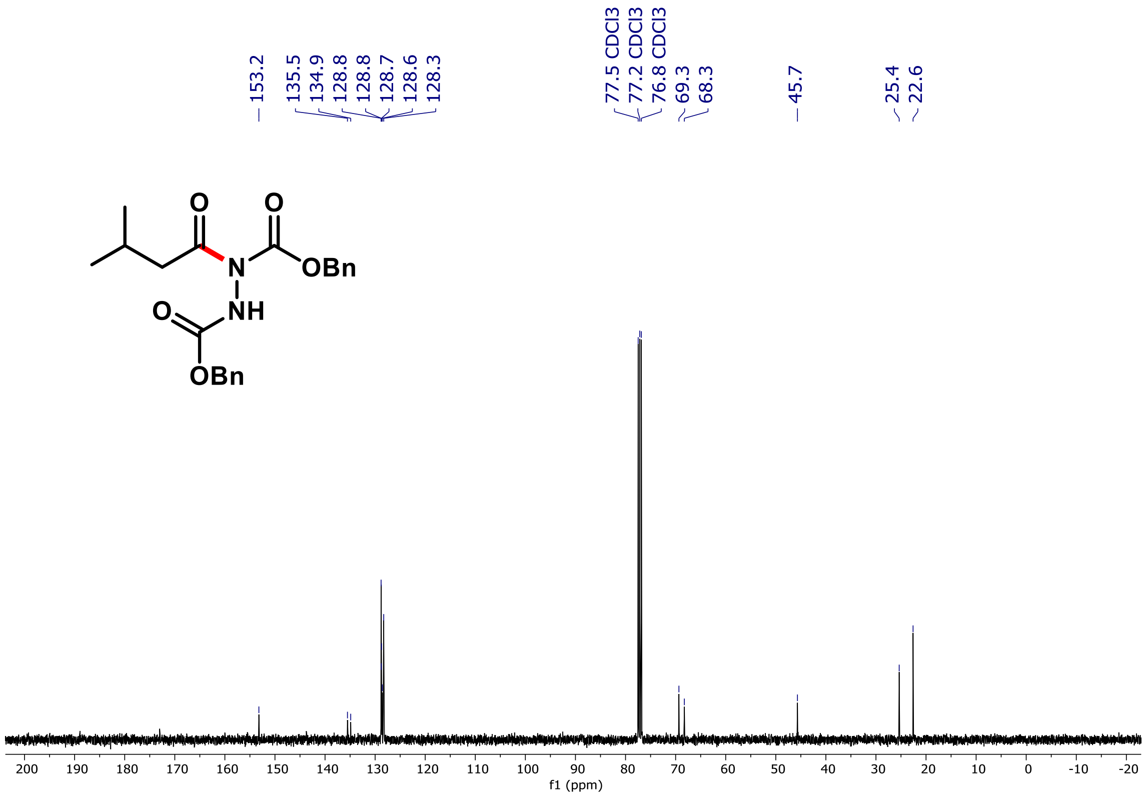

Figure S18. ^{13}C NMR of compound **3c** (101 MHz, $CDCl_3$)

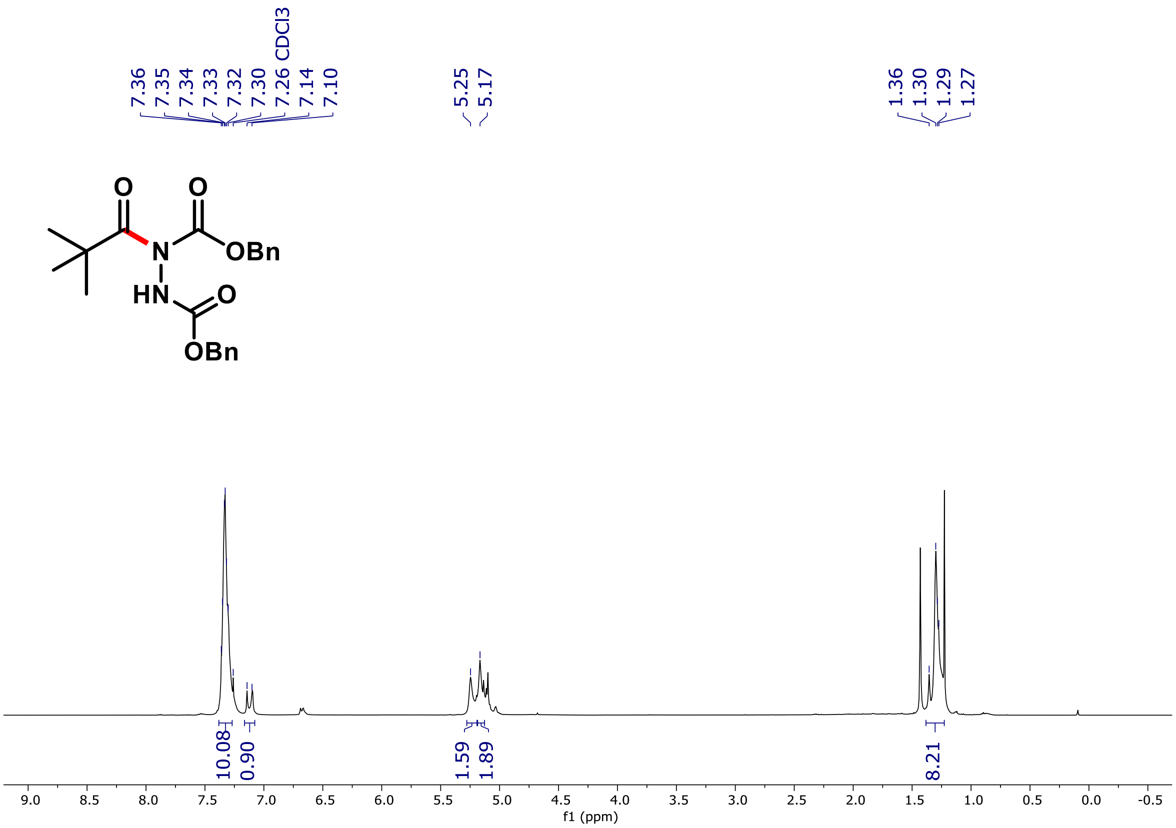

Figure S19. ^{1}H NMR of compound **3d** (400 MHz, $CDCl_3$)

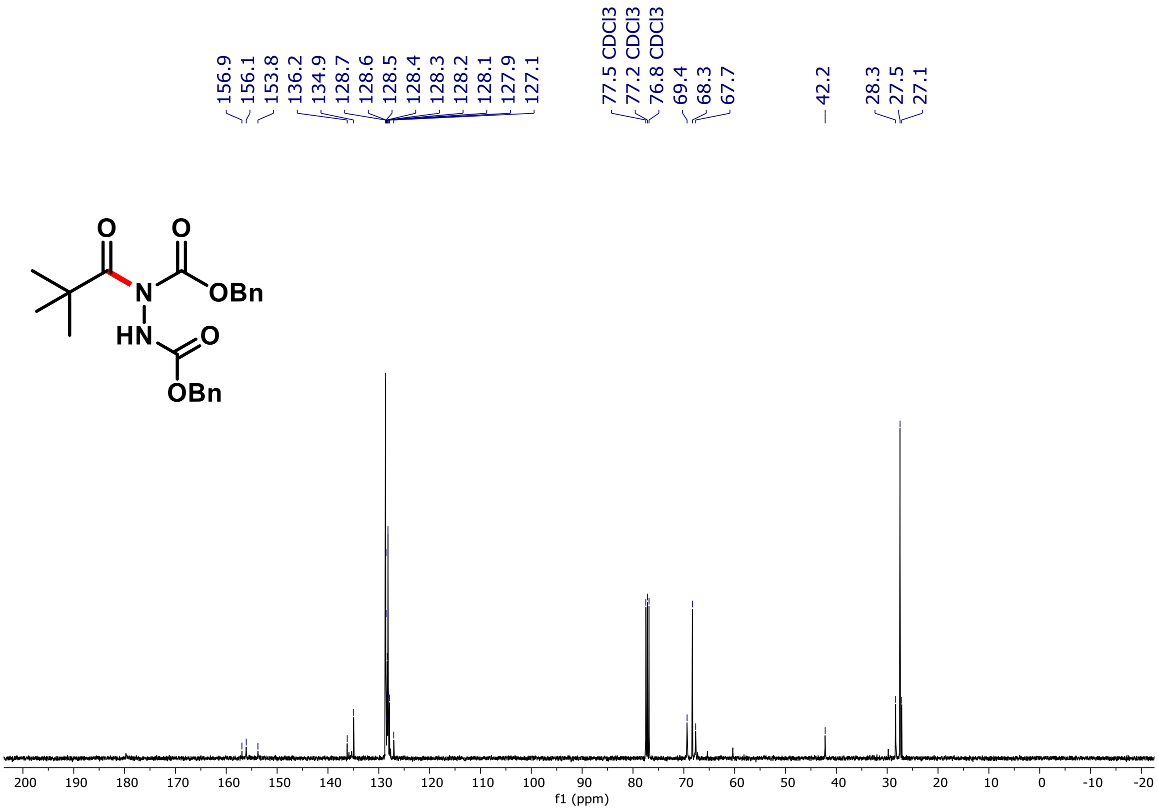

Figure S20. ^{13}C NMR of compound **3d** (101 MHz, $CDCl_3$)

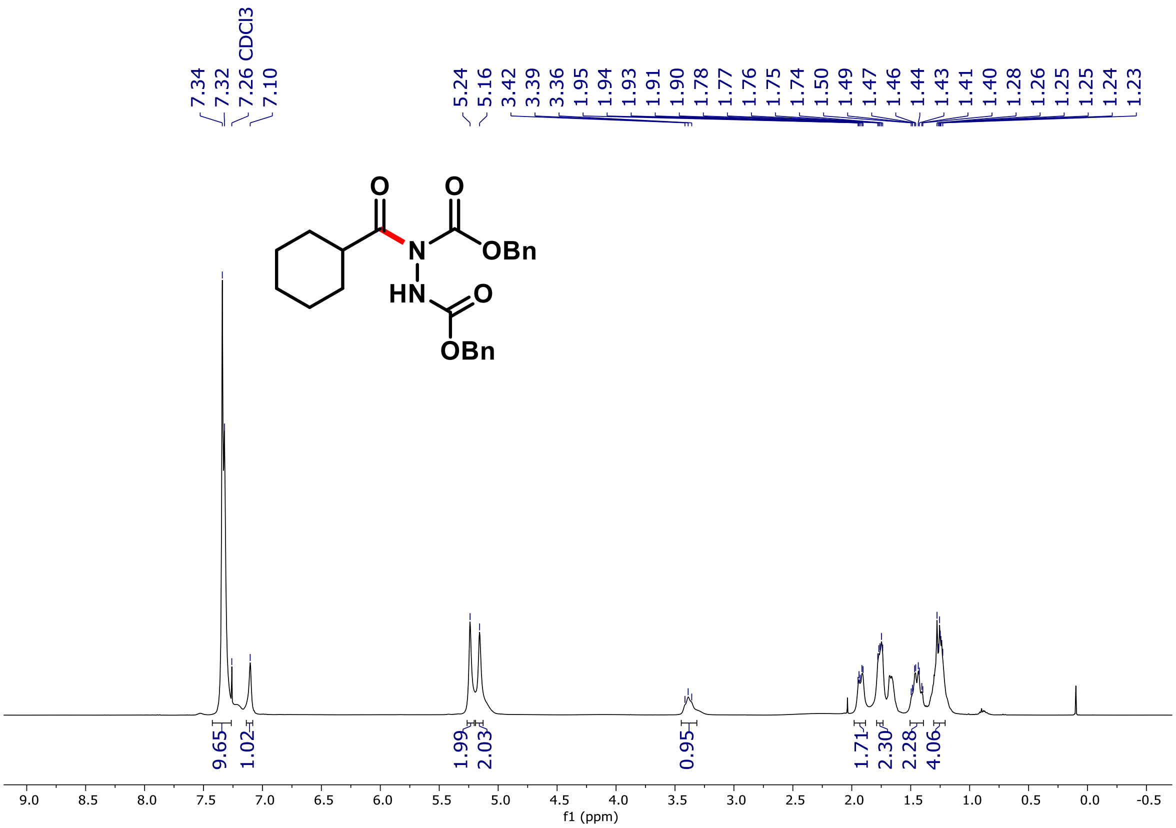

Figure S21. ^{1}H NMR of compound **3e** (400 MHz, $CDCl_3$)

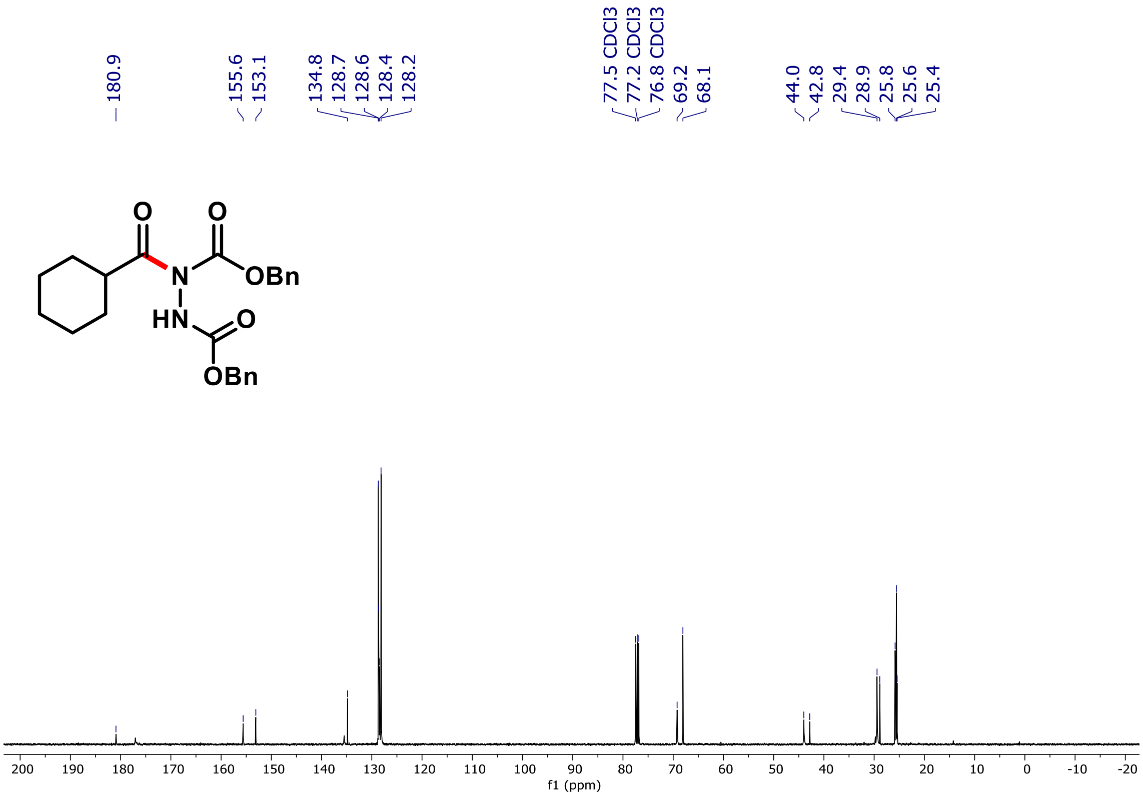

Figure S22. ^{13}C NMR of compound **3e** (101 MHz, $CDCl_3$)

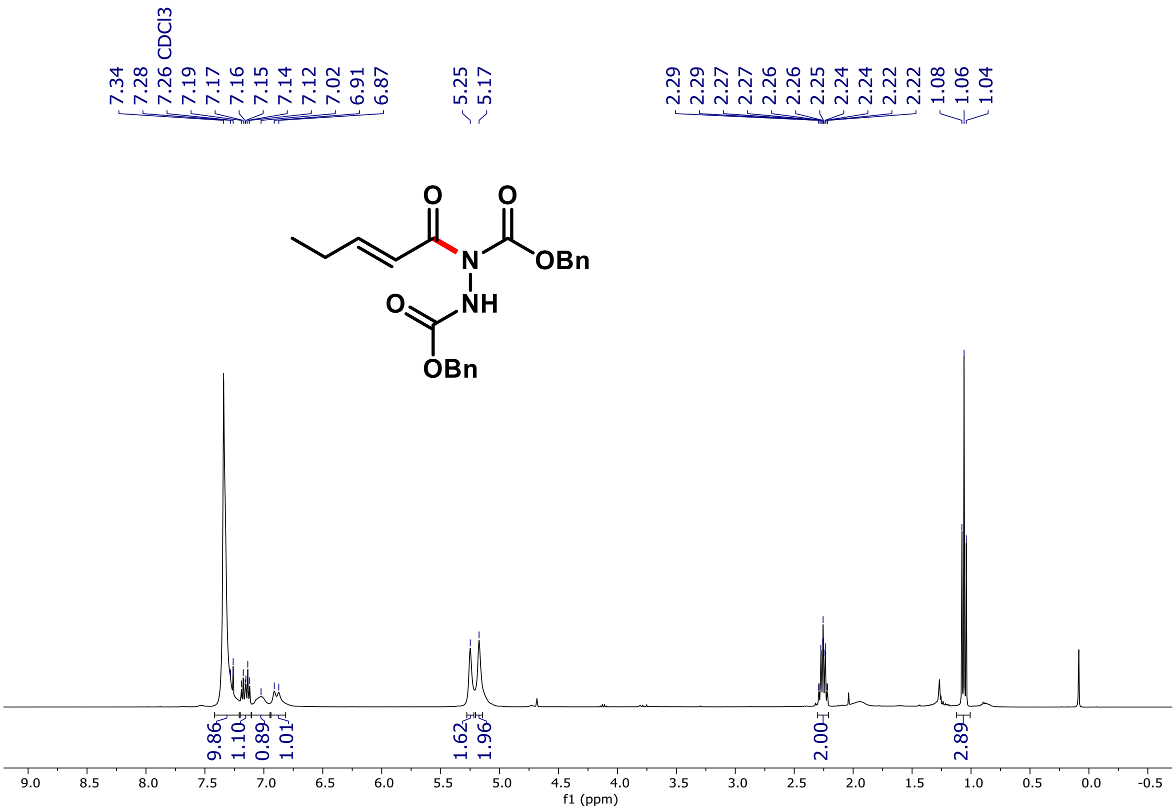

Figure S23. ^{1}H NMR of compound **3f** (400 MHz, $CDCl_3$)

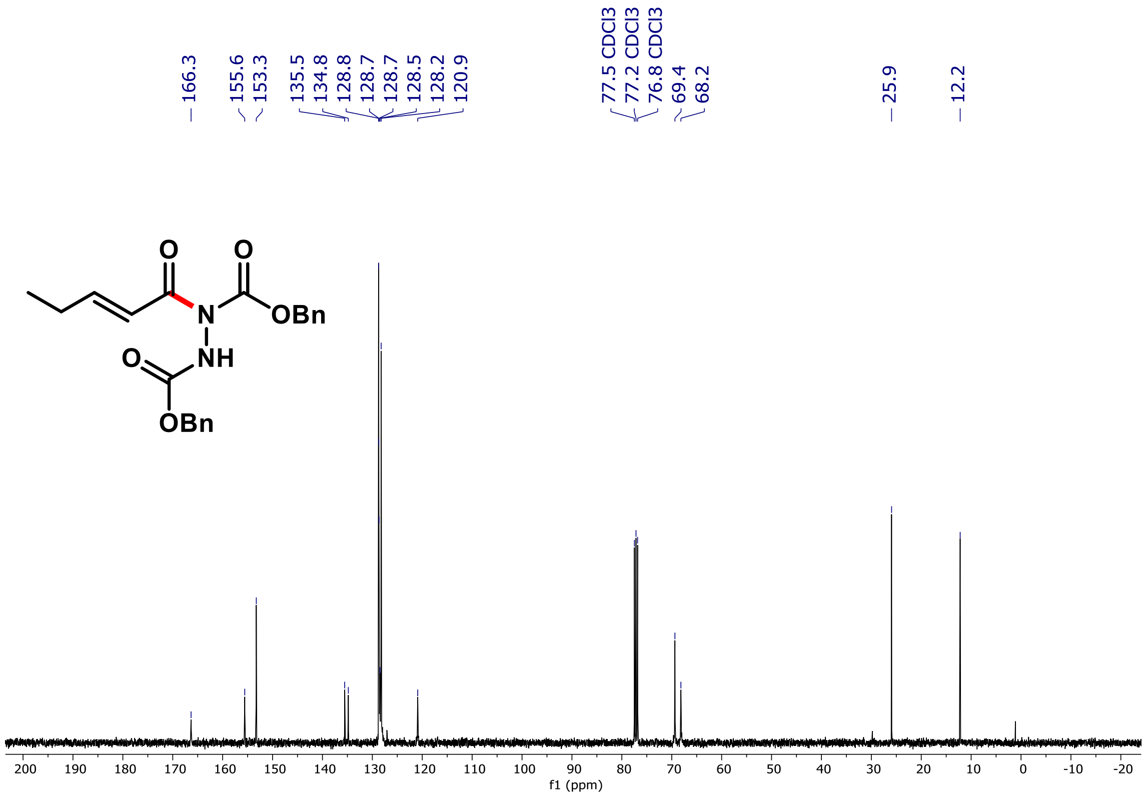

Figure S24. ^{13}C NMR of compound **3f** (101 MHz, $CDCl_3$)

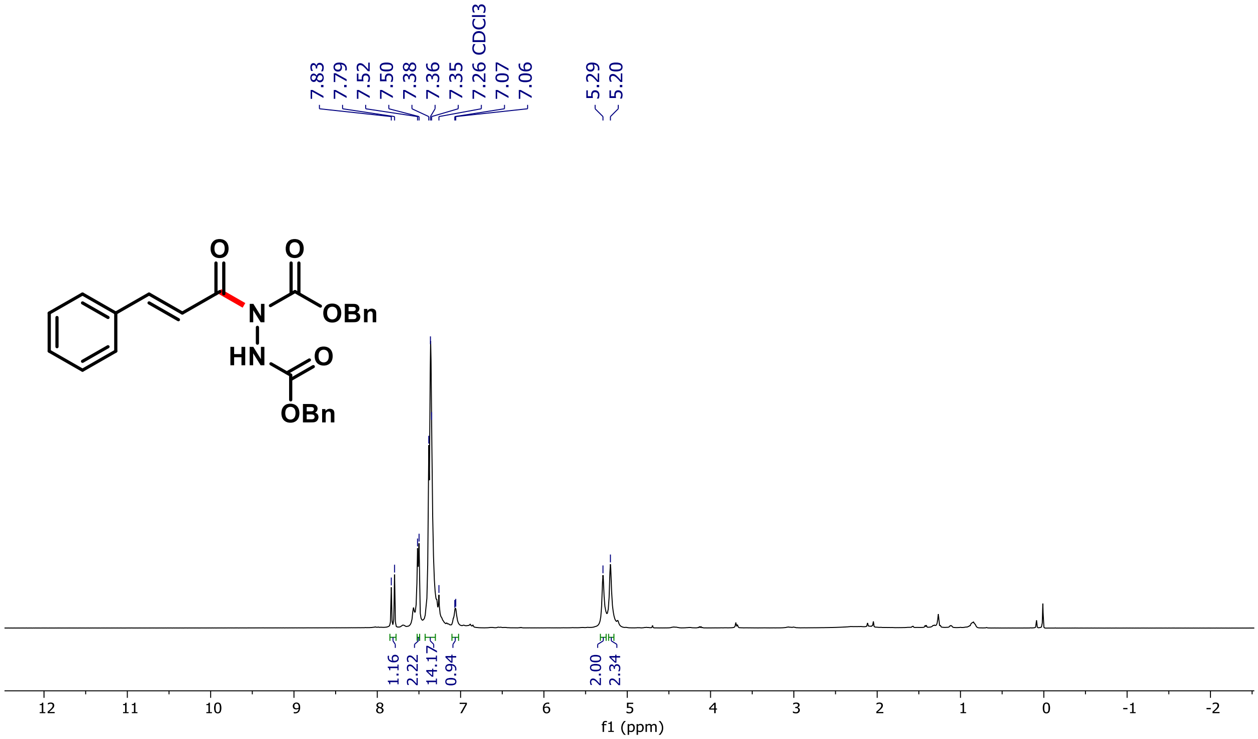

Figure S25. ^{1}H NMR of compound **3g** (400 MHz, $CDCl_3$).

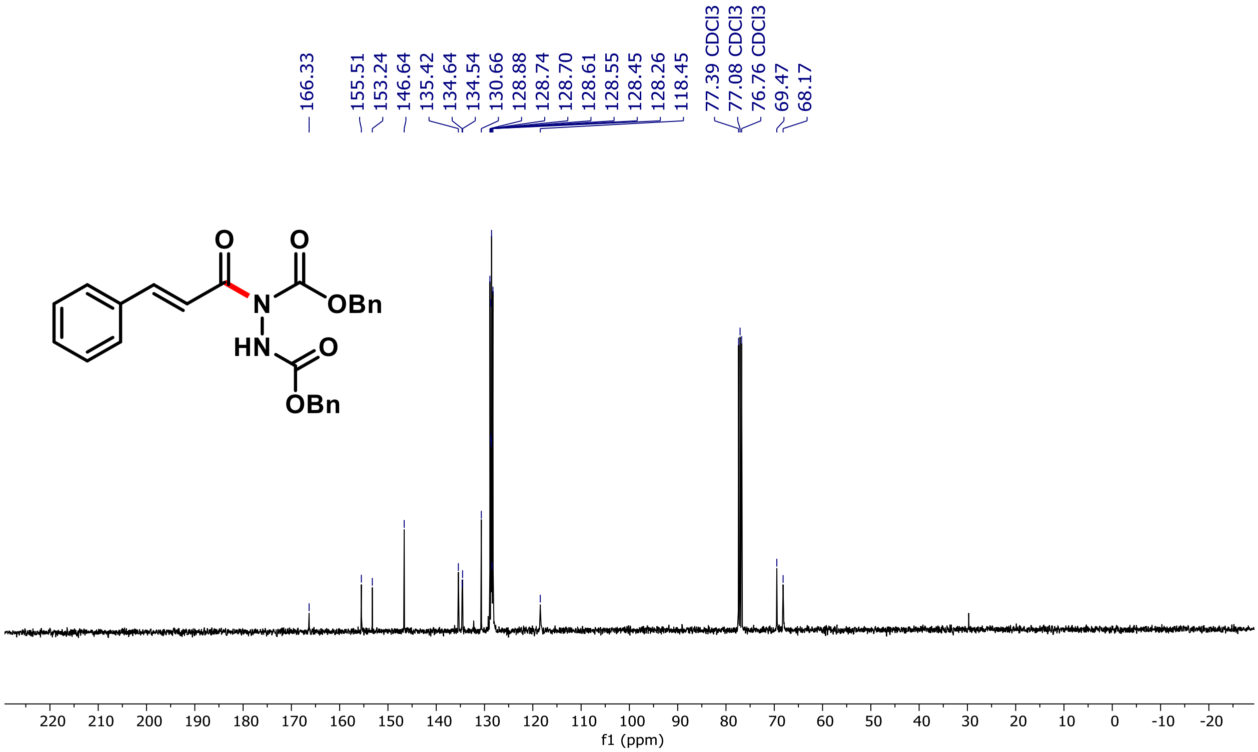

Figure S26. ^{13}C NMR of compound **3g** (101 MHz, $CDCl_3$).

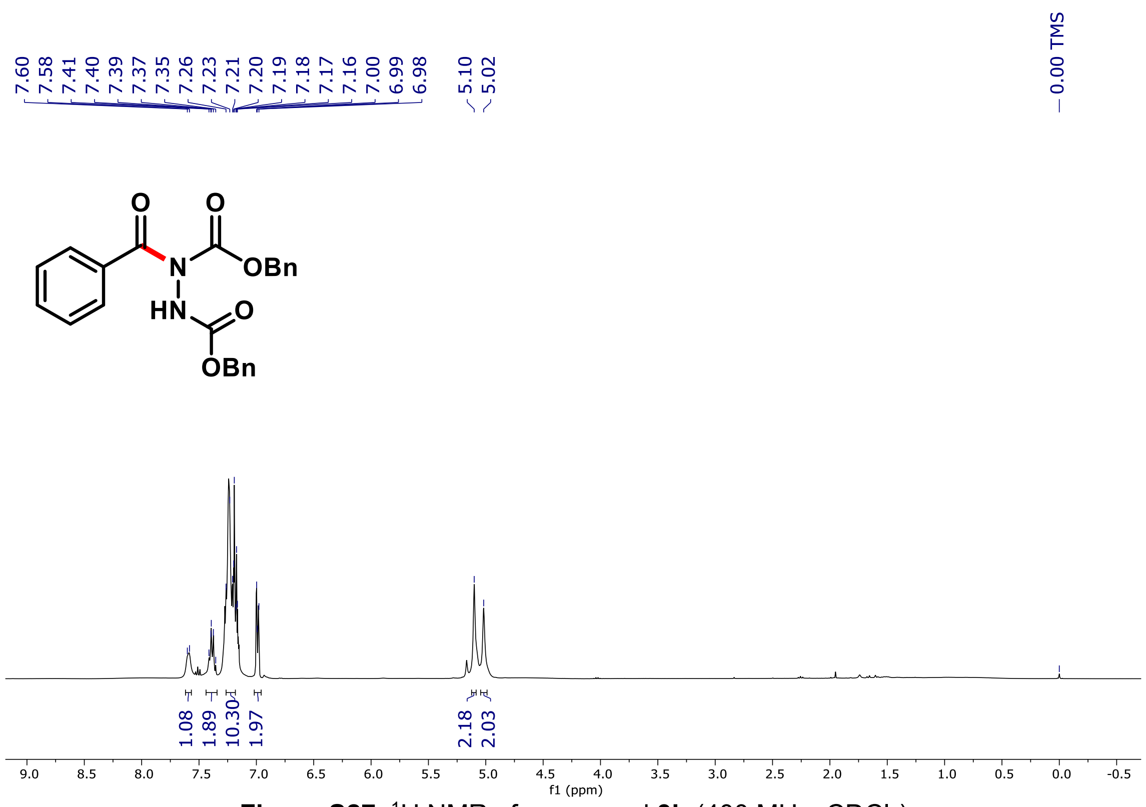

Figure S27. ^{1}H NMR of compound **3h** (400 MHz, $CDCl_3$)

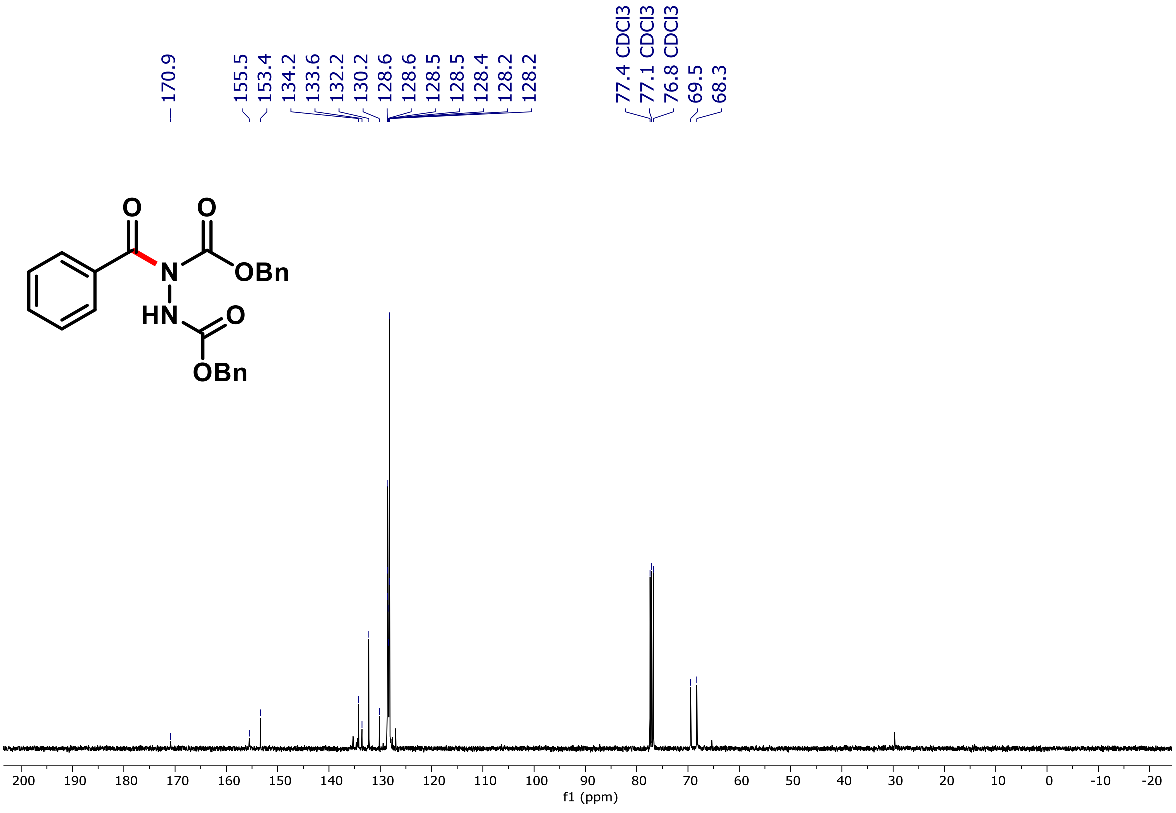

Figure S28. ^{13}C NMR of compound **3h** (101 MHz, $CDCl_3$)

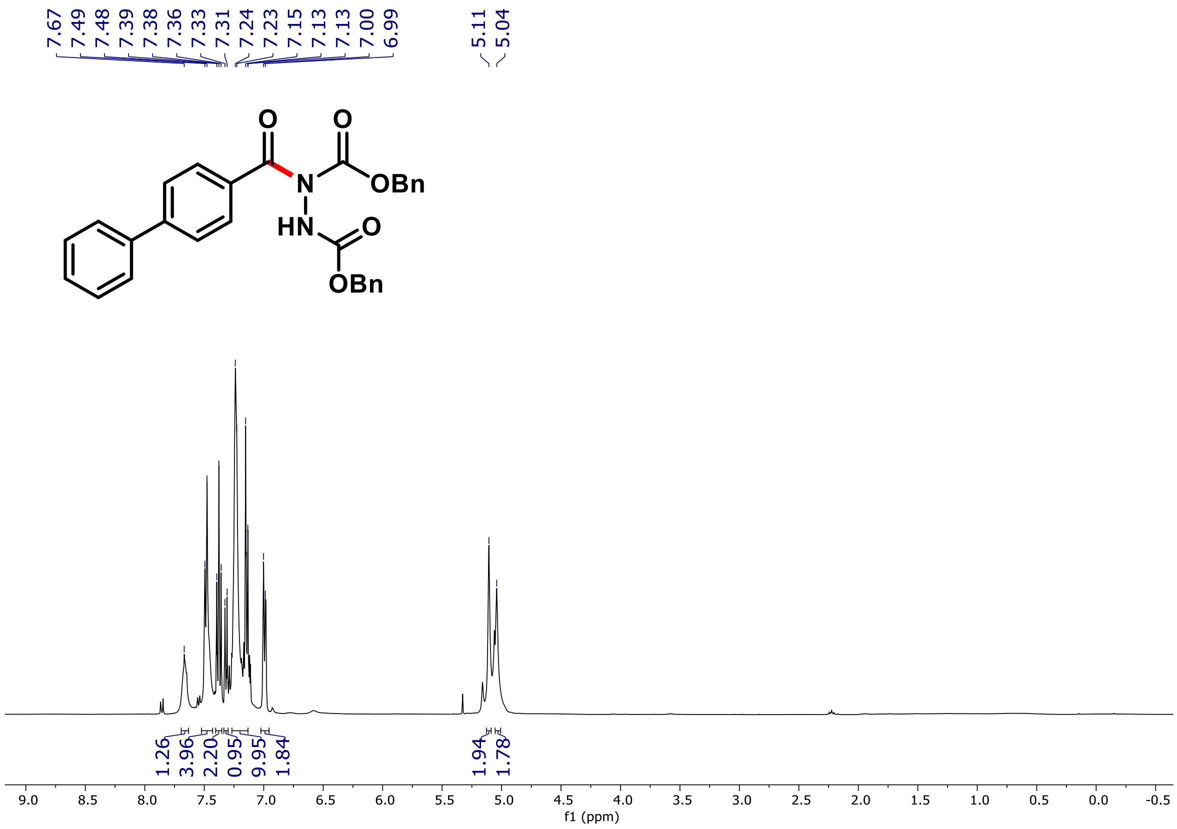

Figure S29. ^{1}H NMR of compound **3i** (400 MHz, $CDCl_3$)

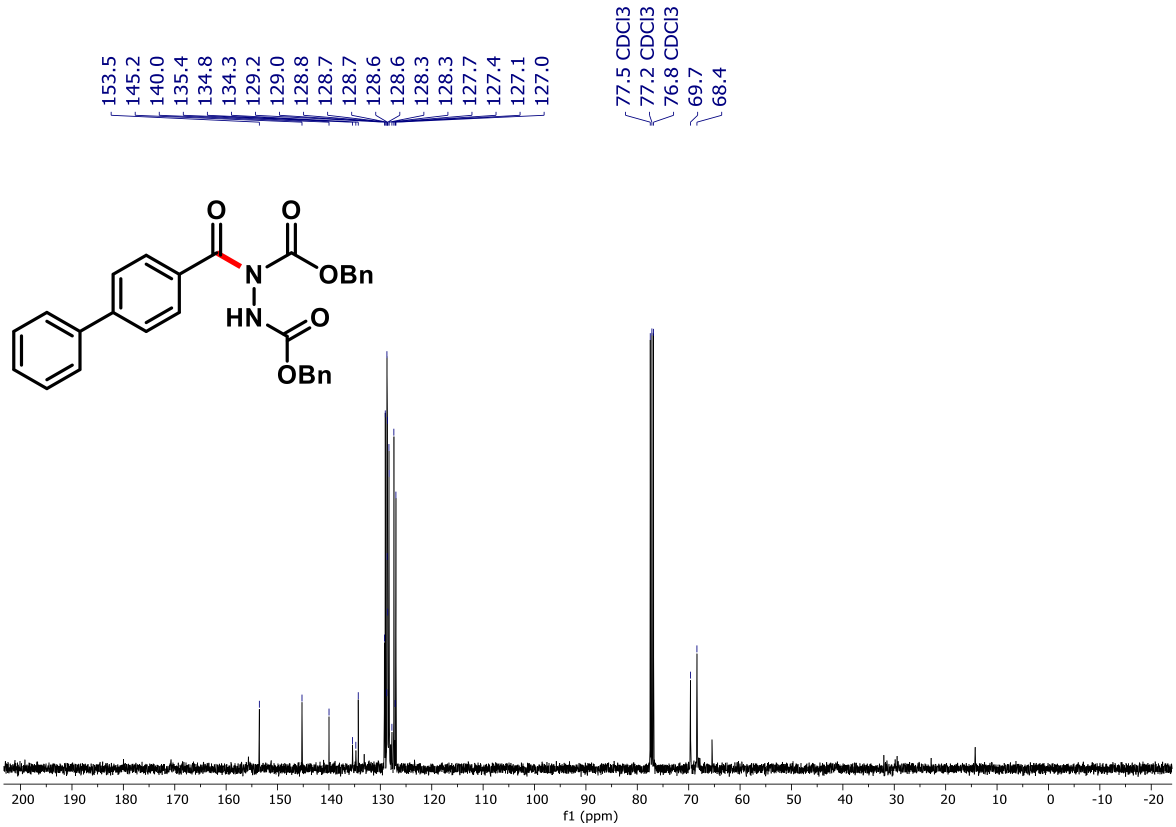

Figure S30. ^{13}C NMR of compound **3i** (101 MHz, $CDCl_3$)

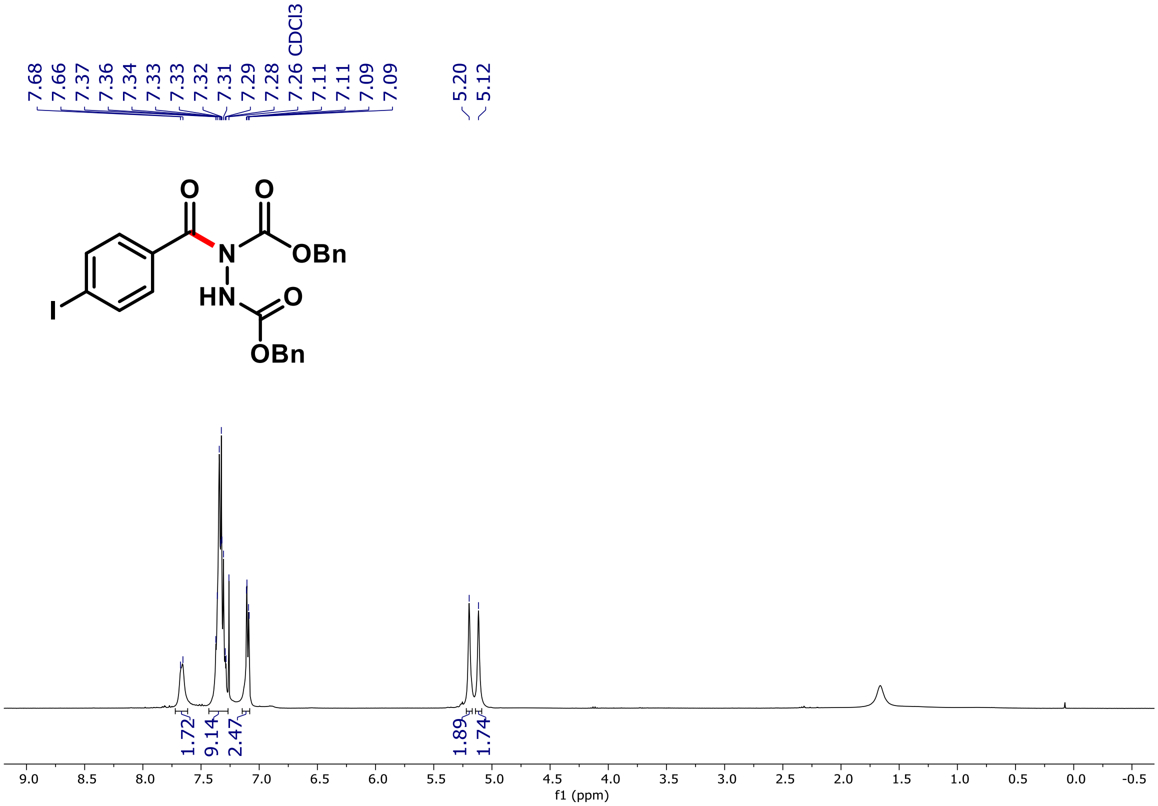

Figure S31. ^{1}H NMR of compound **3j** (400 MHz, $CDCl_3$)

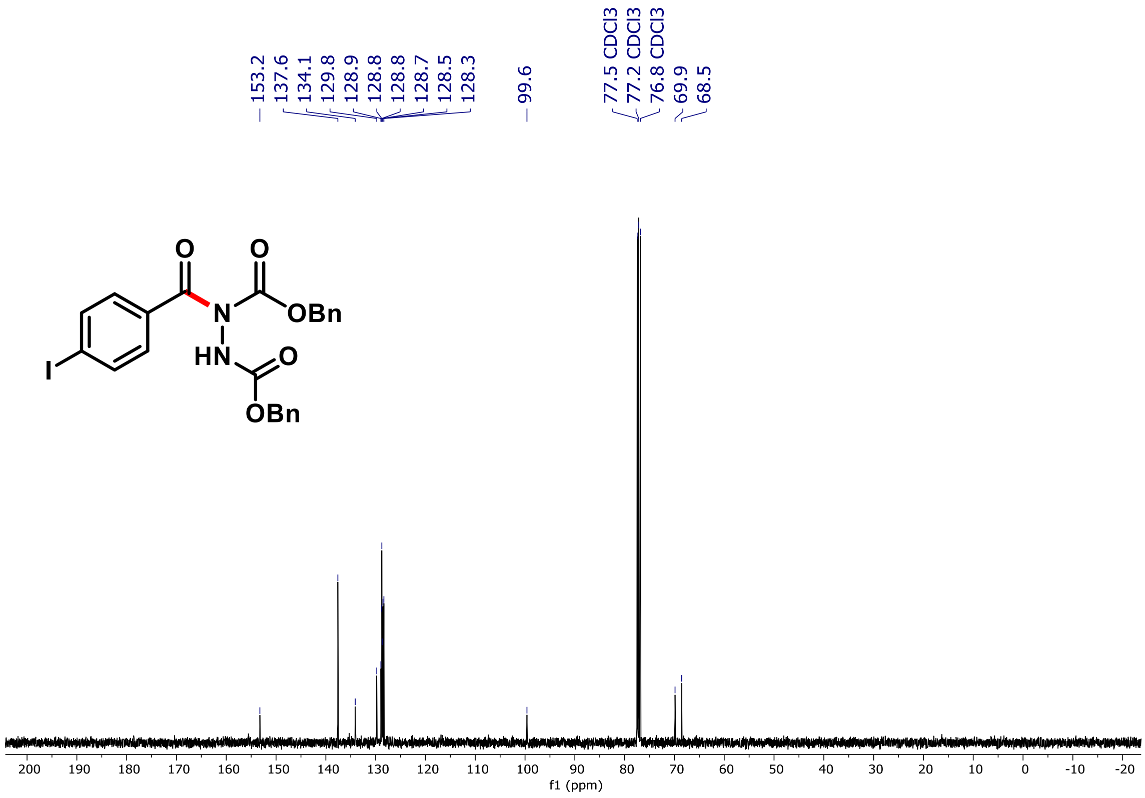

Figure S32. ^{13}C NMR of compound **3j** (101 MHz, $CDCl_3$)

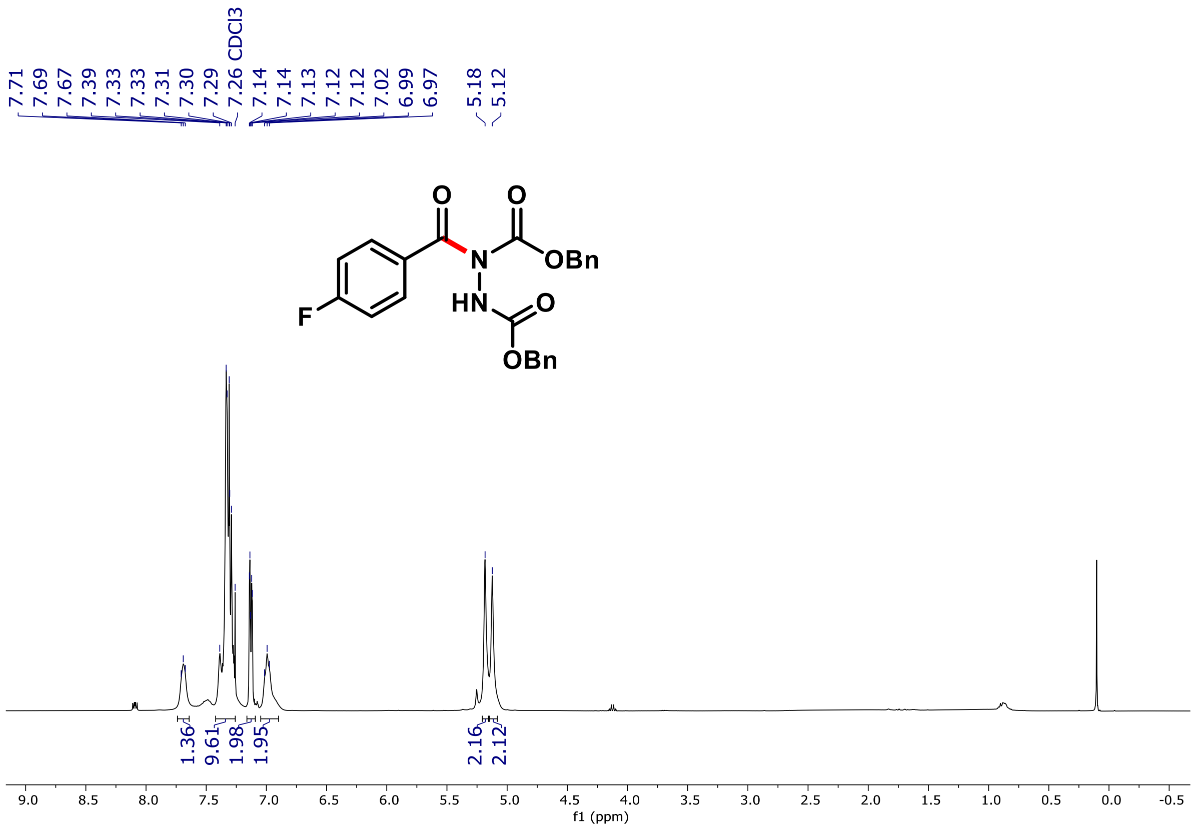

Figure S33. ^{1}H NMR of compound **3k** (400 MHz, $CDCl_3$)

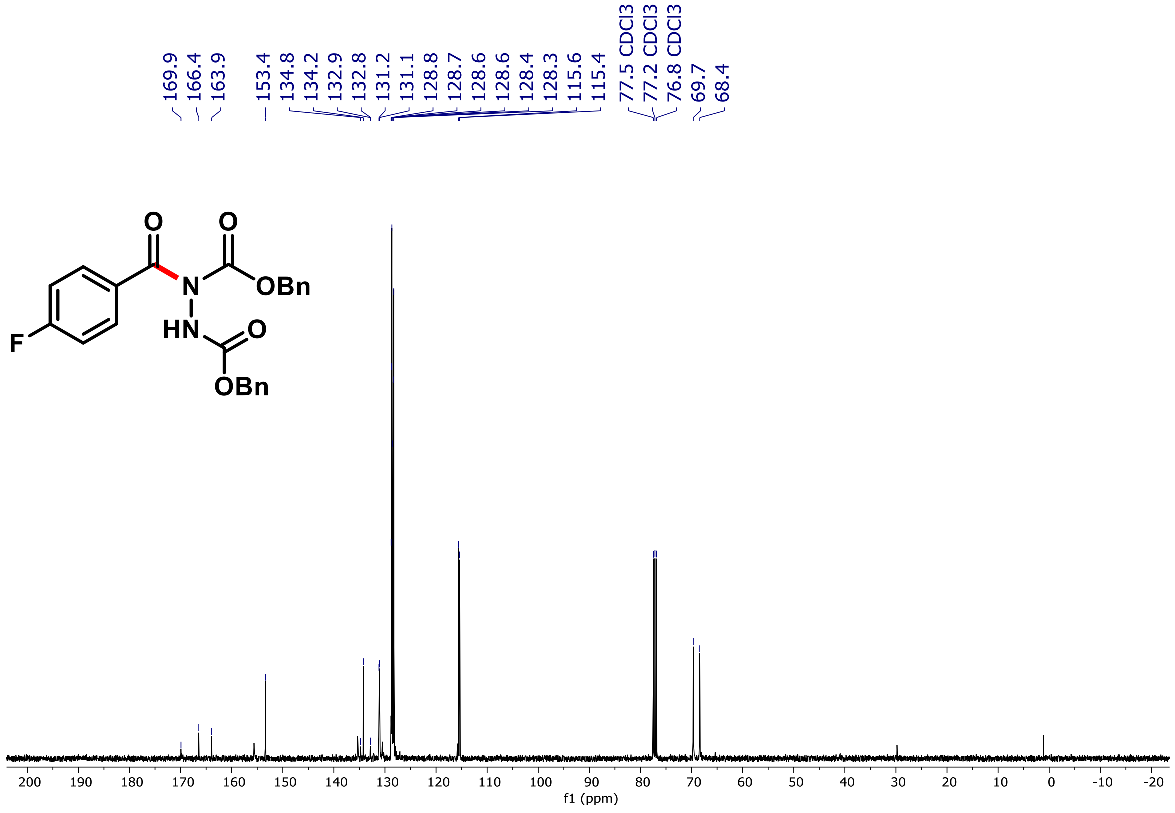

Figure S34. ^{13}C NMR of compound **3k** (101 MHz, $CDCl_3$).

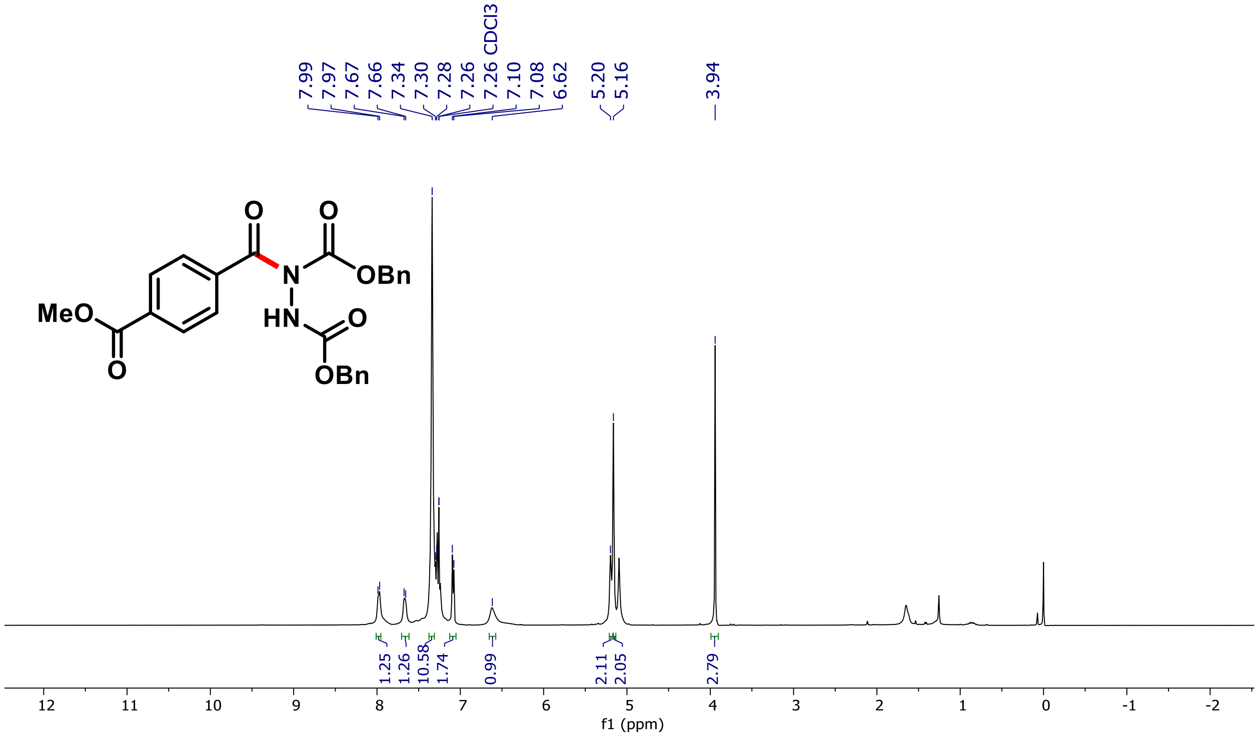

Figure S35. ^{1}H NMR of compound **3l** (400 MHz, $CDCl_3$).

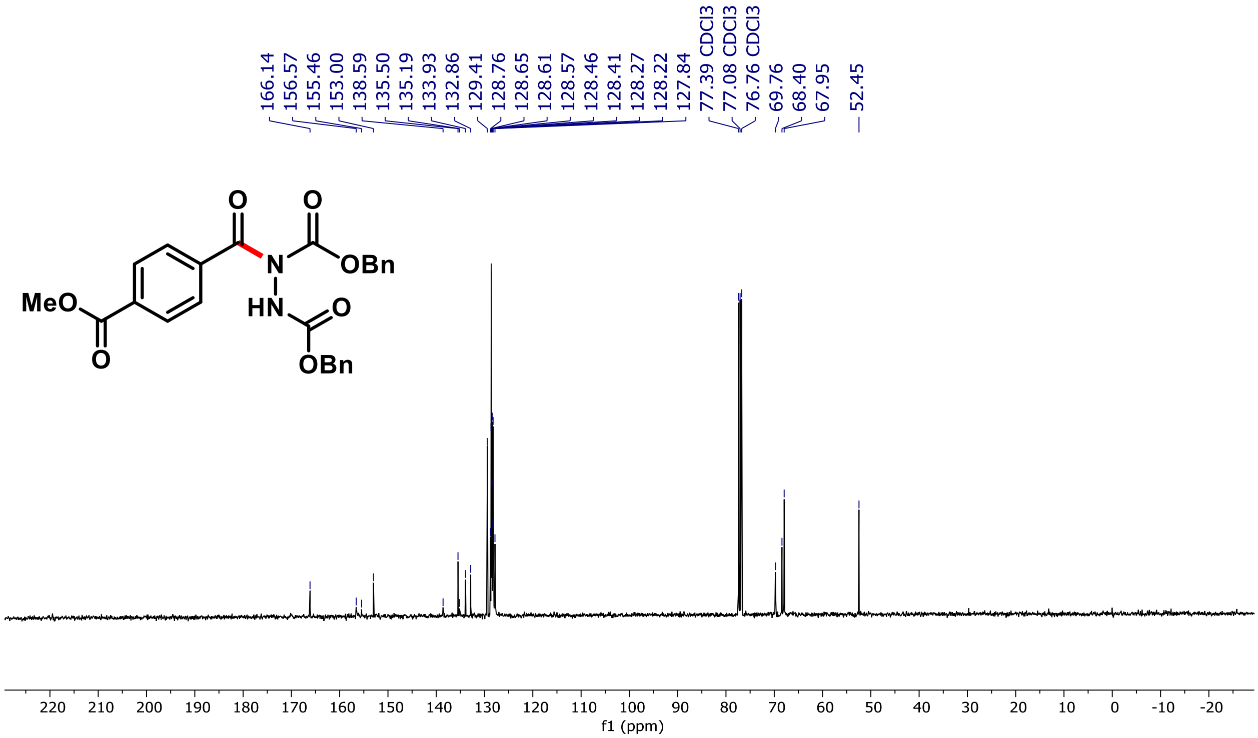

Figure S36. ^{13}C NMR of compound **3l** (101 MHz, $CDCl_3$).

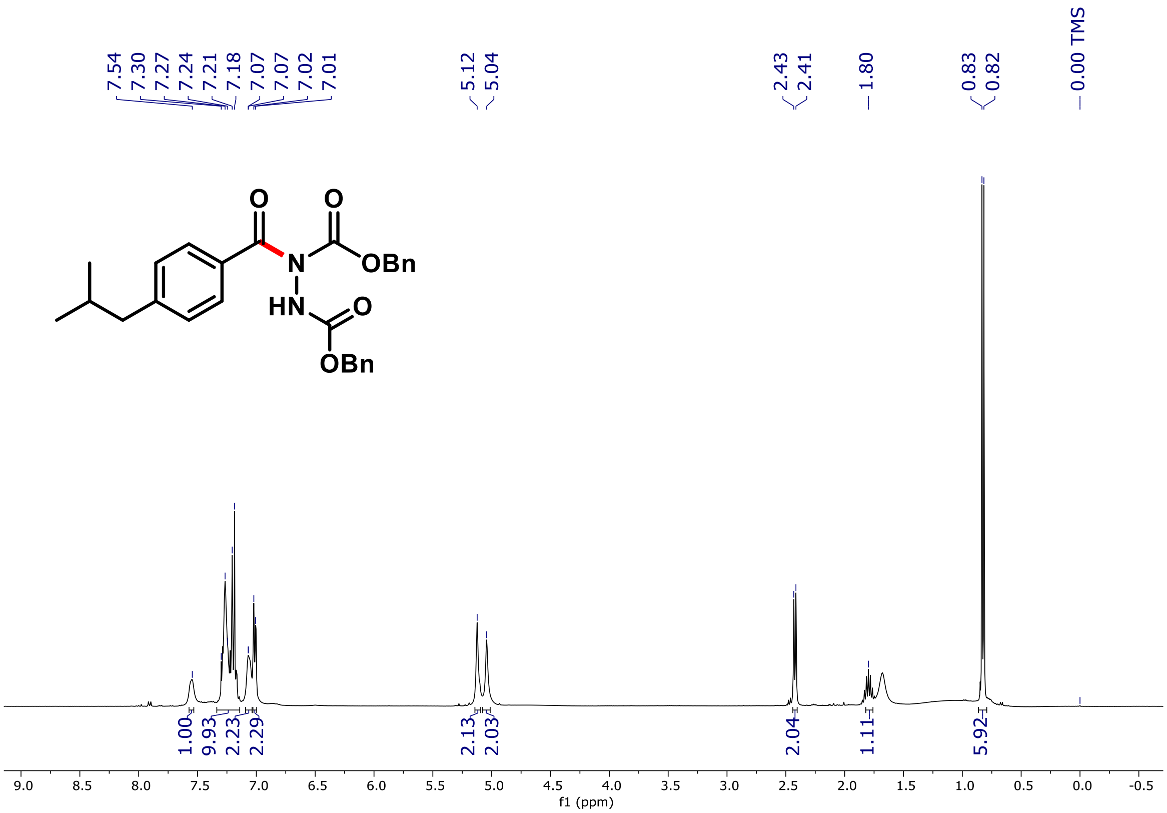

Figure S37. ^{1}H NMR of compound **3m** (400 MHz, $CDCl_3$)

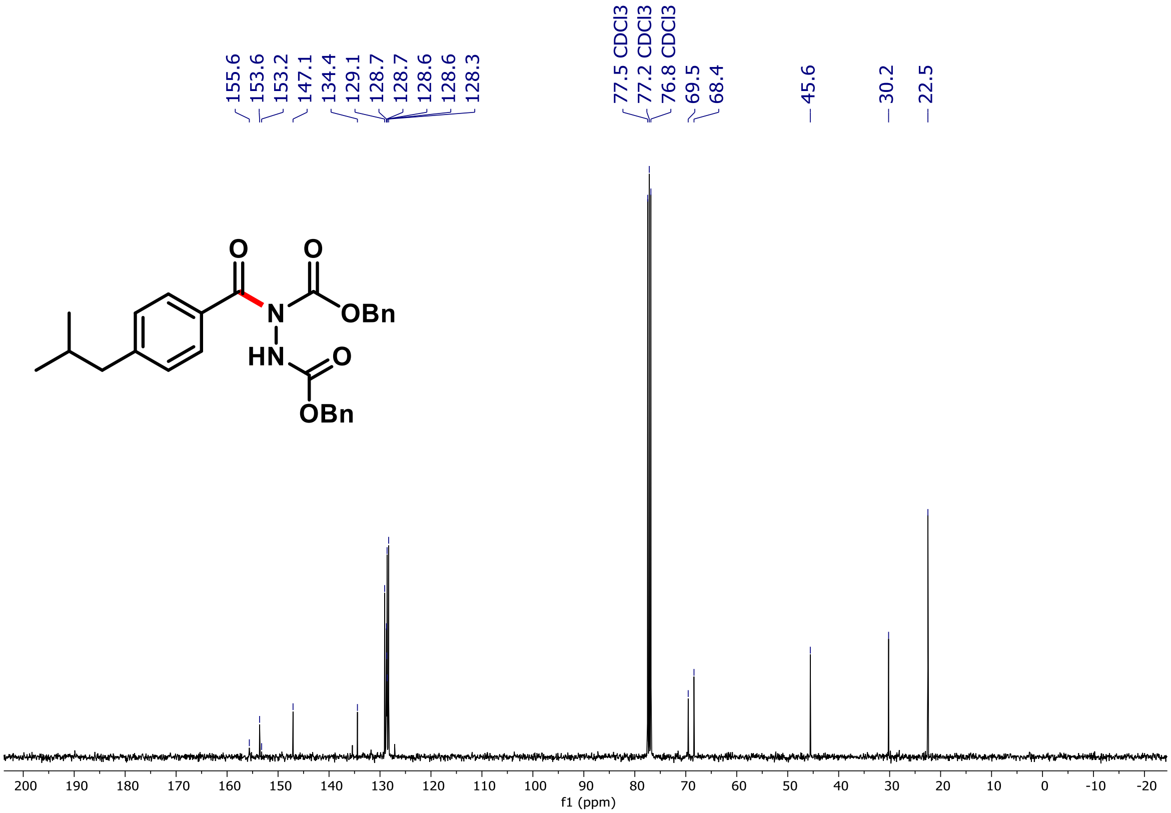

Figure S38. ^{13}C NMR of compound **3m** (101 MHz, $CDCl_3$).

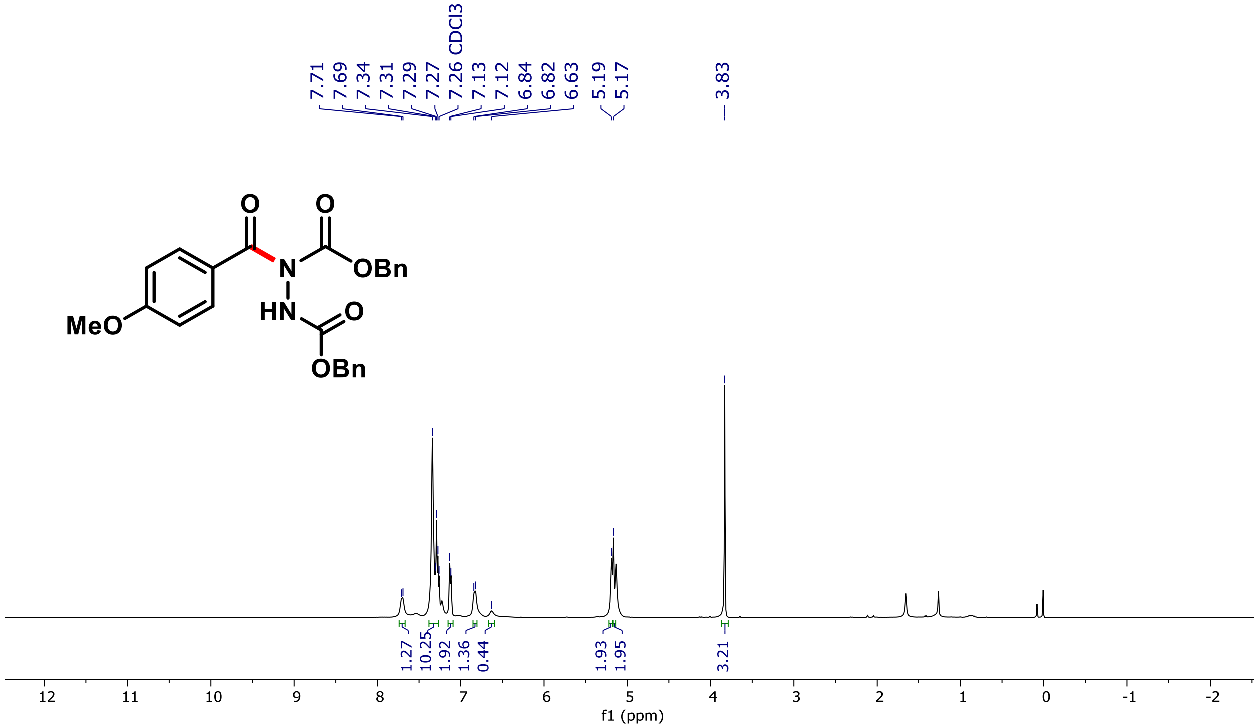

Figure S39. ^{1}H NMR of compound **3n** (400 MHz, $CDCl_3$).

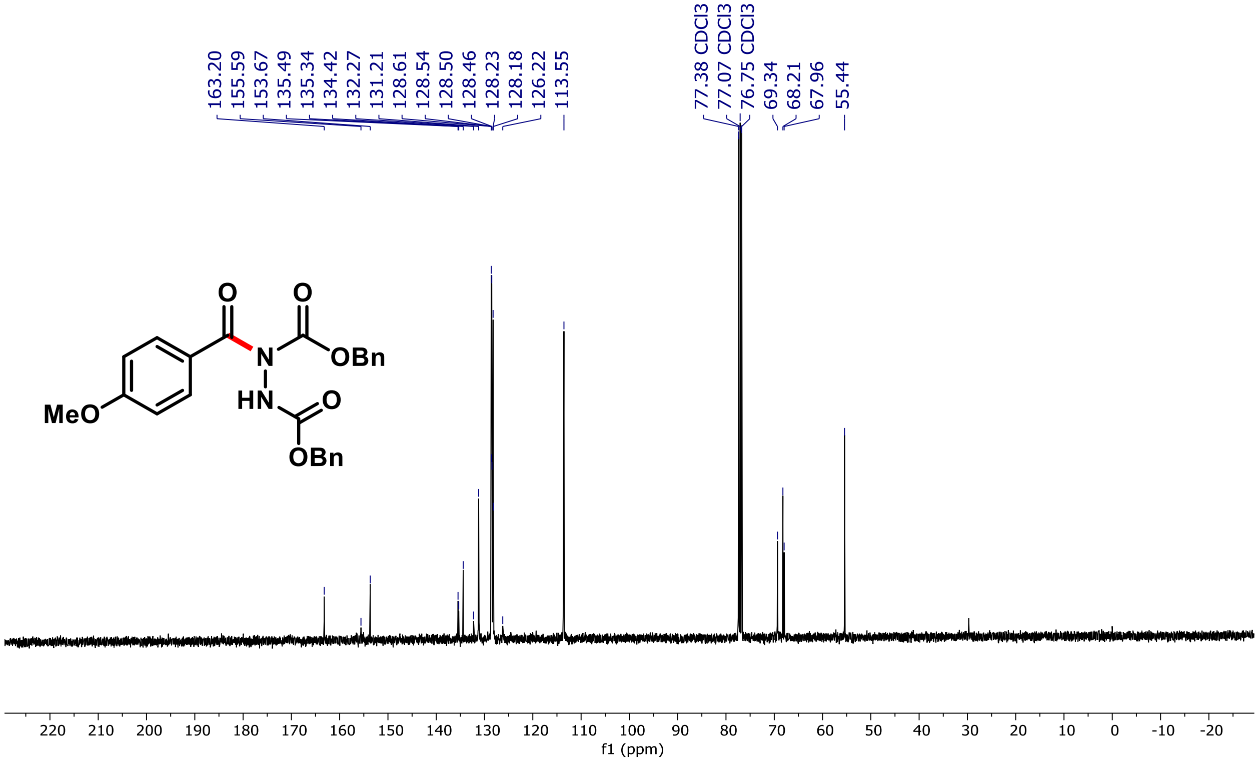

Figure S40. ^{13}C NMR of compound **3n** (101 MHz, $CDCl_3$).

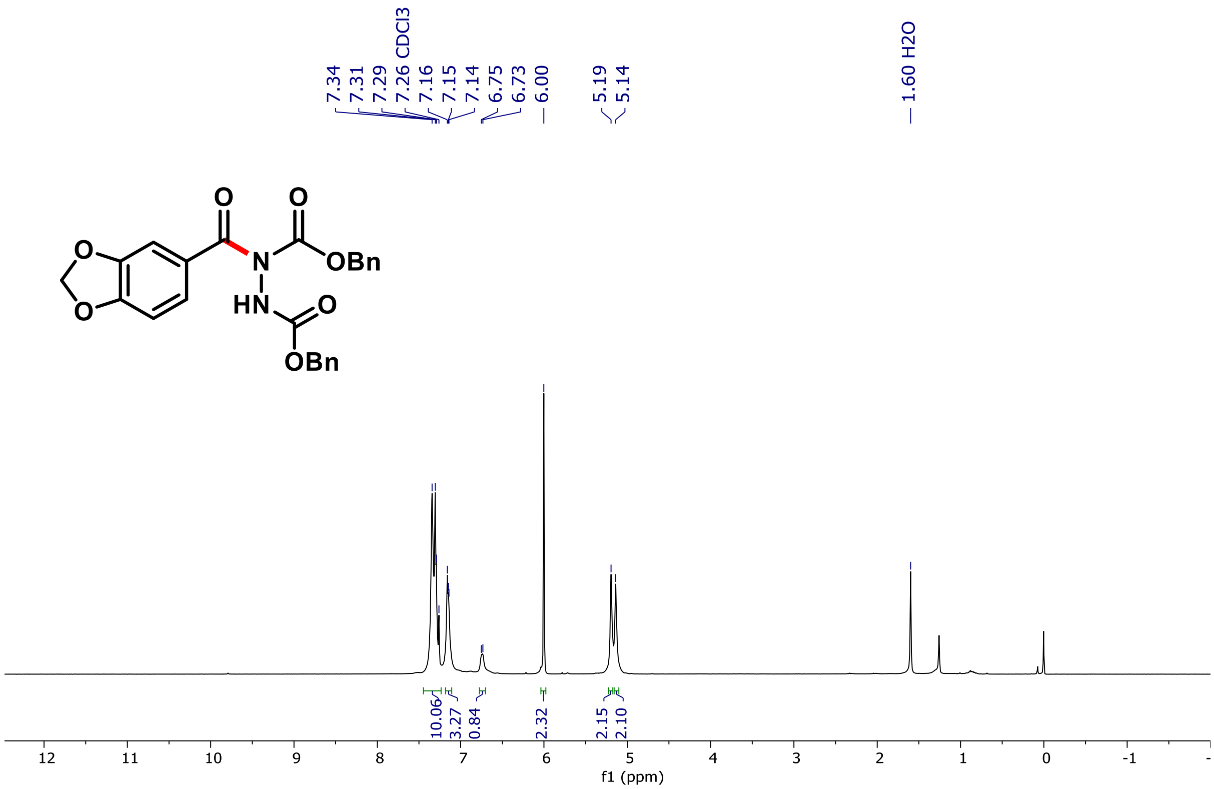

Figure S41. ^{1}H NMR of compound **3o** (400 MHz, $CDCl_3$)

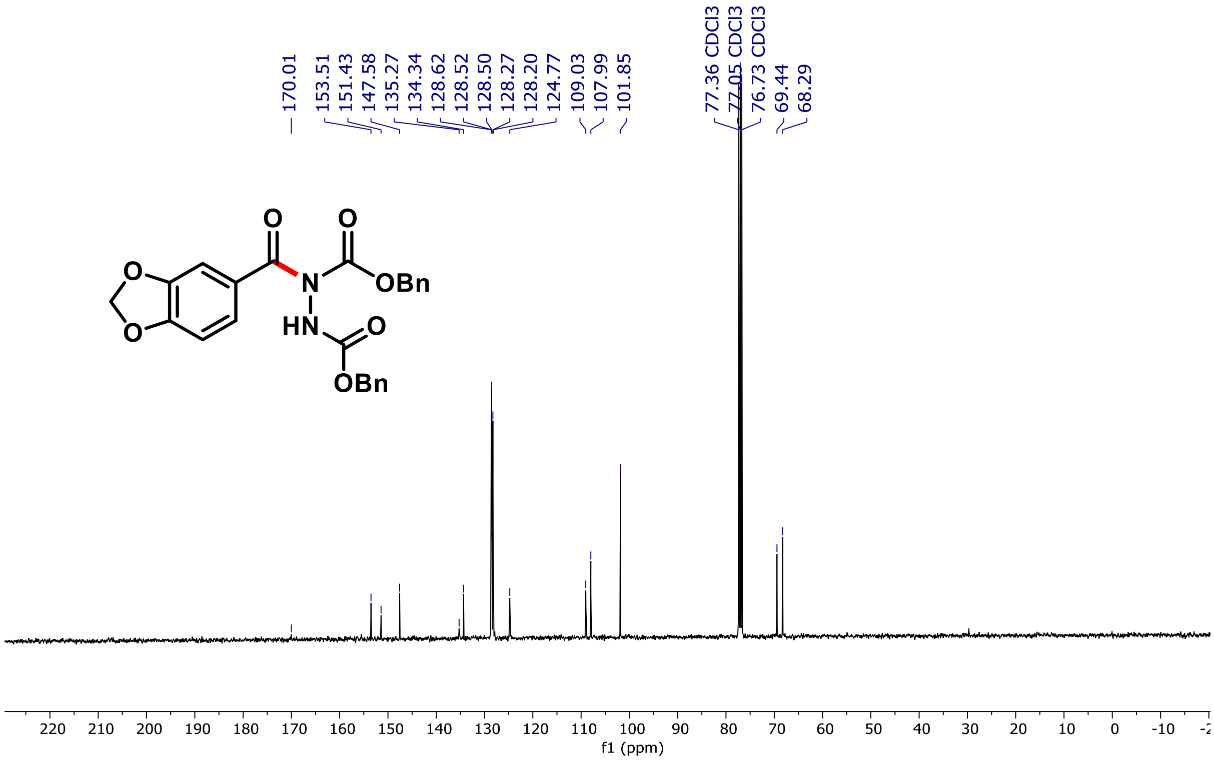

Figure S42. ^{13}C NMR of compound **3o** (101 MHz, $CDCl_3$).

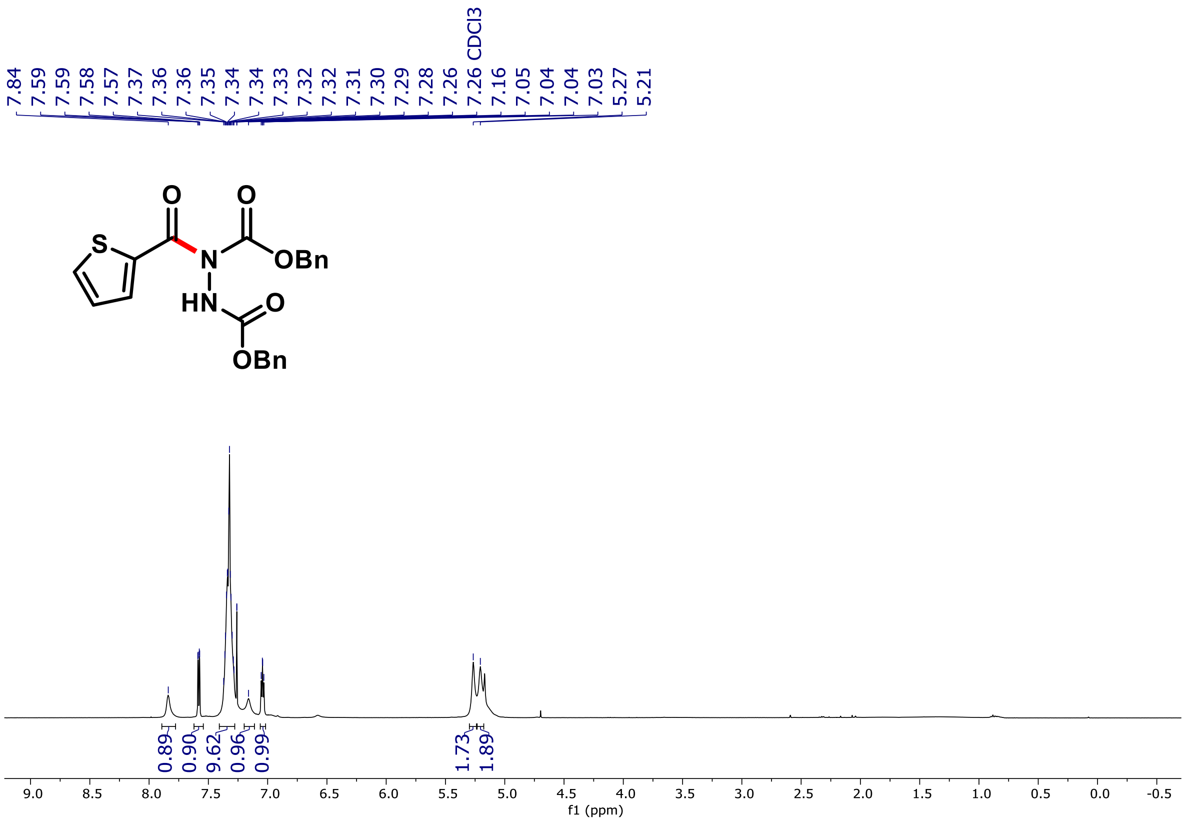

Figure S43. ^{1}H NMR of compound **3p** (400 MHz, $CDCl_3$)

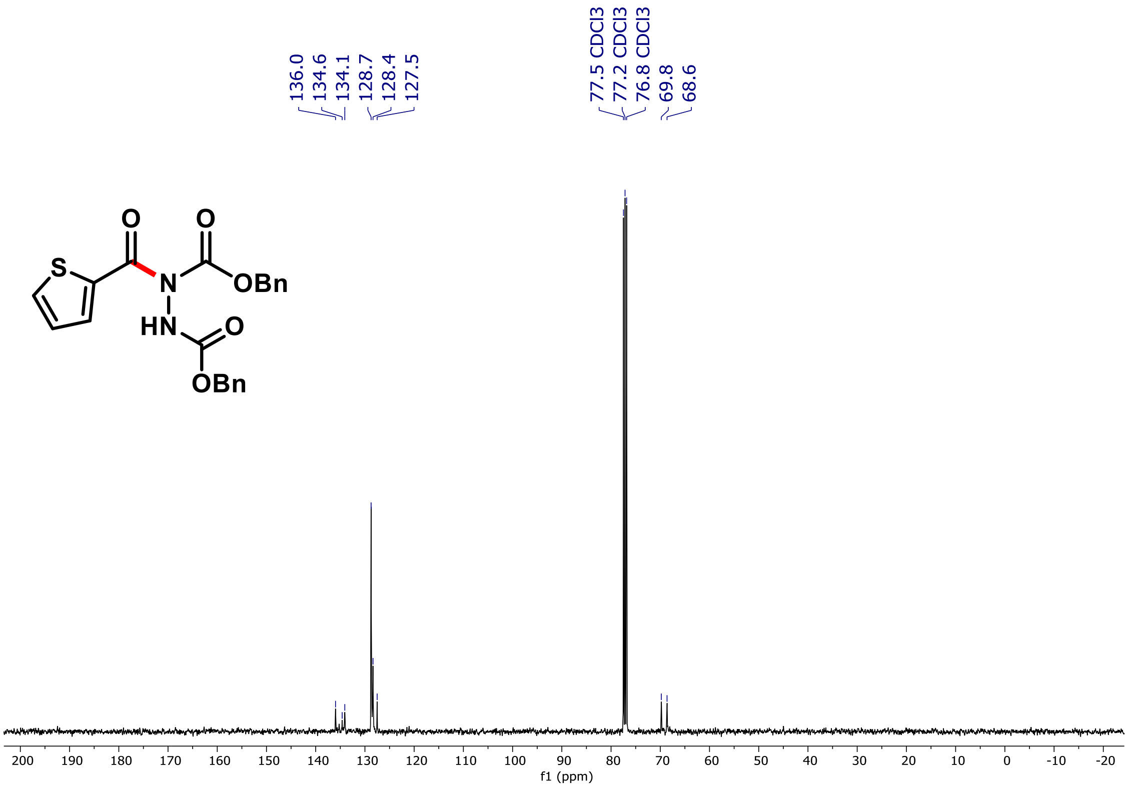

Figure S44. ^{13}C NMR of compound **3p** (101 MHz, $CDCl_3$).

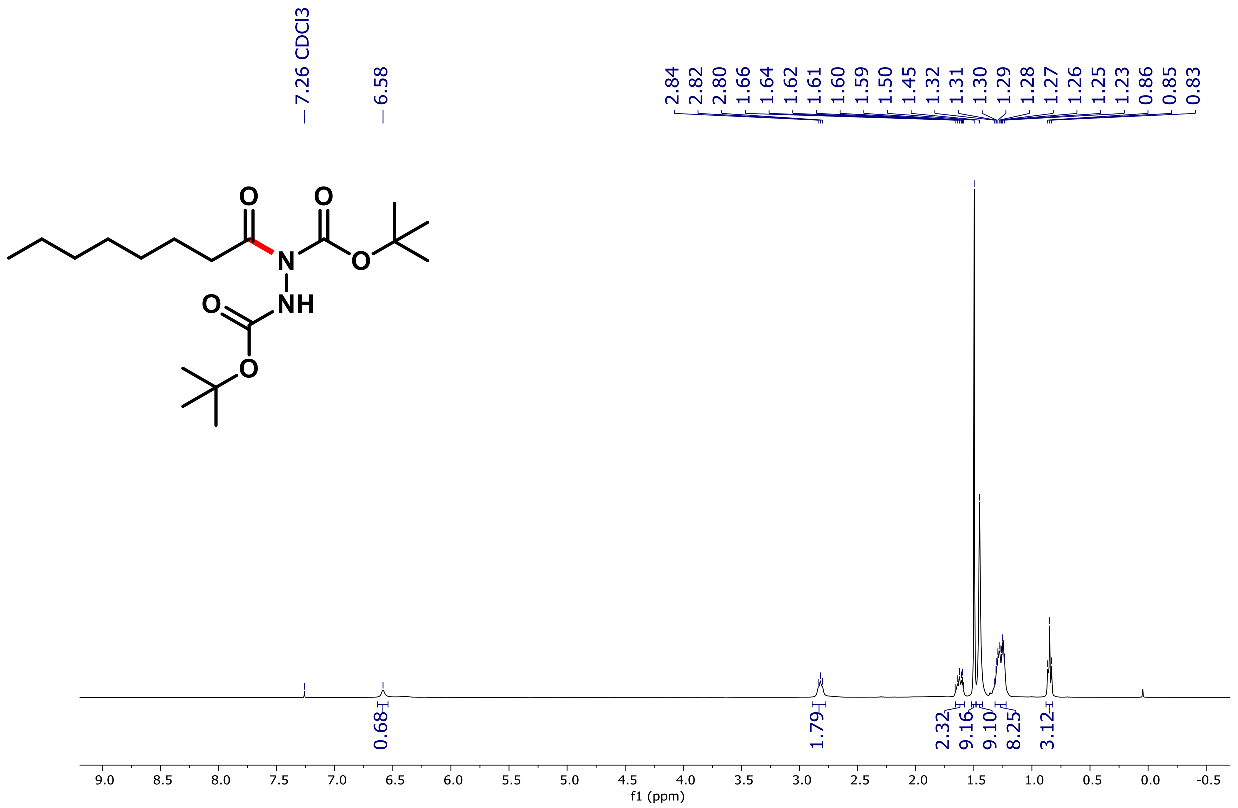

Figure S45. ^{1}H NMR of compound **3q** (400 MHz, $CDCl_3$)

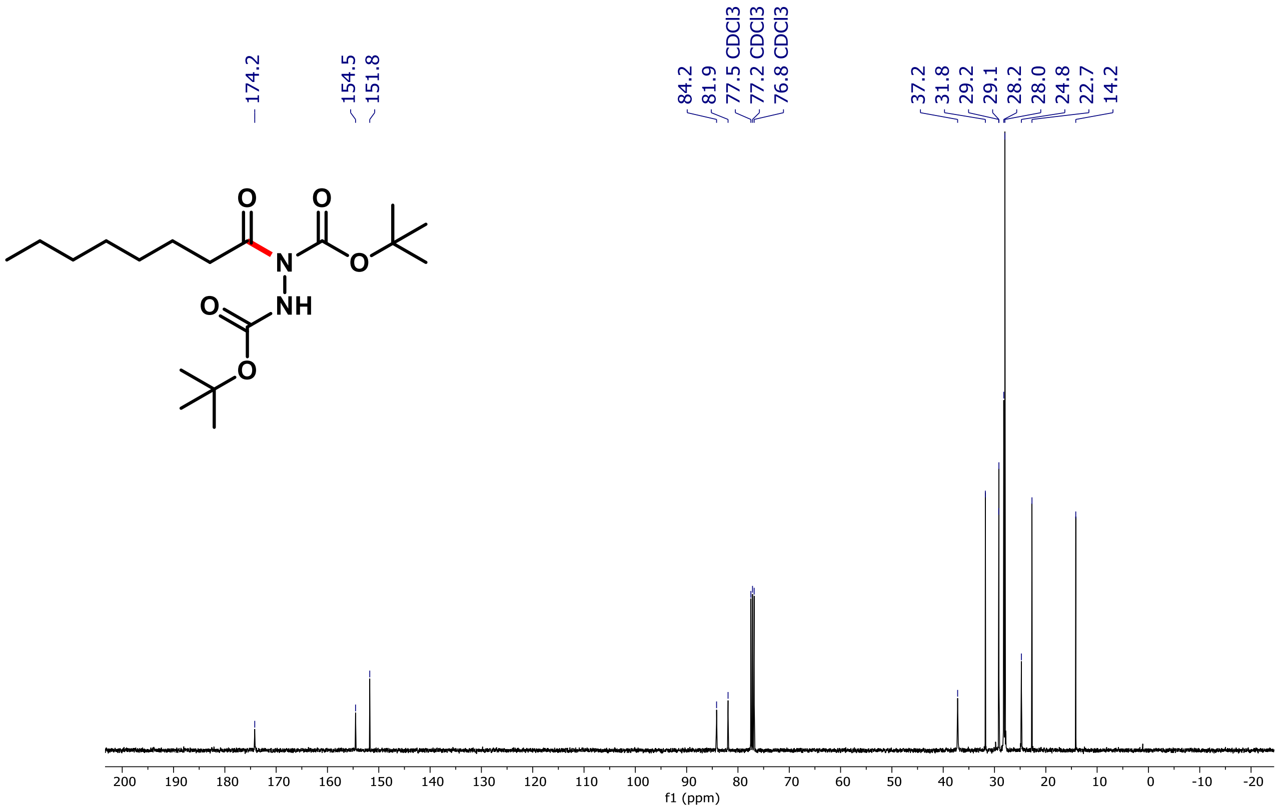

Figure S46. ^{13}C NMR of compound **3q** (101 MHz, $CDCl_3$).

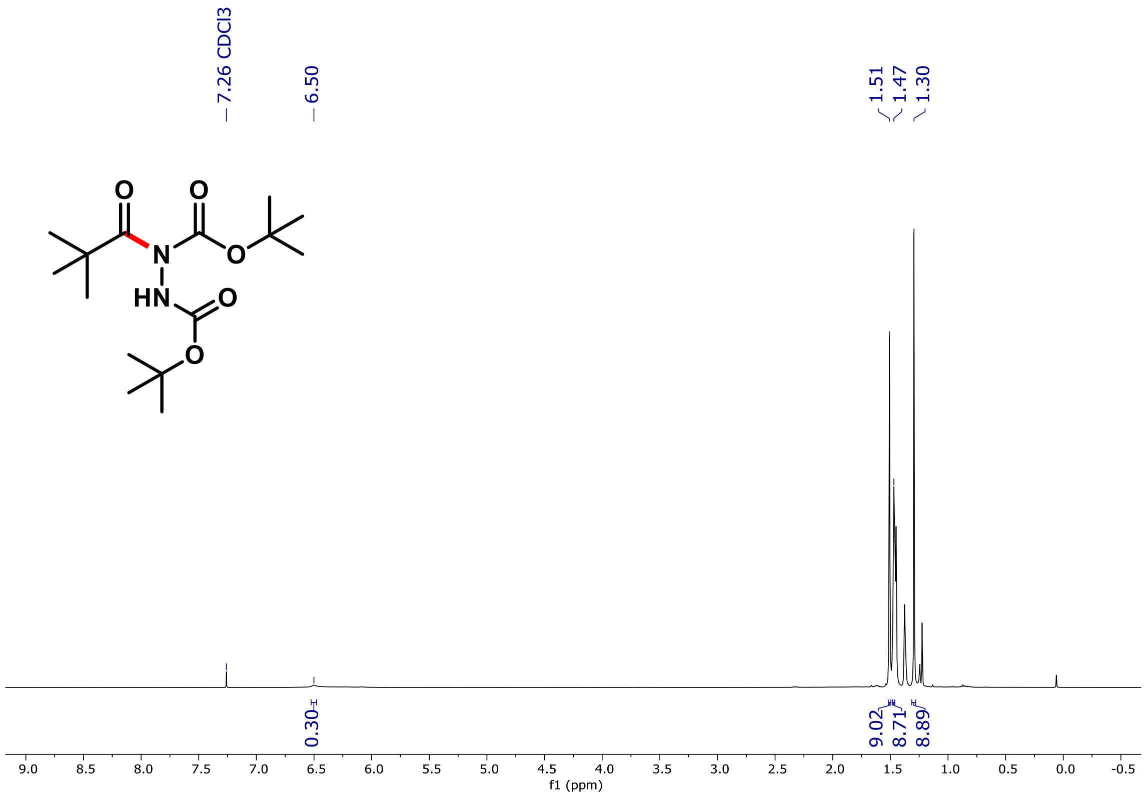

Figure S47. ^{1}H NMR of compound **3r** (400 MHz, $CDCl_3$)

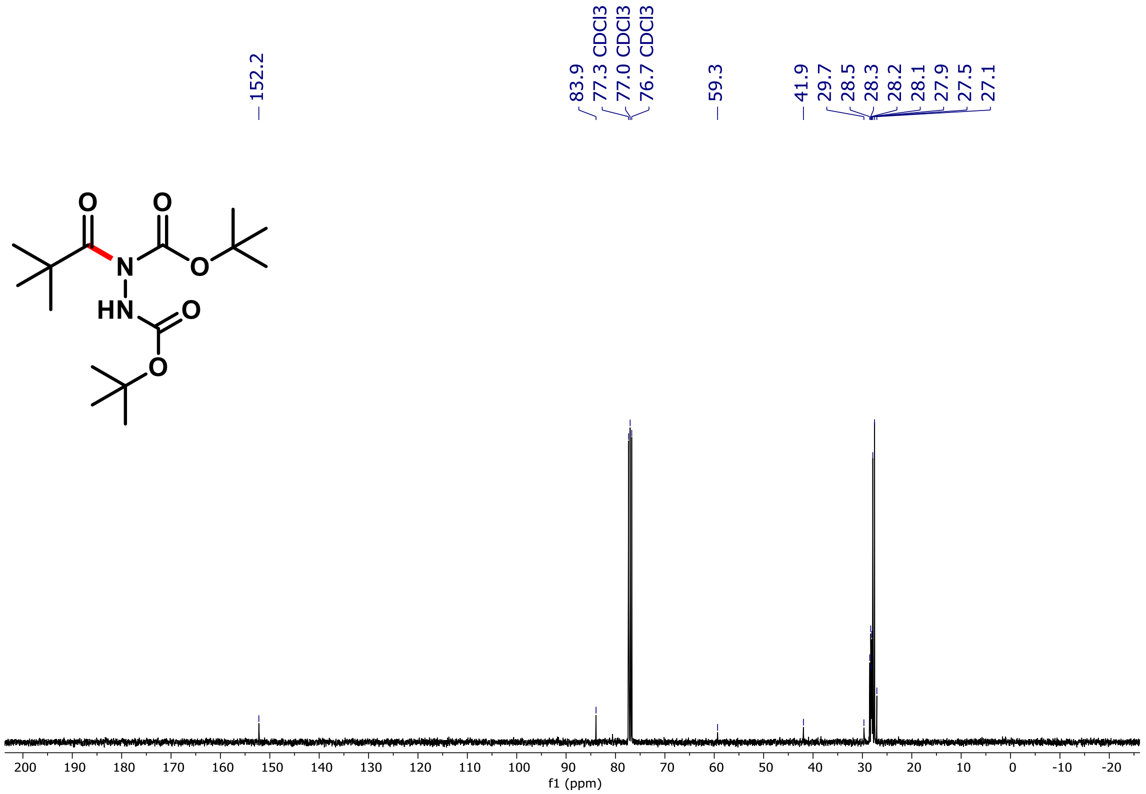

Figure S48. ^{13}C NMR of compound **3r** (101 MHz, $CDCl_3$)

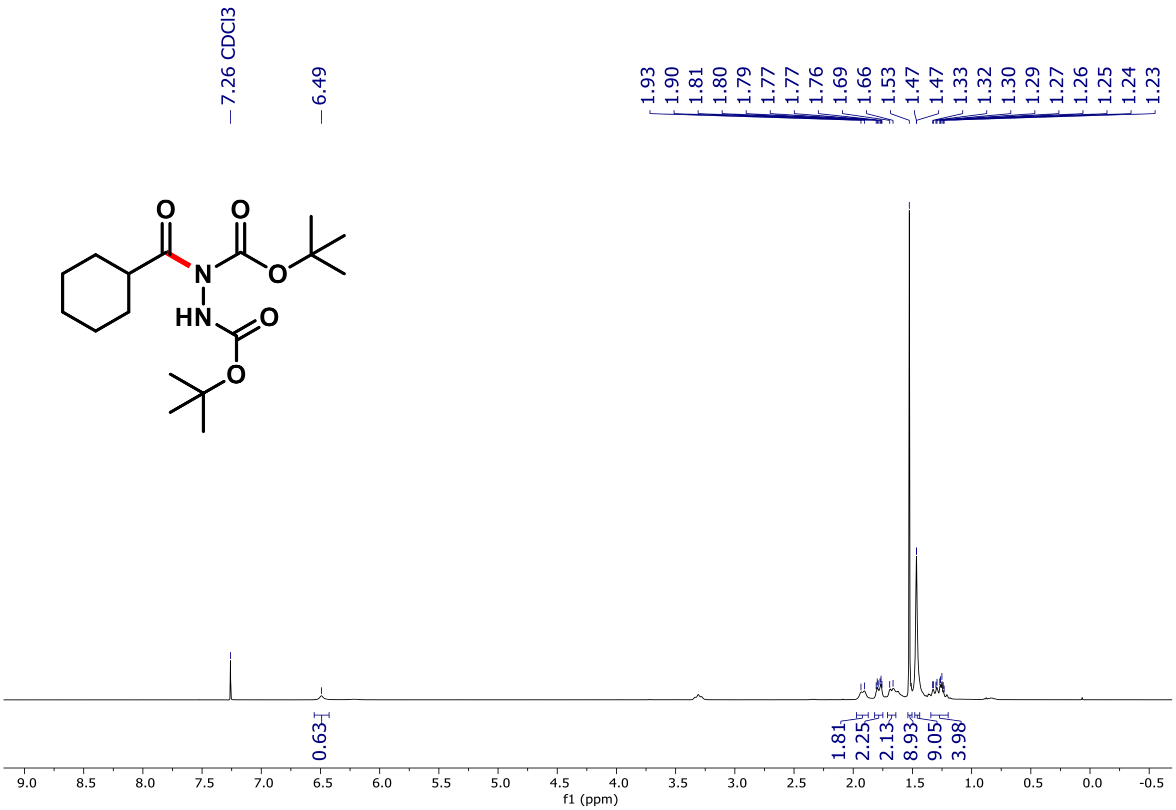

Figure S49. ^{1}H NMR of compound **3s** (400 MHz, $CDCl_3$)

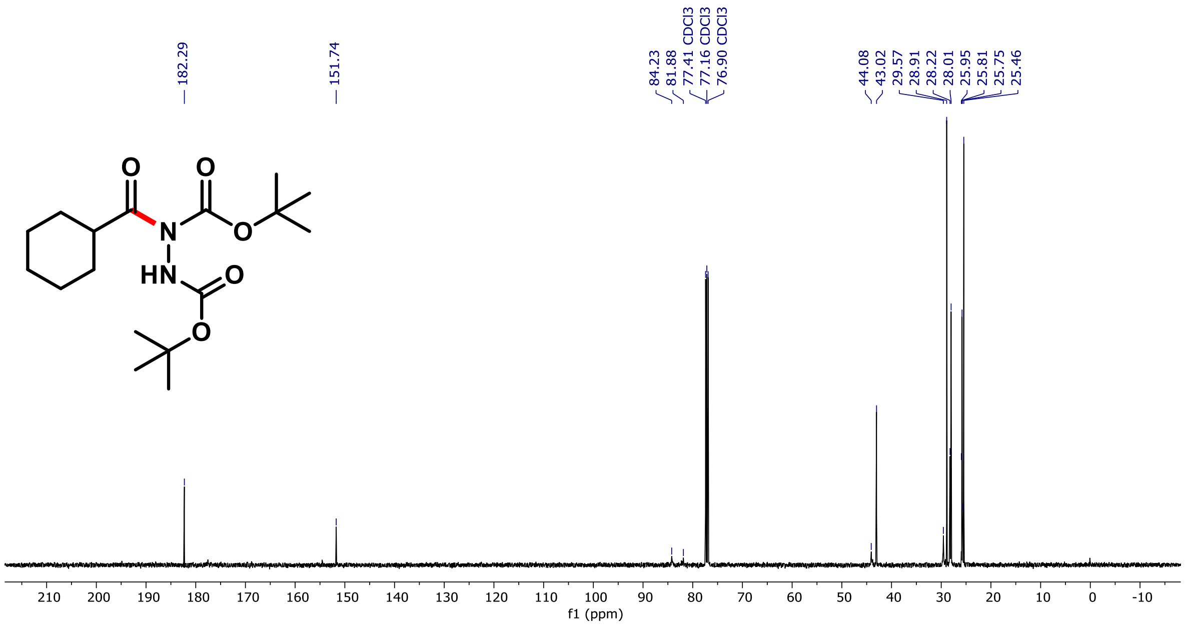

Figure S50. ^{13}C NMR of compound **3s** (101 MHz, $CDCl_3$)

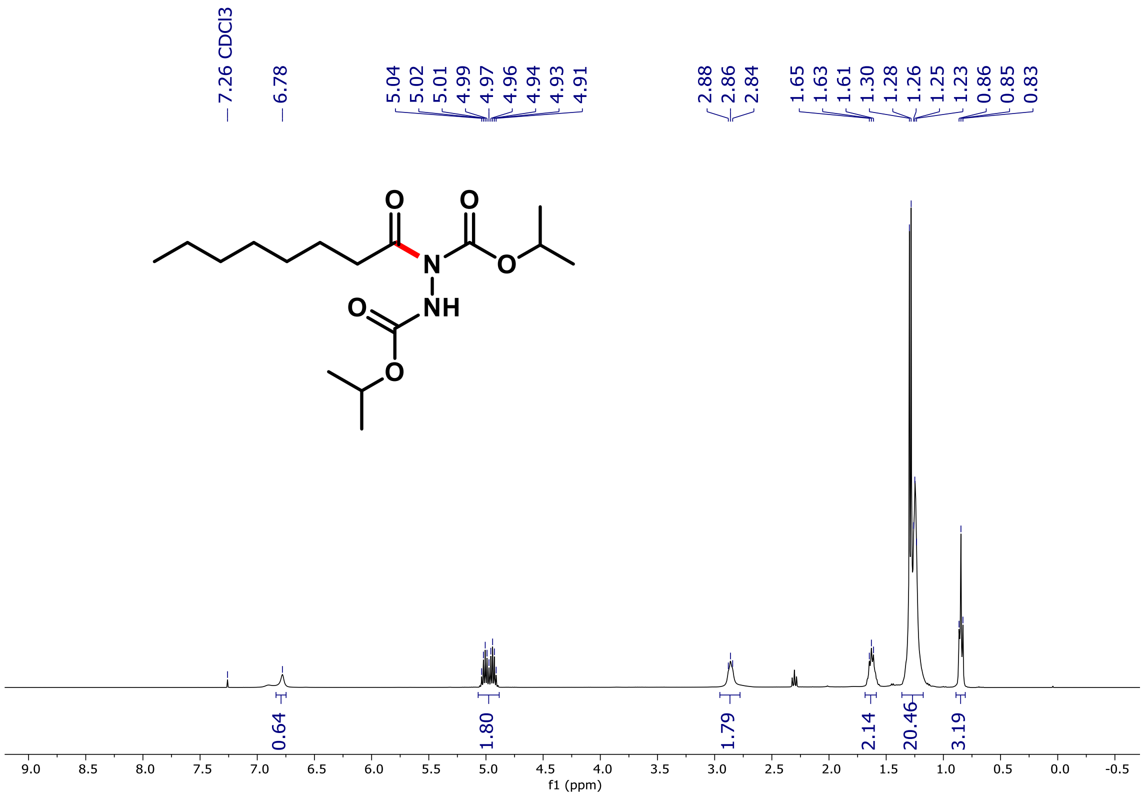

Figure S51. ^{1}H NMR of compound **3t** (400 MHz, $CDCl_3$)

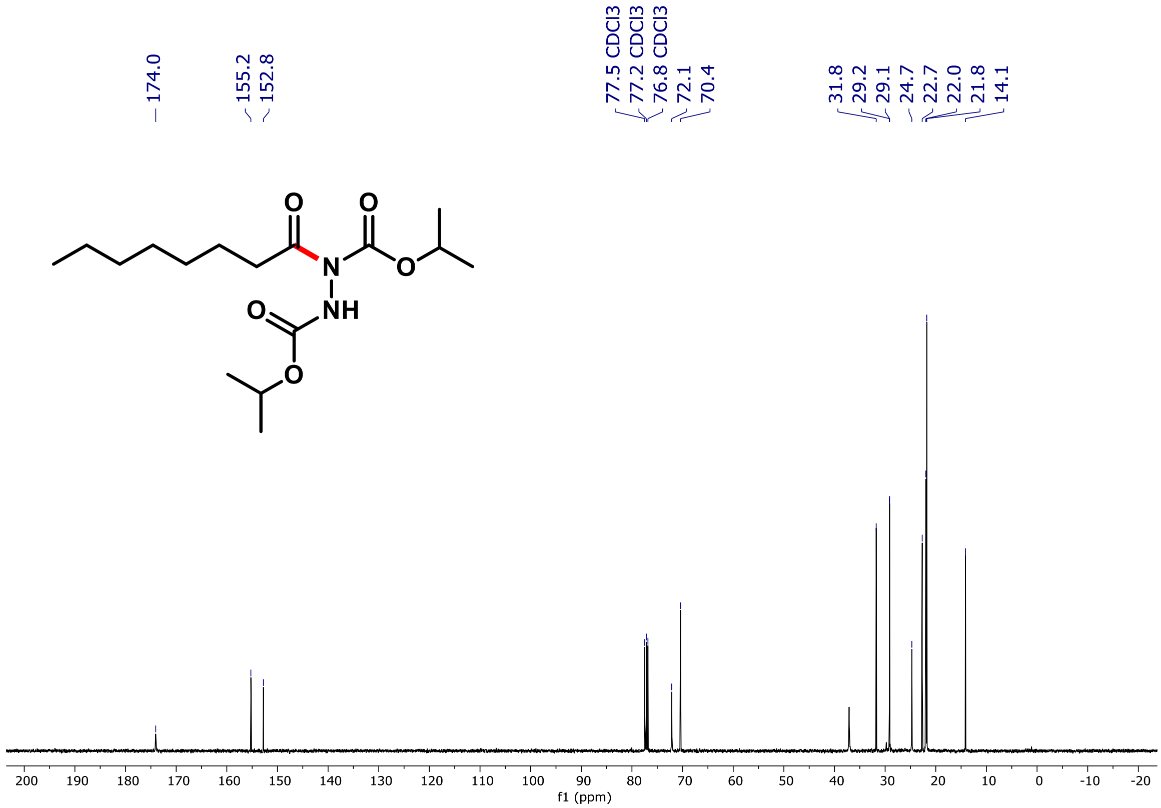

Figure S52. ^{13}C NMR of compound **3t** (101 MHz, $CDCl_3$)

References

[1] W. L. Armarego, *Purification of laboratory chemicals*, Butterworth-Heinemann, **2017**.

[2] M. Tamura, K.-i. Shimizu, A. Satsuma, *Applied Catalysis A: General* **2012**, *433*, 135-145.

[3] S. Singh, S. Sharma, A. Umar, S. K. Mehta, M. S. Bhatti, S. K. Kansal, *Journal of nanoscience and nanotechnology* **2018**, *18*, 5804-5809.

[4] M. Pera-Titus, T. Lescouet, S. Aguado, D. Farrusseng, *The Journal of Physical Chemistry C* **2012**, *116*, 9507-9516.

[5] E. Rahmani, M. Rahmani, *Industrial & Engineering Chemistry Research* **2018**, *57*, 169-178.

[6] J. Tang, S. Li, J. Xu, F. Deng, *The Journal of Physical Chemistry C* **2022**, *126*, 13485-13495.

[7] M. Muttakin, S. Mitra, K. Thu, K. Ito, B. B. Saha, *International Journal of Heat and Mass Transfer* **2018**, *122*, 795-805.

[8] J. Wang, Q. Wang, G. Wang, *Turkish Journal of Chemistry* **2022**, *46*, 1281-1290.

[9] Hu, A., Guo, J.-J., Pan, H., & Zuo, Z. Science, **2018**, 361(6403), 668-672.
